# η^6^-Benzene Tetra-Anion Complexes of Early and Late Rare-Earth Metals

**DOI:** 10.1021/jacs.5c00707

**Published:** 2025-03-21

**Authors:** Ming Liu, Yan-Cong Chen, Arpan Mondal, Huan Wang, Ming-Liang Tong, Richard A. Layfield, Fu-Sheng Guo

**Affiliations:** a Institute of Fundamental and Frontier Sciences, University of Electronic Science and Technology of China, Xiyuan Avenue 2006, Chengdu 611731, China; b Key Laboratory of Bioinorganic and Synthetic Chemistry of the Ministry of Education, School of Chemistry, IGCME, GBRCE for Functional Molecular Engineering, Sun Yat-Sen University, Guangzhou 510006, China; c Department of Chemistry, University of Sussex, Falmer, Brighton BN1 9QR, U.K.

## Abstract

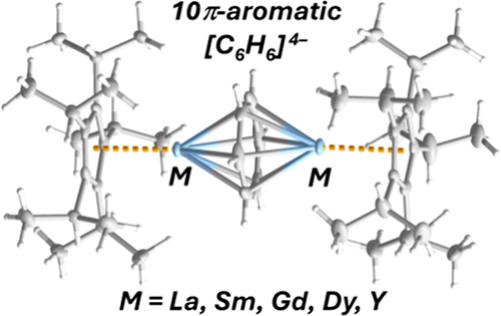

A novel synthetic route to the triple-decker benzene tetra-anion complexes [(η^5^-C_5_*^i^*Pr_5_)M(μ:η^6^:η^6^-C_6_H_6_)M(η^5^-C_5_*^i^*Pr_5_)] is reported for a range of early and late rare-earth elements, i.e., M = Y, La, Sm, Gd, and Dy (**1**_**M**_). The lanthanum complex **1**_**La**_ is the first benzene tetra-anion complex of the largest rare-earth element. Aromaticity in the 10π-electron benzene ligands is confirmed through crystallographic studies of all compounds and nucleus-independent chemical shift calculations on **1**_**Y**_ and **1**_**La**_. Analysis of the bonding in **1**_**Y**_ and **1**_**La**_ using density functional theory revealed strong covalency in the metal-benzene interactions, with very similar contributions from the metal 4d/5d orbitals, respectively, and the benzene π* orbitals. Magnetic susceptibility measurements on **1**_**Sm**_, **1_Gd_**, and **1**_**Dy**_ are also consistent with the presence of a benzene tetra-anion ligand. The origins of the appreciable exchange coupling constant of *J*_exch_ = −3.35 cm^–1^ (−2*J* formalism) in **1**_**Gd**_ are established through a computational study of the interacting magnetic orbitals. The dynamic magnetic properties of **1**_**Dy**_ are also described. The clear absence of SMM behavior in the dysprosium complex is explained using multireference calculations and an ab initio ligand-field theory description of the 4f orbitals, which clearly show that the benzene tetra-anion ligand provides a dominant equatorial contribution.

## Introduction

Organometallic sandwich and half-sandwich complexes continue to play central roles in advancing the chemistry of the rare-earth and actinide elements.^1,2^ By far the most popular ligand in f-element sandwich chemistry is the venerable cyclopentadienyl (Cp) ligand, with its plethora of substituted and isoelectronic derivatives. Significant developments include the synthesis of compounds containing metals in exotic oxidation states or with unprecedented metal–metal bonds,^3−6^ and applications of such complexes range from small-molecule activation^7−11^ to single-molecule magnets (SMMs) and molecular spin qubits.^12−18^ Cyclo-octatetraenyl ligands have also played a pivotal role in developing the field.^19−23^ More recently, other ligand types, notably η^4^-cyclobutadienyl,^24−28^ η^7^-cycloheptatrienyl^29,30^ and η^9^-cyclononatetraenyl,^31,32^ have been introduced into f-element sandwich and half-sandwich chemistry.

Another ligand type that continues to generate considerable interest in rare-earth and early actinide chemistry is η^6^-arene, especially benzene itself.^33,34^ η^6^-Arene ligands can be distinguished from other η*^n^*-ligands in f-element chemistry by their rich redox properties, resulting in complexes of the ligand in neutral^35−41^ or monoreduced forms,^42,43^ a singlet or triplet dianion,^44−53^ or even as the exotic tetra-anion.^54−59^ Indeed, rare-earth complexes of the benzene tetra-anion η^6^-[C_6_H_6_]^4–^ are currently attracting considerable attention because of the formal 10π-electron, Hückel aromatic nature of the ligand and the possibility of metal–ligand covalency with elements that conventionally engage in electrostatic bonding.^60−64^

Given the scarcity of rare-earth complexes of benzene tetra-anion ligands, we sought to prepare complexes of the type [(η^5^-C_5_*^i^*Pr_5_)M(μ:η^6^:η^6^-C_6_H_6_)M(η^5^-C_5_*^i^*Pr_5_)] where M is yttrium (**1**_**Y**_), lanthanum (**1**_**La**_), samarium (**1**_**Sm**_), gadolinium (**1**_**Gd**_) or dysprosium (**1**_**Dy**_). In complexes **1**_**M**_, the metal centers should occupy the common rare-earth oxidation state M^3+^ and the bridging η^6^-benzene ligand should be present as the tetra-anion. We note that, in the preparation of the current article, McClain et al. described some properties of **1**_**Y**_, **1**_**Gd**_ and **1**_**Dy**_ in addition to the terbium and thulium versions **1**_**Tb**_ and **1**_**Tm**_.^63^ We now focus on the synthesis and structural properties of the early rare-earth benzene tetra-anion complexes **1**_**La**_ and **1**_**Sm**_ in addition to the metal-arene bonding in **1**_**Y**_ and **1**_**La**_, while also providing deeper analysis of the magnetism and ligand-field properties of **1**_**Gd**_ and **1**_**Dy**_.

## Results and Discussion

**Scheme 1:**
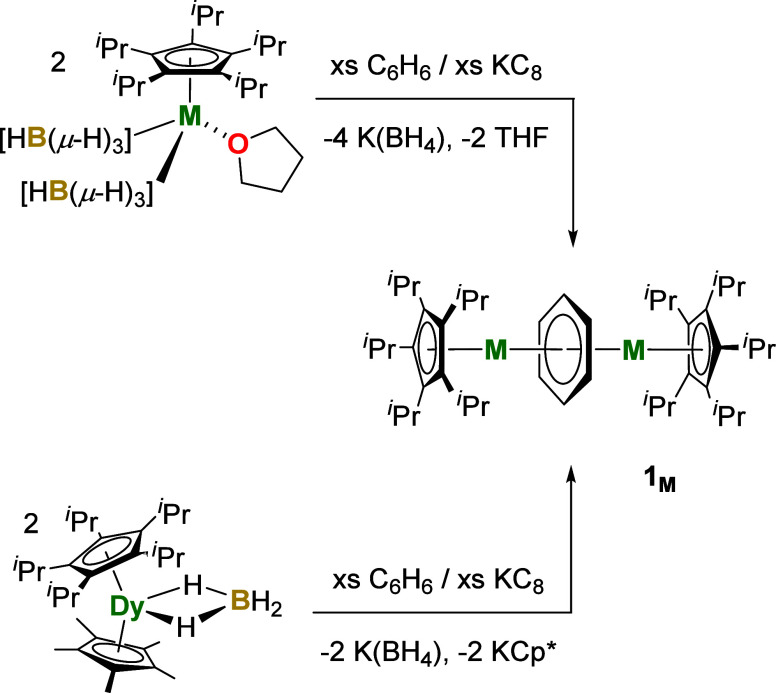
Synthesis of **1**_**M**_ with M = Y, La, Sm, Gd, and Dy

Synthesis of the target compounds was achieved according to Scheme 1 using dysprosium and two complementary methods, which differ from those described previously.^63^ In the first method, benzene was added to a mixture of [(η^5^-C_5_*^i^*Pr_5_)Dy(η^5^-Cp*)(BH_4_)] (Cp* = C_5_Me_5_) and a 10-fold excess of potassium graphite, which produced a dark brown solution after stirring for 10 days. Following workup, dark red crystals of **1**_**Dy**_ were obtained in an isolated yield of only 2%. In the second method, adding benzene to a mixture of the half-sandwich complex [(η^5^-C_5_*^i^*Pr_5_)Dy(BH_4_)_2_(THF)] and a five-fold excess of potassium graphite also formed a dark brown solution after 3 days. Following workup, dark red crystals of **1**_**Dy**_ were obtained in an improved yield of 33%.

The second synthesis method based on the novel half-sandwich complexes [(η^5^-C_5_*^i^*Pr_5_)M(BH_4_)_2_(THF)] (Figures S1–S4, S10, Tables S1, S3–S6) was therefore used to synthesize analogous compounds for the other rare-earths, allowing dark red crystals of **1**_**Y**_, **1**_**La**_, **1**_**Sm**_, and **1**_**Gd**_ to be obtained in yields of 32%, 22%, 31% and 32%, respectively. By incorporating early and late rare-earth elements, the scope of the synthetic method described here is broader than those reported previously,^63^ which typically focus on late rare-earth elements.

### Solid-State Structures

**Figure 1:**
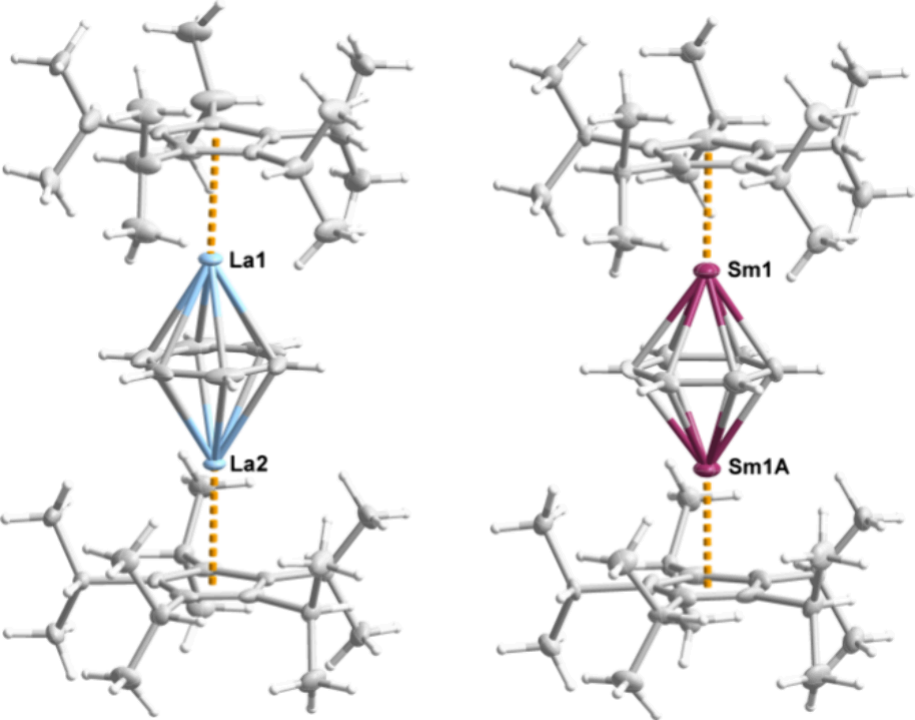
Thermal ellipsoid representation (50% probability) of the molecular structures of **1**_**La**_ and **1**_**Sm**_. Unlabeled atoms are carbon (gray) and hydrogen (white).

The molecular structures of all compounds were determined by X-ray crystallography and found to be similar, consistent with the similarities in their FTIR spectra (Figures S5–S9). The structures of **1**_**La**_ and **1**_**Sm**_ (Figure 1, Tables S2, S8, S9) are described in detail here, with **1**_**Y**_, **1**_**Gd**_ and **1**_**Dy**_ presented in the Supporting Information (Figure S11, Tables S7, S10, S11). Molecules of **1**_**La**_ and **1**_**Sm**_ are triple-decker sandwich complexes with two capping η^5^-C_5_*^i^*Pr_5_ ligands and an η^6^-C_6_H_6_ middle deck. The C_5_*^i^*Pr_5_ ligands are disordered over two sites and their positions were refined with the isopropyl groups adopting opposing orientations. The crystallographic 2-fold rotation axis is coplanar with the η^6^-benzene ligand. The near-linearity of **1**_**La**_ is reflected in the (C_5_*^i^*Pr_5_)_c_-La-(C_6_H_6_)_c_ angles of 176.521(9)/176.665(9)°, the La-(C_6_H_6_)_c_-La angle of 178.837(7)° (subscript ‘c’ denotes the ligand centroid), and the angles of 4.37(9)/2.75(9)° formed by the intersection of the cyclopentadienyl and benzene ring planes. The structure of **1**_**Sm**_ is also near-linear, with (C_5_*^i^*Pr_5_)_c_-Sm-(C_6_H_6_)_c_ and Sm-(C_6_H_6_)_c_-Sm angles of 177.49(19) and 176.41(17)°, respectively, and an angle of 2.0(5)° formed by the intersection of the cyclopentadienyl and benzene ring planes.

**Scheme 2:**
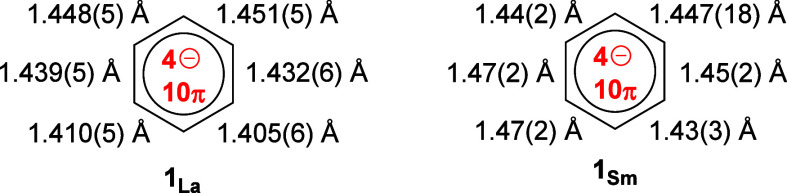
Benzene C–C Bond Lengths in **1**_**La**_ and **1**_**Sm**_

An initial indication of the extent to which the η^6^-benzene ligands in **1**_**La**_ and **1**_**Sm**_ have been reduced can be obtained by considering the C–C bond lengths. The benzene C–C distances in **1**_**La**_ span a relatively narrow range of 1.405(6)-1.451(5) (average 1.431 Å) (Scheme 2). In addition, the deviations of the carbon atoms from the mean place of the benzene ring are only 0.0003–0.0015 Å, hence the ligand is essentially planar. The structure of the η^6^-benzene ligand in **1**_**La**_ is therefore consistent with the fully delocalized, tetra-anionic form, implying that the lanthanum centers are present in the trivalent oxidation state. The benzene C–C distances in **1**_**Sm**_ adopt a similar pattern, lying in the range 1.43(3)-1.47(2) Å (average 1.45 Å), and the planarity of the ligand is reflected in the small deviations of 0.001–0.006 of the carbon atoms from the mean plane of the benzene ring. These features are also consistent with the presence of a fully delocalized benzene tetra-anion in **1**_**Sm**_. For comparative purposes, the ranges of C–C distances in **1**_**Y**_, **1**_**Gd**_ and **1**_**Dy**_ are 1.44(3)-1.47(3) Å (average 1.45 Å), 1.438(15)- 1.465(13) Å (average 1.451 Å), and 1.418(12)-1.488(13) Å (average 1.457 Å), respectively (Tables S7, S10, S11).

The La–(C_5_*^i^*Pr_5_)_c_ distances of 2.58126(19)/2.58738(19) Å in **1**_**La**_ are of limited use in assigning an oxidation state to the metal since there is, seemingly, considerable variation in the analogous distances found in related compounds. For example, divalent (C_5_*^i^*Pr_5_)_2_La has a metal-centroid distance of 2.559(5) Å whereas trivalent (C_5_*^i^*Pr_5_)_2_LaI has analogous distances of 2.585(3) Å and 2.580(3) Å.^65^ Lanthanum–Cp_c_ distances as long as 2.681–2.709 Å have also been reported for the arene-bridged complex [(η^5^-Cp^tt^_2_)_2_La(μ:η^6^:η^6^-C_6_H_6_)La(η^5^-Cp^tt^_2_)_2_]^2–^ (Cp^tt^ = 1,3-di(*tert*-butyl)cyclopentadienyl), in which both lanthanum centers are divalent.^45^ In contrast, the Sm–(C_5_*^i^*Pr_5_)_c_ distance of 2.4639(3) Å in **1**_**Sm**_ is typical for a samarium(III) metallocene, e.g., Cp*_2_Sm(X)(THF) (Cp* = C_5_Me_5_, X = Cl, I) with analogous distances of 2.43(5)-2.47(4) Å,^66^ rather than a well-defined samarium(II) metallocene such as (C_5_*^i^*Pr_5_)_2_Sm, where the distance is 2.590(5) Å.^65^ These data support the claim for a benzene tetra-anion in **1**_**Sm**_.

Turning to the metal-benzene interactions, the La–(C_6_H_6_)_c_ distances in **1**_**La**_ are, at 2.18609(18) and 2.18657(18) Å, noticeably shorter than the distances to the cyclopentadienyl centroids. Furthermore, in comparison to La–(C_6_H_6_)_c_ distances in well-defined lanthanum(III) complexes of neutral arene ligands, such as those of 2.633–2.666 Å in [(η^6^-arene)La(AlX_4_)_3_] (X = halide),^67^ a stronger metal-arene interaction with an anionic version of the ligand appears likely in **1**_**La**_. The La–(C_6_H_6_)_c_ distances in **1**_**La**_ are also shorter by 0.086–0.092 Å than those in [{(η^5^-Cp’)}_2_La(μ:η^6^:η^6^-C_6_H_6_)]^2–^ (Cp’ = C_5_H_4_SiMe_3_), which consists of two La(II) centers bridged by a benzene dianion, with La–(C_6_H_6_)_c_ distances of 2.278 Å and 2.273 Å.^42^

In the case of **1**_**Sm**_, the Sm–(C_6_H_6_)_c_ distances can be considered in light of the structures of recently reported samarium(III) complexes with benzene tetra-anion ligands.^60−62,64^ Thus, the Sm1–(C_6_H_6_)_c_ and Sm1A–(C_6_H_6_)_c_ distances are 2.0636(3) and 2.0996(3) Å, respectively, both of which are similar to those reported for a series of β-diketiminate-capped samarium(III) complexes of benzene tetra-anions, i.e., 2.075(2)-2.109(1) Å.^60,61^ This comparison strongly supports the presence of a benzene tetra-anion ligand in **1**_**Sm**_. The structural properties of the metal-arene interactions in **1**_**Sm**_ are mirrored in the structures of **1**_**Y**_, **1**_**Gd**_ and **1**_**Dy**_ (Figures S11, Tables S7, S10, S11), with M–(C_6_H_6_)_c_ distances of 1.974(9)/2.000(9) Å, 2.055(6)/2.022(6) Å, and 1.969(5)/2.019(6) Å, respectively, consistent with the analysis presented by by McClain et al.^63^

Considering the five **1**_**M**_ compounds, as expected based on the decreasing radius of the M^3+^ cation across the series, the M–(C_6_H_6_)_c_ distances decrease as 2.18609(18)/2.18657(18) Å, 2.0636(3)/2.0996(3) Å, 2.055(6)/ 2.022(6) Å, 1.969(5)/ 2.019(6) Å and 1.974(9)/ 2.000(9) Å, respectively. A similar trend is found for the M···M separations, which are 4.3724(3), 4.1576(7) Å, 4.0707(2), 3.9798(3) and 3.9673(8) Å in **1**_**La**_, **1**_**Sm**_, **1**_**Gd**_, **1**_**Dy**_, and **1**_**Y**_, respectively.

### NMR Spectroscopy

Turning to the solution-phase structures, the ^1^H NMR spectrum of **1**_**La**_ in C_6_D_6_ consists of a resonance at 3.79 ppm for the benzene protons in addition to signals for the isopropyl groups, and the ^13^C{^1^H} NMR spectrum has a resonance at 67.2 ppm for the benzene carbons in addition to the other resonances (Figures S23, S24). The ^1^H NMR spectrum of **1**_**La**_ is temperature-independent up to 80 °C (Figure S26). The upfield ^1^H and ^13^C NMR resonances for the benzene ligand in **1**_**La**_ are consistent with shielding of the nuclei by a 10π aromatic ring current, with the equivalent resonances observed for **1**_**Y**_ at δ(^1^H) = 3.75 ppm and δ(^13^C) = 59.2 ppm, respectively, suggesting similar behavior (Figures S19–S21). The ^89^Y NMR spectrum of **1**_**Y**_ features a single resonance at – 103 ppm (Figure S22), in agreement with the previously reported spectrum of this compound.^63^

Overall, the appearance of the NMR spectra for **1**_**La**_ is consistent with diamagnetism. However, the appearance of diamagnetism could arise two La(III) centers and a benzene tetra-anion or two 5d^1^ La(II) centers strongly exchange coupled via a benzene dianion. The latter scenario was proposed to account for the appearance of diamagnetism in the ^1^H NMR spectrum of [(Cp’_2_Ln)_2_((μ:η^6^:η^6^-C_6_H_6_)]^2–^, a complex found to be weakly paramagnetic through magnetic susceptibility measurements, which was interpreted in terms of the La(II) centers coupling strongly with a triplet benzene dianion ligand.^45^ The possibility of paramagnetism in **1**_**La**_ was excluded by measuring the molar magnetic susceptibility (χ_M_) in a DC field of 10 kOe, which produced a negative response (Figure S53), which effectively confirms the presence of a benzene tetra-anion in this complex.

As highlighted recently by Harder et al. in their study of a samarium benzene tetra-anion complex,^61^^1^H NMR spectra of samarium(III) compounds feature relatively sharp resonances, whereas as those of samarium(II) complexes display severe line broadening owing to the strong paramagnetic contribution. Not only does the ^1^H NMR spectrum of **1**_**Sm**_ consist of sharp resonances for all environments (Figures S27, S28), the chemical shift corresponding to the benzene protons is, at δ = 21.13 ppm, very similar to those reported recently for the β-diketiminate-capped samarium(III) benzene tetra-anion complexes.^60−62^ It has also been noted that the ^13^C{^1^H} NMR spectra of decamethylsamarocene in the formal +2 and +3 oxidation states, and Lewis adducts thereof, can provide an indication of the formal metal oxidation state.^68,69^ Thus, the relatively downfield resonance of δ(^13^C) = 132.0 ppm for the ring carbons and the relatively upfield resonance of δ(^13^C) = 35.6 ppm for the ring-bound methine carbons in **1**_**Sm**_ are consistent with the presence of Sm^3+^ (Figures S29, S30). In addition, the isopropyl methyl group occur at δ(^13^C) = 22.8 and 19.4 ppm, and the benzene carbon atoms occur at δ(^13^C) = 3.1 ppm.

### Electronic Structure and Bonding Analysis

Insight into the electronic structure and bonding in compounds **1**_**M**_ was obtained using UV/vis/NIR spectroscopy, complemented by a DFT (density functional theory) analysis of the metal–ligand bonding in diamagnetic **1**_**Y**_ and **1**_**La**_, and time-dependent DFT (TD-DFT) calculations. The bonding analysis considered the full molecules using the experimental geometries obtained from the crystal structures, with the hydrogen atoms positions being optimized. Full details are provided in the Supporting Information.

**Figure 2:**
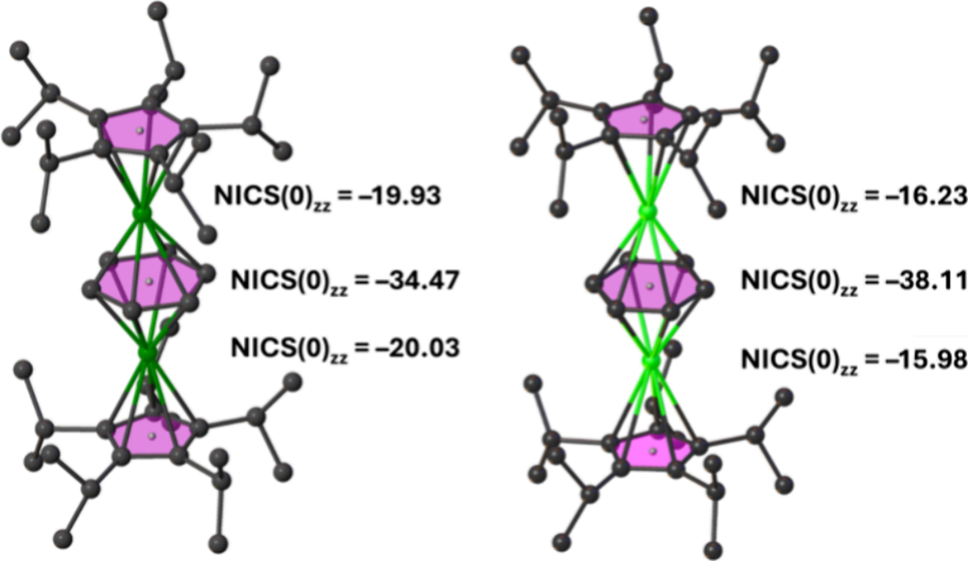
NICS(0) calculations for **1**_**Y**_ (left) and **1**_**La**_ (right).

First, the aromaticity of the benzene and cyclopentadienyl ligands in **1**_**Y**_ and **1**_**La**_ was established with nucleus-independent chemical shift (NICS) calculations using dummy atoms at the centers of the rings (Figure 2). The [C_5_*^i^*Pr_5_]^−^ ligands in **1**_**Y**_ have NICS(0) values of –19.93 and –20.03, and the [C_6_H_6_]^4–^ ligand has a NICS(0) of –34.47. In **1**_**La**_, the [C_5_*^i^*Pr_5_]^−^ ligands have NICS(0) values of –16.23 and –15.98 and the [C_6_H_6_]^4–^ ligand has a NICS(0) of –38.11. The appreciable negative NICS(0) values in **1**_**Y**_ and **1**_**La**_ are indicative of 6π aromatic character for the cyclopentadienyl ligands and 10π aromatic character for the benzene tetra-anion ligand.

**Figure 3:**
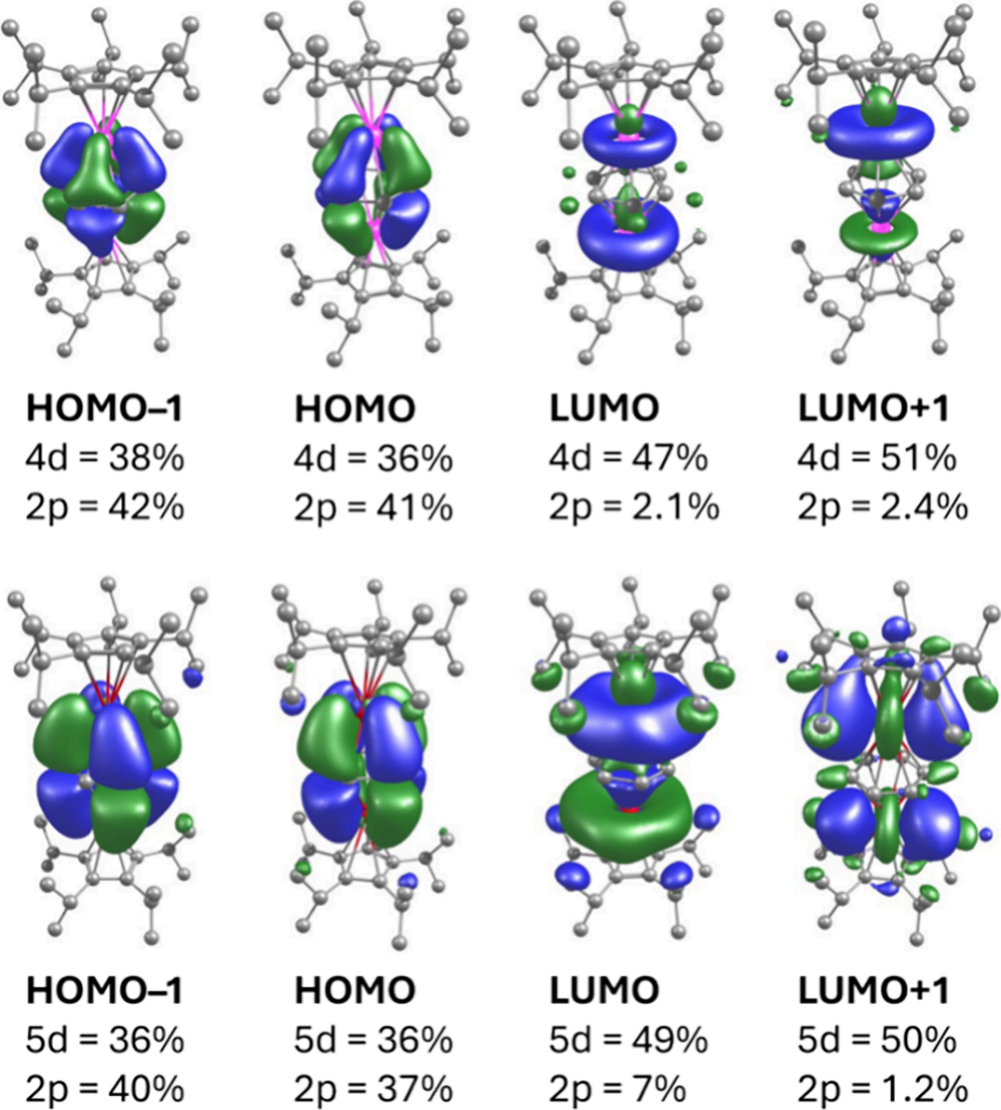
Selected frontier MOs for **1**_**Y**_ (upper) and **1**_**La**_ (lower) with associated compositions (isosurface value = 0.04).

The calculated frontier MOs for **1**_**Y**_ and **1**_**La**_ correspond to the metal-benzene interactions and are qualitatively similar, with the HOMO (highest-occupied MO) and HOMO–1 consisting of δ-type overlap between the metal d-orbitals and the benzene tetra-anion π* orbitals (Figure 3). In both molecules, the two highest-occupied MOs are composed of approximately equal contributions from the metal d (36–38%) and carbon 2p (37–42%) orbitals. The extensive orbital overlap within the HOMO and HOMO–1 reflects a high degree of covalency, which explains the short M–(C_6_H_6_)_c_ distances observed in the solid-state structures. The LUMO (lowest-unoccupied MO) and LUMO+1 in **1**_**Y**_ and **1**_**La**_ are essentially metal-based d-orbitals, with minimal contribution from the carbon 2p orbitals of the benzene ligand. Comparing **1**_**Y**_ and **1**_**La**_, it is noteworthy that, despite the appreciable difference in the radii of La^3+^ and Y^3+^ and the more diffuse nature of the 5d orbitals in the former ion, the bonding interactions are very similar in terms of orbital composition. The overall MO picture in **1**_**Y**_ and **1**_**La**_ is therefore reminiscent of other rare-earth complexes of benzene tetra-anion ligands^60−62,64^ and the much more extensive series of arene-bridged diuranium compounds, for which metal–ligand δ-type bonding involving various combinations of uranium 5f and 6d orbitals has been established.^40,47,50,52,54−56,62,70^

**Figure 4:**
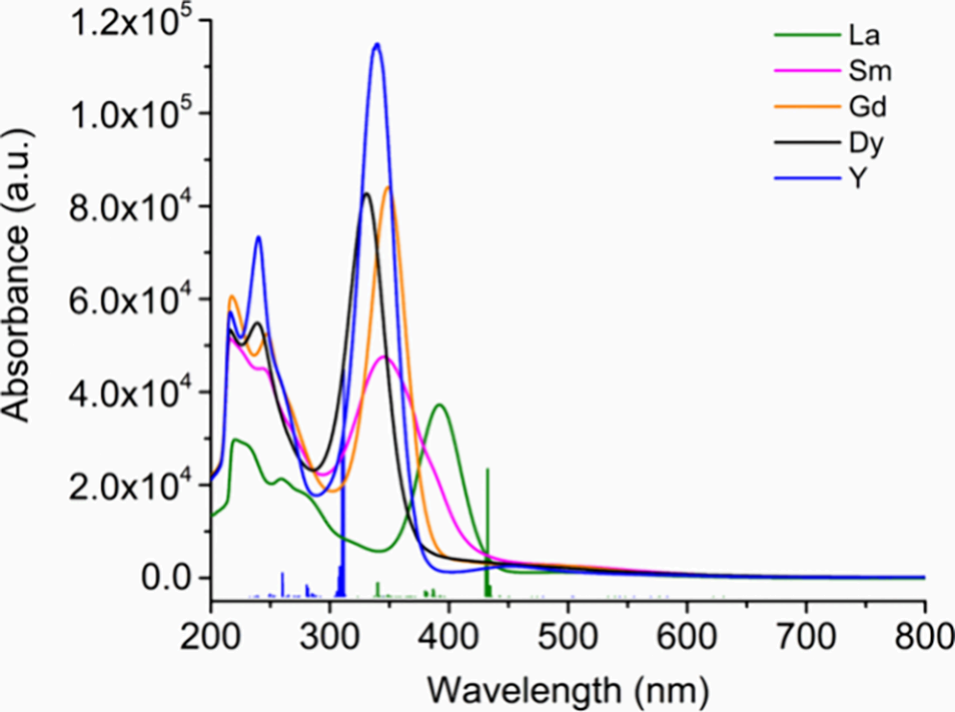
UV/vis/NIR spectra of **1**_**M**_ (M = Y, La, Sm, Gd, and Dy) in hexane. Solid vertical lines correspond TD-DFT calculated transitions for **1**_**Y**_ and **1**_**La**_.

To connect the calculated MOs for **1**_**Y**_ and **1**_**La**_ to the experimental electronic structure, we measured the UV/vis/NIR spectra of all **1**_**M**_ compounds in the range 200–1000 nm and then performed TD-DFT analyses of the yttrium and lanthanum versions. The spectra of the new compounds **1**_**La**_ and **1**_**Sm**_ are similar to those of **1**_**Y**_, **1**_**Gd**_ and **1**_**Dy**_ (Figure 4, Figures S31–S52). The UV/vis/NIR spectrum of **1**_**La**_ consists of a strong absorption at 392 nm with two additional absorptions at 220 and 260 nm. For **1**_**Sm**_, three absorptions of comparable intensity occur at 216, 244, and 345 nm. The spectra of **1**_**Y**_, **1**_**Gd**_ and **1**_**Dy**_ reported previously were only measured in a spectral window of 300–700 nm and were found to contain a single absorption in the region 300–350 nm.^63^ While we also find dominant absorptions at 340, 349, and 331 nm in the spectra of **1**_**Y**_, **1**_**Gd**_ and **1**_**Dy**_, at higher energies we also find additional significant absorptions. For **1**_**Y**_, these additional absorptions occur at 216/240 nm, and for **1**_**Sm**_, **1**_**Gd**_ and **1**_**Dy**_ they occur at 216/244 nm, 216/247 nm and 216/239 nm, respectively.

Analysis of the UV/vis/NIR transitions observed for **1**_**Y**_ and **1**_**La**_ was aided by TD-DFT calculations (see Supporting Information for computational details). The close similarities in the spectra of all five compounds suggest that the results for **1**_**Y**_ and **1**_**La**_ should also apply to the other members of the series. The calculations accurately reproduce the spectrum of **1**_**La**_ and **1**_**Y**_. The major absorption observed for the lanthanum complex at 392 nm corresponds to a transition calculated at 436 nm and is described by transitions from the HOMO–1 to the metal-based orbitals LUMO+1 and LUMO+6. The analogous absorption at 340 nm for **1**_**Y**_ is calculated to occur at 315 nm, corresponding to HOMO–1/LUMO+3 and HOMO/LUMO+2 transitions, with these LUMOs also being metal-based. The previously unobserved higher-energy absorptions in **1**_**Y**_ and **1**_**La**_ around 216–260 nm correspond to transitions from the HOMO–1 and HOMO to much higher-lying orbitals, which are mostly yttrium 5d-character in **1**_**Y**_ and lanthanum 4f character in **1**_**La**_.

### Magnetic Properties

Evidence for a benzene tetra-anion ligand in **1**_**Gd**_ can also be obtained through magnetic susceptibility measurements since the well-isolated ^8^S_7/2_ ground state of gadolinium(III) facilitates analysis of the magnetism using a standard spin Hamiltonian. Thus, the temperature dependence of χ_M_*T*, where χ_M_ is the molar magnetic susceptibility, was measured in an applied field of 1000 Oe in the range 2–300 K. The χ_M_*T*(*T*) profile for **1**_**Gd**_ contrasts markedly to that of conventional weakly exchange-coupled gadolinium dimers,^71^ which typically show room-temperature χ_M_*T* close to the theoretical value of 15.76 cm^3^ K mol^–1^ for two uncoupled Gd^3+^ ions, and a slight decrease in χ_M_*T* with temperature on approaching 2 K (Figure S55). For **1**_**Gd**_, χ_M_*T* at 300 K is an unusually low 13.31 cm^3^ K mol^–1^, with χ_M_*T* decreasing appreciably as the temperature approaches 120 K. A more rapid decrease in χ_M_*T* occurs at lower temperatures and, remarkably, an extremely low value of 0.21 cm^3^ K mol^–1^ is reached at 2 K. A good fit of the χ_M_*T*(*T*) data was obtained using the isotropic spin Hamiltonian *Ĥ* = – *J*_exch_*Ŝ*_1_ · *Ŝ*_2_ + *g*μ_B_*Ŝ*_*z*_ · *B*, where *J*_exch_ is the exchange coupling constant, *Ŝ*_*n*_ denotes a spin of 7/2 for each Gd^3+^ ion (necessitating a benzene tetra-anion for charge balance), and *g* = 2.01(1). The analysis for **1**_**Gd**_ yielded *J*_exch_ = –3.35 cm^–1^, indicating strong antiferromagnetic exchange. Although exchange-coupled gadolinium dimers with bridging arene tetra-anion ligands are very rare, we note that *J*-values in the region of –0.65 cm^–1^ (−2*J* formalism) have been reported for a related compound containing a biphenyl tetra-anion.^59^ The much stronger exchange coupling in **1**_**Gd**_ is most likely due to a higher degree of overlap between the relevant magnetic orbitals. In addition, the exchange coupling constant determined through our analysis of **1**_**Gd**_ is slightly larger than the value of – 2.94(2) cm^–1^ reported elsewhere,^63^ although no detailed study of the spin density or magnetic orbitals was presented.

**Figure 5:**
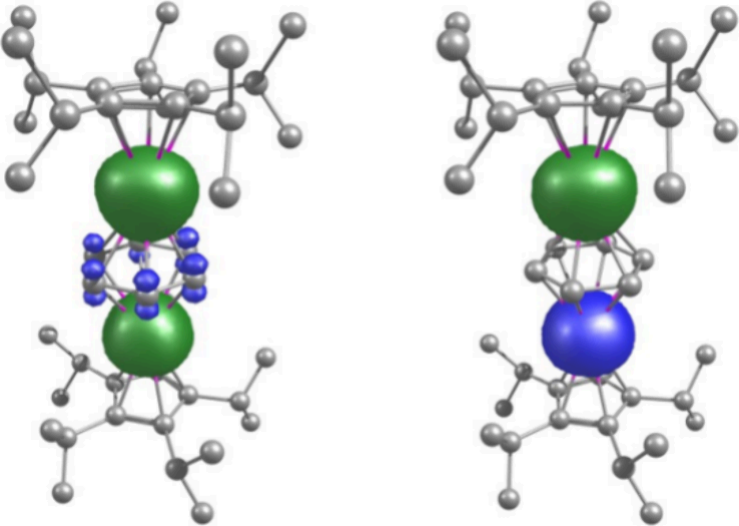
Spin density plots for the high-spin (left) and broken-symmetry (right) states for **1**_**Gd**_. Green and blue indicate positive and negative spin density, respectively (isosurface value = 0.002).

**Figure 6:**
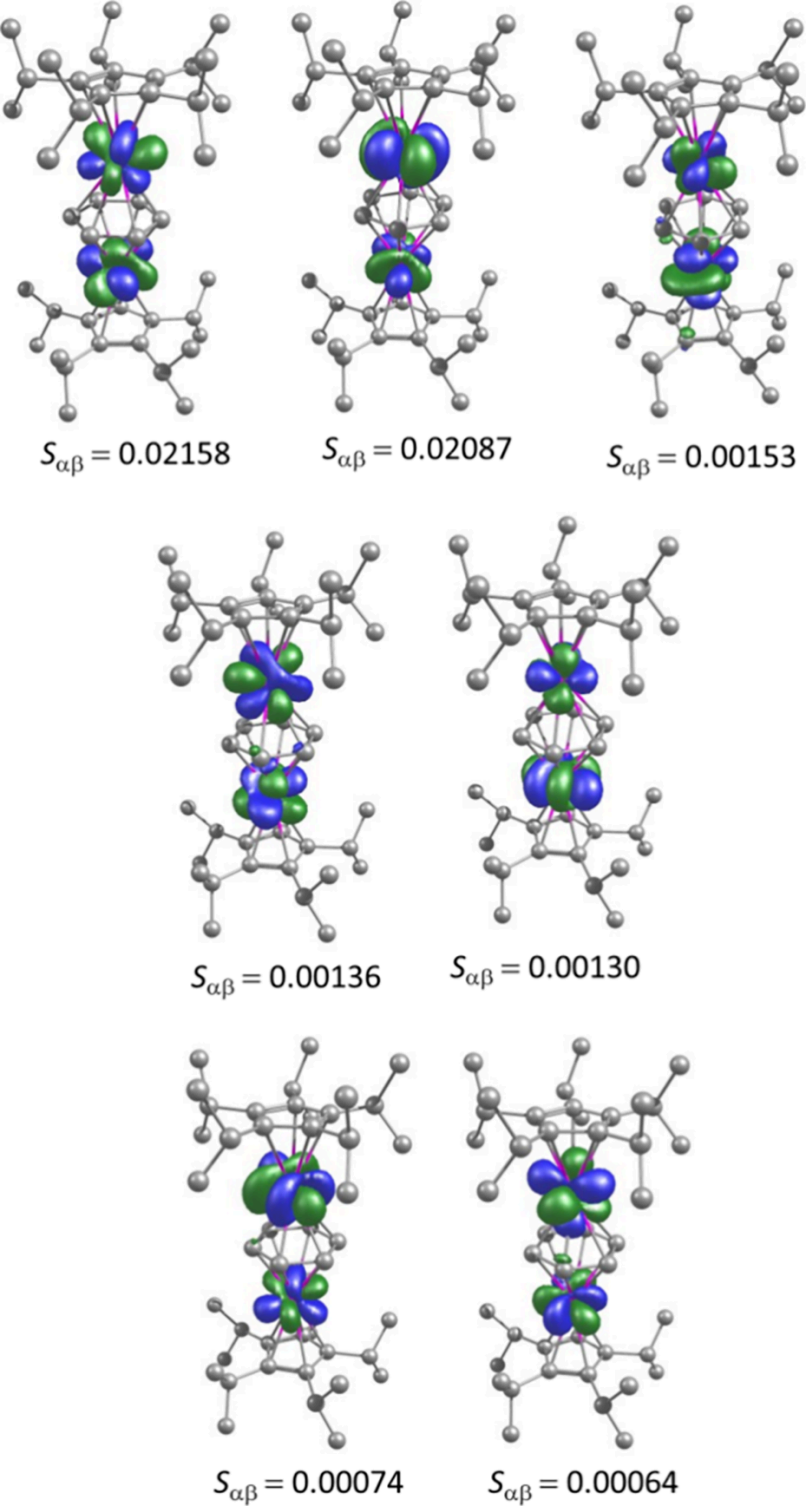
Interacting magnetic orbitals and overlap integrals (*S*_αβ_) for **1**_**Gd**_.

To gain insight into the magnetic exchange coupling between two gadolinium centers in **1**_**Gd**_, we performed DFT calculations in combination with the broken symmetry (BS) approach using ORCA 5.0.2.^72,73^ The calculated energies for the high-spin and broken-symmetry state and the exchange coupling constant are summarized in Table S14. The calculated BS-DFT exchange coupling constant shows an antiferromagnetic exchange interaction with *J*_exch_ = –2.86 cm^–1^, in good agreement with the experimental value. The spin density analysis (Figure 5, Table S15) shows negative spin density on the carbon atoms of the [C_6_H_6_]^4–^ ligand, indicating spin polarization from gadolinium and increasing spin delocalization as well as exchange interactions between the gadolinium centers. Furthermore, the magnetic 4f orbitals on gadolinium show non-negligible overlap integrals through the [C_6_H_6_]^4–^ ligand (Figure 6), which are responsible for the antiferromagnetic exchange interaction.

Susceptibility measurements on **1**_**Sm**_ were inconclusive regarding the oxidation state of the metal. However, the strong dependence of χ_M_*T* on temperature and the fact that χ_M_*T* is still increasing rapidly above 300 K (Figure S54) is consistent with the characteristic non-Curie behavior of Sm^3+^ (4f^5^, ^6^H_5/2_ ground multiplet), which is known to have low-lying excited states that can be populated at room temperature.^74^ Magnetic susceptibility measurements on **1**_**Dy**_ also show a strong dependence of χ_M_*T* on temperature, particularly below 80 K, where the decrease in χ_M_*T* is rapid (Figure S57). At 300 K, χ_M_*T* for **1**_**Dy**_ is 27.96 cm^3^ K mol^–1^, which is close to the theoretical value for two uncoupled Dy^3+^ ions (4f^9^, ^6^H_15/2_ ground multiplet). At 2 K, however, the value of χ_M_*T* drops to 1.87 cm^3^ K mol^–1^, indicating antiferromagnetic exchange. Simulation of the susceptibility of **1**_**Dy**_ using the PHI software,^75^ the Hamiltonian *Ĥ* = –2*J*_tot_ · (*S*_Dy1_ · *S*_Dy2_) + *B*_0_^2^*C*_0_^2^ + *B*_0_^4^*C*_0_^4^ + *B*_0_^6^*C*_0_^6^ + *zJ*′, and the crystal field parameters stated in Table S20 yielded an exchange coupling constant of *J*_*tot*_ = – 0.78 cm^–1^.

**Figure 7:**
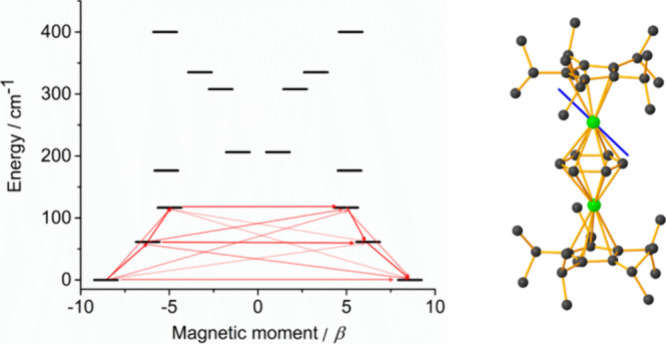
Calculated orientation of the easy axis of magnetization (blue line) for the ground KDs in **1**_Dy_. Stronger red arrows indicate larger absolute values of the transition magnetic moment matrix elements between the respective states. Transitions involving higher-energy states not involved in the relaxation mechanism are omitted for clarity.

While the onset of magnetic blocking (i.e., SMM behavior) could also contribute to the low-temperature susceptibility of **1**_**Dy**_, such an occurrence would normally occur in the form of a precipitous drop in χ_M_*T* rather than the observed gradual decrease. To confirm this, AC susceptibility measurements and multireference ab initio calculations – which we note have not previously been described – were performed on **1**_**Dy**_. The absence of SMM behavior in **1**_**Dy**_ was quickly revealed by measuring the temperature dependence of the imaginary component of the AC susceptibility, i.e., χ′′(*T*), which produced zero response up to 100 K in AC frequencies ranging from 1 to 1000 Hz (Figures S59–S62). This observation implies that, despite the axial symmetry of **1**_**Dy**_ and the cyclopentadienyl ligands coinciding with the molecular symmetry axis, an appreciable equatorial ligand field should be present in this molecule. To provide a theoretical basis for this idea, the SINGLE_ANISO routine was applied to **1**_**Dy**_ to determine the crystal-field parameters and the energy spectrum of the Dy^3+^ centers, along with the *g*-tensors of the Kramers doublets (KDs) in the ground multiplet and the associated wave function composition of each KD. As shown in Figure 7, the easy axis of magnetization in the ground KD on each Dy^3+^ center deviates from the molecular symmetry axis by approximately 43°, meaning that an axial crystal field is not dominant in **1**_**Dy**_. This is confirmed by the calculated *g*-tensors associated with the ground KD, which are *g*_*x*_ = 0.19, *g*_*y*_ = 0.45 and *g*_*z*_ = 17.15, confirming the appreciable equatorial contribution (Table S16, S17). The ground KD is also a strong admixture of several different *M*_*J*_ wave functions. Thus, the weak anisotropy in **1**_**Dy**_ when compared to a highly axial SMM, such as [(η^5^-C_5_*^i^*Pr_5_)Dy(η^5^-Cp*)]^+^,^14^ means that there is effectively no thermal barrier to flipping of the magnetic dipole, and the main relaxation route is a barrier-crossing transition within the ground KD (Figure 7, Table S18).

**Figure 8:**
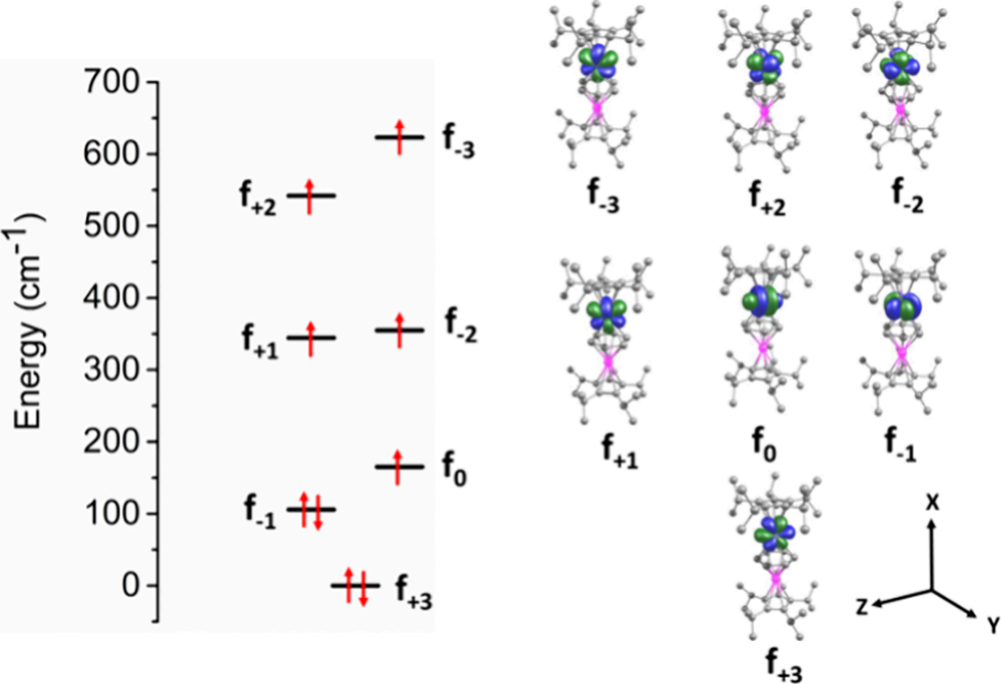
AILFT 4f-orbital splitting for **1**_**D**y_ obtained from CASSCF/NEVPT2 calculations and corresponding 4f orbitals.

To link the relatively low magnetic anisotropy in **1**_**Dy**_ to the energies of the 4f orbitals, we analyzed the computed f-orbital splitting obtained from CASSCF/NEVPT2 calculations according to ab initio ligand-field theory (AILFT).^76^ With this approach, the general set of seven 4f orbitals is denoted in simplified format according to *m*_*l*_ values: 4f_*z*^3^_ and 4f_*xz*^2^_/4f_*yz*^2^_ are represented as 4f_0_ and as 4f_±1_, respectively, and 4f*_*xyz*_*/4f_*z*(*x*^2^–*y*^2^)_ and 4f_*y*(3*x*^2^–*y*^2^)_/4f_*x*(*x*^2^–3*y*^2^)_ are represented as 4f_±2_ and 4f_±3_, respectively (Figure 8). It has been shown by Gil and Aravena that high-performance SMM behavior for the oblate ion Dy^3+^ should occur when there is a large energy separation between the axially oriented 4f_0_/4f_±1_ orbitals and the equatorially oriented 4f_±2_/4f_±3_ orbitals.

The AILFT orbital analysis of **1**_**Dy**_ reveals a substantial degree of mixing between the 4f orbitals, resulting in large energy splitting within each 4f pair, i.e., 623 cm^–1^, 141 cm^–1^, and 205 cm^–1^ for 4f_±3_, 4f_±2_ and 4f_±1_, respectively. The large splitting of the 4f_±3_ pair is likely to be a consequence of a strong equatorial ligand field exerted by the benzene tetra-anion ligand since these orbitals are sensitive to such interactions, and the carbon donor atoms in this ligand are much closer to the Dy^3+^ ions than those in the cyclopentadienyl ligands. The ab initio calculated crystal parameters for **1**_**Dy**_ agree with this analysis since the equatorial *B*_*q*_^*k*^ values are comparable in magnitude to the analogous values of the axial parameters (Table S19). Furthermore, in a linear complex such as **1**_**Dy**_, the energy of each f _± *n*_ block depends on the angular overlap model (AOM) parameters, where the 4f_0_, 4f_±1_, 4f_±2_ and 4f_±3_ orbitals are associated with e_σ_, e_π_, e_δ_, and e_φ_ parameters. In **1**_**Dy**_, the 4f_0_, and 4f_±1_ orbitals appear at lower energy compared to the 4f_±2_ orbitals, indicating that e_δ_ dominates over e_σ_ and e_π_, which is the opposite of the requirement for high-performance Dy^3+^ SMM. This observation is consistent with the properties of the HOMO calculated for **1**_**La**_, which shows delta bond formation consisting of metal 4d character and the π* orbitals of [C_6_H_6_]^4–^, which should result in the 4f_±2_ orbitals being relatively high in energy.

## Conclusions

In summary, a novel and seemingly broad-scope method for the synthesis of early and late rare-earth element complexes of benzene tetra-anion ligands has been developed. The linear triple-decker complexes [(η^5^-C_5_*^i^*Pr_5_)M(μ:η^6^:η^6^-C_6_H_6_)M(η^5^-C_5_*^i^*Pr_5_)] (**1**_**M**_), where M = Y, La, Sm, Gd and Dy, are isostructural and feature much stronger interactions between the metal and the [μ:η^6^:η^6^-C_6_H_6_]^4–^ middle decks than with the capping [η^5^-C_5_*^i^*Pr_5_)]^−^ ligands. The flat nature and equal C–C bond lengths in the benzene tetra-anion ligands, which are elongated relative to benzene itself, imply 10π aromatic character, which is supported with NICS calculations on **1**_**Y**_ and **1**_**La**_. Strong, δ-type covalent bonding between the metal and benzene ligands was established using DFT calculations on **1**_**Y**_ and **1**_**La**_. Calculations of the interacting magnetic orbitals in **1_Gd_** revealed appreciable overlap integrals for this system, providing an explanation of the experimentally determined antiferromagnetic exchange coupling constant of *J*_exch_ = –3.35 cm^–1^. Magnetic measurements on **1_Dy_** and a detailed series of theoretical calculations have provided important insight into the ligand-field properties of the benzene tetra-anion ligand. Most notably, and in contrast to the well-established axial crystal field originating from cyclopentadienyl ligands, the [μ:η^6^:η^6^-C_6_H_6_]^4–^ ligand in **1_Dy_** provides a strong equatorial crystal field, which effectively suppresses slow magnetic relaxation. This effect originates from the interactions of the ligand with specific dysprosium 4f orbitals, as established with the AILFT analysis.

With the broad-scope synthetic route to **1**_**M**_ and their electronic structure and bonding now established, our future work will focus on the reducing ability of the benzene tetra-anion ligands. We envisage that **1**_**M**_ can be used as surrogates for, or ‘masked’ versions of, rare-earth elements in the monovalent oxidation state.

## Data Availability

Additional research data supporting this publication are available as supporting information at DOI: 10.25377/sussex.28569206.

## Supporting Information Available

Synthesis, spectroscopic characterization, crystallography details, magnetic measurements, and computational details (PDF)
