# Supplementary material for: η6-Benzene Tetra-Anion Complexes of Early and Late Rare-Earth Metals
Source: J Am Chem Soc. 2025 Mar 21;147(13):11359–67. doi: 10.1021/jacs.5c00707 (PMC11969546; doi:10.1021/jacs.5c00707)
Supplement: Supplementary file 1 — ja5c00707_si_001.pdf [file ja5c00707_si_001.pdf]

Supporting Information for

η^6-Benzene Tetra-anion Complexes of Early and Late Rare-Earth Metals

Ming Liu,[a] Yan-Cong Chen,[b] Arpan Mondal,[c] Huan Wang,[a]
Ming-Liang Tong,*[b] Richard A. Layfield,*[c] and Fu-Sheng Guo*[a]

a. Institute of Fundamental and Frontier Sciences, University of Electronic Science and Technology of China, Xiyuan Avenue 2006, Chengdu 611731, China.
E-mail: guofush@hotmail.com

b. Key Laboratory of Bioinorganic and Synthetic Chemistry of the Ministry of Education, School of Chemistry, IGCME, GBRCE for Functional Molecular Engineering, Sun Yat-Sen University, Guangzhou 510006, China.
E-mail: tongml@mail.sysu.edu.cn

c. Department of Chemistry, University of Sussex, Falmer, Brighton, BN1 9QR, U.K.
E-mail: R.Layfield@sussex.ac.uk

Contents

1. General Considerations and Synthesis Procedures S1-S3
2. FTIR spectroscopy S4-S8
3. X-ray crystallography S9-S18
4. NMR Spectroscopy S19-S26
5. UV/vis/NIR Spectroscopy S27-S37
6. Magnetic Measurements S38-S43
7. Computational Details S44-S56
8. References S57-S58

General Considerations

All experiments were conducted under rigorous anhydrous, anaerobic conditions under an atmosphere of purified argon (99.9995%) using standard Schlenk line or glovebox techniques. Solvents were purified by a MIKROUNA solvent purification system and dried over activated 4 Å molecular sieves before use. C_6D_6 was refluxed over CaH_2 for at least one week and stored in an ampoule under argon. $NaCp^{iPr5}$, $M(BH_4)_3(THF)_3$ (M = Y, La, Sm, Gd), $[(Cp^{iPr5})Dy(BH_4)_2(THF)]$, $[(Cp^{iPr5})Dy(Cp^*)BH_4]$ and KC_8 were prepared according to literature procedures.[1,2]

Elemental analyses were performed on a Vario Micro Cube automatic element analyzer. FTIR spectra were recorded on a Bruker Alpha II FTIR spectrometer equipped with a Platinum ATR module. UV/vis/NIR absorption spectra were collected from 200-1000 nm on a Shimadzu UV-3600 Plus spectrophotometer at room temperature. NMR spectra were recorded on a Bruker Avance III HD 600 MHz spectrometer in deuterated benzene (C_6D_6) at room temperature. NMR spectra were referenced to the residual proton signals of C_6D_6. Assignment of resonances in the ^1H and $^{13}C\{^1H\}$ NMR spectra were made based on two-dimensional ^1H-^{13}C HSQC NMR spectra. ^{89}Y INEPT NMR spectra were obtained at 29.41 MHz and were externally referenced to 1 M YCl_3 in D_2O. The spectra of $[(Cp^{iPr5})Y(BH_4)_2(THF)]$ and $\mathbf{1_Y}$ were acquired with a five second relaxation delay, with accumulation times of about four hours and one hour, respectively.

Synthesis of $[(Cp^{iPr5})_2Dy_2(\mu\text{-}\eta^6\text{:}\eta^6\text{-}C_6H_6)]$ ($\mathbf{1_{Dy}}$) from $[(Cp^{iPr5})Dy(Cp^*)BH_4]$ (method 1)

Benzene (20 mL) was added to an ampoule containing $[(Cp^{iPr5})Dy(Cp^*)BH_4]$ (590 mg, 1.0 mmol), KC_8 (1.34 g, 10.0 mmol) and a glass-coated stirrer bar. The reaction mixture was stirred at ambient temperature for 10 days, resulting in a dark brown solution and a black precipitate. The solvent was removed under vacuum, and the residue was extracted into *n*-hexane (3 × 15 mL). The combined

hexane extracts were evaporated to dryness *in vacuo*, giving a dark red powder. Crystals of [(Cp^{iPr5})$_2$Dy$_2$(μ-η^6:η^6-C$_6$H$_6$)] (9 mg, about 2% yield) were obtained by storing a concentrated hexane solution at –35 °C for three days.

Synthesis of [(Cp^{iPr5})$_2$Dy$_2$(μ-η^6:η^6-C$_6$H$_6$)] (1$_{Dy}$) from [(Cp^{iPr5})Dy(BH$_4$)$_2$(THF)] (method 2)
Benzene (20 ml), [(Cp^{iPr5})Dy(BH$_4$)$_2$(THF)] (532 mg, 1.0 mmol) and KC$_8$ (670 mg, 5.0 mmol) were added to an ampoule and the resulting suspension was stirred at ambient temperature for three days, during which time a dark brown colour developed. The volatiles were removed under vacuum, and the residue was extracted into toluene (3 × 15 mL). After filtering, the filtrate was concentrated to ca. 15 mL under reduced pressure. Dark red crystals of [(Cp^{iPr5})$_2$Dy$_2$(μ-η^6:η^6-C$_6$H$_6$)] (157 mg, 33% yield) suitable for X-ray crystallography formed by storing the solution at –35 °C for two days. [(Cp^{iPr5})$_2$Dy$_2$(μ-η^6:η^6-C$_6$H$_6$)] does not show any signs of decomposition in the solid-state following storage under argon atmosphere at room temperature for several months. ***Elemental analysis*** [%] calculated for [C$_{46}$H$_{76}$Dy$_2$] (954.06 g/mol): C 57.91, H 8.03; found: C 57.84, H 7.97. ***FTIR*** (cm^{-1}): 2973m, 2934m, 2864m, 1453m, 1380m, 1363m, 1328w, 1312m, 1225w, 1160m, 1111m, 1084m, 909w, 872s, 692s, 546w, 515w, 482s. 443w, 413w.

Synthesis of [(Cp^{iPr5})Y(BH$_4$)$_2$(THF)]
Toluene (25 mL) was added to an ampoule containing NaCp^{iPr5} (500 mg, 2.0 mmol), Y(BH$_4$)$_3$(THF)$_3$ (940 mg, 2.0 mmol) and a glass-coated stirrer bar. The resulting suspension was stirred at 60 °C for 24 h. The solvent was removed under vacuum, and the residue was extracted into *n*-hexane (2 × 30 mL) and filtered. The combined hexane extracts were concentrated to ca. 10 mL, giving colourless microcrystals. Crystals of [(Cp^{iPr5})Y(BH$_4$)$_2$(THF)] suitable for X-ray crystallography were grown from a THF/hexane solution at –35 °C. Yield: 523 mg, 56%. ***Elemental analysis*** [%] calculated for [C$_{48}$H$_{102}$B$_4$O$_2$La$_2$] (1032.35 g/mol): C 61.83, H 11.03; found: C 61.49, H 10.87. ***FTIR*** (cm^{-1}): 2972s, 2929m, 2872m, 2459s, 2357w, 2305w, 2260w, 2197m, 2131s, 1458s, 1382m, 1365s, 1318w, 1248m, 1172s, 1098s, 1084s, 1041w, 1004s, 909w, 856s, 729w, 710w, 669w, 546m, 526m, 484s, 456w. ***^{1}H NMR*** (600.13 MHz, 298 K, C$_6$D$_6$): δ 3.58 (s, 4H, THF), 3.38 (m, *J* = 7.3 Hz, 5H, *i*Pr CH), 1.58 (d, *J* = 6.6 Hz, 15H, *i*Pr CH$_3$), 1.39 (d, *J* = 7.1 Hz, 3H, BH$_4$), 1.25 (d, *J* = 9.4 Hz, 15H, *i*Pr CH$_3$), 1.13 (d, *J* = 6.7 Hz, 2H, BH$_4$), 1.06 (s, 4H, THF), 0.89 (t, *J* = 7.1 Hz, 3H, BH$_4$). ***^{11}B{^{1}H} NMR*** (192.55 MHz, 298 K, C$_6$D$_6$): δ -20.91. ***^{89}Y NMR*** (29.41 MHz, 298 K, C$_6$D$_6$): δ 78.82.

Synthesis of [(Cp^{iPr5})La(BH$_4$)$_2$(THF)]
Following the procedure described for [(Cp^{iPr5})Y(BH$_4$)$_2$(THF)] using toluene (25 mL), NaCp^{iPr5} (500 mg, 2.0 mmol), and La(BH$_4$)$_3$(THF)$_3$ (840 mg, 2.0 mmol), crystals of [(Cp^{iPr5})La(BH$_4$)$_2$(THF)] were isolated. Yield: 510 mg, 49 %. ***Elemental analysis*** [%] calculated for [C$_{48}$H$_{102}$B$_4$O$_2$La$_2$] (1032.35 g/mol): C 55.84, H 9.96; found: C 55.78, H 9.90. ***FTIR*** (cm^{-1}):2968s, 2934m, 2870m, 2439s, 2332w, 2273w, 2234w, 2188m, 2123s, 1460s, 1382m, 1365s, 1314w, 1226w, 1158s, 1094s, 1084s, 1039w, 1008s, 955w, 916w, 858s, 784w, 749w, 710w, 667w, 542w, 521m, 484s, 458w, 433w. ***^{1}H NMR*** (600.13 MHz, 298 K, C$_6$D$_6$): δ 3.53 (s, 4H, THF), 3.35 (m, *J* = 7.3 Hz, 5H, *i*Pr CH), 2.00 (br., 2H, BH$_4$), 1.87 (br., 3H, BH$_4$), 1.71 (br., 3H, BH$_4$), 1.60 (d, *J* = 7.5 Hz, 15H, *i*Pr CH$_3$), 1.23 (d, *J* = 7.4 Hz, 15H, *i*Pr CH$_3$), 1.11 (s, 4H, THF). ***^{11}B{^{1}H} NMR*** (192.55 MHz, 298 K, C$_6$D$_6$): -16.56.

Synthesis of [(Cp^{iPr5})Sm(BH$_4$)$_2$(THF)]
Following the procedure described for [(Cp^{iPr5})Y(BH$_4$)$_2$(THF)], Sm(BH$_4$)$_3$(THF)$_3$ (862 mg, 2.0 mmol) and NaCp^{iPr5} (500 mg, 2.0 mmol) were combined to give [(Cp^{iPr5})Sm(BH$_4$)$_2$(THF)] as orange crystals (489 mg, 46 %). ***Elemental analysis*** [%] calculated for [C$_{48}$H$_{102}$B$_4$O$_2$Sm$_2$] (1055.23 g/mol): C 54.63, H 9.74; found: C 54.42, H 9.90. ***FTIR*** (cm^{-1}): 2970s, 2931m, 2870m, 2453s, 2348w, 2295w, 2246w, 2186m, 2129s, 1460m, 1382m, 1365s, 1318w, 1236w, 1168s, 1098s, 1084s, 1041w, 1006s, 955w, 918w, 858s, 749w, 735w, 710w, 667w, 544w, 523m, 484s, 454w, 431w. ***^{1}H NMR*** (600.13 MHz, 298 K, C$_6$D$_6$): δ 3.91 (s, 5H, *i*Pr CH), 2.17 (d, *J* = 210.5 Hz, 15H, *i*Pr CH$_3$), 1.40 – 0.75 (m, 8H, THF), -1.73 (s, 15H, *i*Pr CH$_3$), -12.92 (br., 8H, BH$_4$). ***^{11}B{^{1}H} NMR*** (192.55 MHz, 298 K, C$_6$D$_6$): δ -35.18.

Synthesis of [(Cp^{iPr5})Gd(BH$_4$)$_2$(THF)]
Following the procedure described for [(Cp^{iPr5})La(BH$_4$)$_2$(THF)], Gd(BH$_4$)$_3$(THF)$_3$ (876 mg, 2.0 mmol) and NaCp^{iPr5} (500 mg, 2.0 mmol) were combined to give [(Cp^{iPr5})Gd(BH$_4$)$_2$(THF)] as colourless crystals (690 mg, 65 %). ***Elemental analysis*** [%] calculated for [C$_{48}$H$_{102}$B$_4$O$_2$Gd$_2$] (1069.03 g/mol):

C 53.93, H 9.62; found: C 53.62, H 9.54. ***FTIR*** (cm^{-1}): 2966s, 2931m, 2872m, 2468s, 2369w, 2312w, 2260w, 2199m, 2135s, 1460s, 1380m, 1365s, 1314w, 1252m, 1176s, 1106s, 1039w, 1004s, 955w, 920w, 856s, 799s, 749w, 714w, 688w, 671w, 618w, 585w, 546w, 521s, 470m, 415m.

Synthesis of [(Cp^{iPr5})$_2$Y$_2$(μ-η^6:η^6-C$_6$H$_6$)] (1$_Y$)

Following method 2 described for **1$_{Dy}$**, [(Cp^{iPr5})Y(BH$_4$)$_2$(THF)] (459 mg, 1.0 mmol) and KC_8 (670 mg, 5.0 mmol) were combined to give [(Cp^{iPr5})$_2$Y$_2$(μ-η^6:η^6-C$_6$H$_6$)] as dark red crystals (128 mg, 32 %). ***Elemental analysis*** [%] calculated for [$C_{46}H_{76}Y_2$] (808.88 g/mol): C 68.47, H 9.49; found: C 68.48, H 9.43. ***FTIR*** (cm^{-1}): 2973m, 2936m, 2864m, 1453m, 1380m, 1363m, 1328w, 1312m, 1226w, 1160m, 1111m, 1084s, 909w, 872s, 692s, 546w, 515w, 482s, 445w 441w. ***^{1}H NMR*** (600.13 MHz, 298 K, C_6D_6): δ 3.75 (s, 6H, C_6H_6), 3.25 (m, *J* = 7.3 Hz, 10H, *i*Pr CH), 1.59 (d, *J* = 7.3 Hz, 30H, *i*Pr CH_3), 1.29 (d, *J* = 7.2 Hz, 30H, *i*Pr CH_3). ***^{13}C{^{1}H} NMR*** (150.92 MHz, 298 K, C_6D_6): 126.16 (Cp ring C_5), 59.17(μ-η^6:η^6-C$_6$H$_6$), 27.04 (*i*Pr CH), 25.44 (*i*Pr CH_3), 23.48 (*i*Pr CH_3). ***^{89}Y NMR*** (29.41 MHz, 298 K, C_6D_6): δ -102.93.

Synthesis of [(Cp^{iPr5})$_2$La$_2$(μ-η^6:η^6-C$_6$H$_6$)]·3benzene (1$_{La}$)

Following method 2 described for **1$_{Dy}$**, [(Cp^{iPr5})La(BH$_4$)$_2$(THF)] (516 mg, 1.0 mmol) and KC_8 (670 mg, 5.0 mmol) were combined to give [(Cp^{iPr5})$_2$La$_2$(μ-η^6:η^6-C$_6$H$_6$)] as dark red powder. ***Elemental analysis*** [%] calculated for [$C_{46}H_{76}La_2$] (906.88 g/mol): C 60.92, H 8.45; found: C 60.78, H 8.54. Recrystallizing the powder in hot benzene gives [(Cp^{iPr5})$_2$La$_2$(μ-η^6:η^6-C$_6$H$_6$)]·3Benzene (101 mg, 22 %) suitable for X-ray crystallography. ***FTIR*** (cm^{-1}): 2970s, 2934m, 2866m, 1455m, 1377m, 1363m, 1343w, 1310w, 1265w, 1158m, 1109w, 1082m, 905w, 877s, 731w, 671s, 546w, 511w, 482s, 441w. ***^{1}H NMR*** (600.13 MHz, 298 K, C_6D_6): δ 3.79 (s, 6H, C_6H_6), 3.25 (m, *J* = 7.3 Hz, 10H, *i*Pr CH), 1.60 (d, *J* = 7.4 Hz, 30H, *i*Pr CH_3), 1.31 (d, *J* = 7.3 Hz, 30H, *i*Pr CH_3). ***^{13}C{^{1}H} NMR*** (150.92 MHz, 298 K, C_6D_6): 126.88 (Cp ring C_5), 67.17 (μ-η^6:η^6-C$_6$H$_6$), 26.92 (*i*Pr CH), 23.72 (*i*Pr CH_3), 23.26 (*i*Pr CH_3).

Synthesis of [(Cp^{iPr5})$_2$Sm$_2$(μ-η^6:η^6-C$_6$H$_6$)] (1$_{Sm}$)

Following method 2 described for **1$_{Dy}$**, [(Cp^{iPr5})Sm(BH$_4$)$_2$(THF)] (527 mg, 1.0 mmol) and KC_8 (670 mg, 5.0 mmol) were combined to give [(Cp^{iPr5})$_2$Sm$_2$(μ-η^6:η^6-C$_6$H$_6$)] as dark red crystals (146 mg, 31 %). ***Elemental analysis*** [%] calculated for [$C_{46}H_{76}Sm_2$] (943.56 g/mol): C 59.42, H 8.24; found: C 59.18, H 8.31. ***FTIR*** (cm^{-1}): 2973s, 2931s, 2866s, 1455s, 1380m, 1363s, 1343w, 1312m, 1226w, 1158m, 1111m, 1082s, 909w, 879s, 751w, 682s, 546w, 513w, 482s, 439w, 415w. ***^{1}H NMR*** (600.13 MHz, 298 K, C_6D_6): δ 21.13 (s, 6H, C_6H_6), 4.45 - 4.37 (m, 10H, *i*Pr CH), 2.74 (d, *J* = 7.1 Hz, 30H, *i*Pr CH_3), -2.01 (d, *J* = 7.1 Hz, 30H, *i*Pr CH_3). ***^{13}C{^{1}H} NMR*** (150.92 MHz, 298 K, C_6D_6): 132.02 (Cp ring C_5), 35.64 (*i*Pr CH), 22.84 (*i*Pr CH_3), 19.38 (*i*Pr CH_3), 3.07(μ-η^6:η^6-C$_6$H$_6$).

Synthesis of [(Cp^{iPr5})$_2$Gd$_2$(μ-η^6:η^6-C$_6$H$_6$)] (1$_{Gd}$)

Following method 2 described for **1$_{Dy}$**, [(Cp^{iPr5})Gd(BH$_4$)$_2$(THF)] (527 mg, 1.0 mmol) and KC_8 (670 mg, 5.0 mmol) were combined to give [(Cp^{iPr5})$_2$Gd$_2$(μ-η^6:η^6-C$_6$H$_6$)] as dark red crystals (150 mg, 32 %). ***Elemental analysis*** [%] calculated for [$C_{46}H_{76}Gd_2$] (943.56 g/mol): C 58.55, H 8.12; found: C 58.61, H 8.29. ***FTIR*** (cm^{-1}): 2973m, 2936m, 2864m, 1453m, 1380m, 1363m, 1328w, 1312m, 1225w, 1160m, 1111m, 1084m, 909w, 870s, 696s, 546w, 515w, 484s, 441w, 415w.

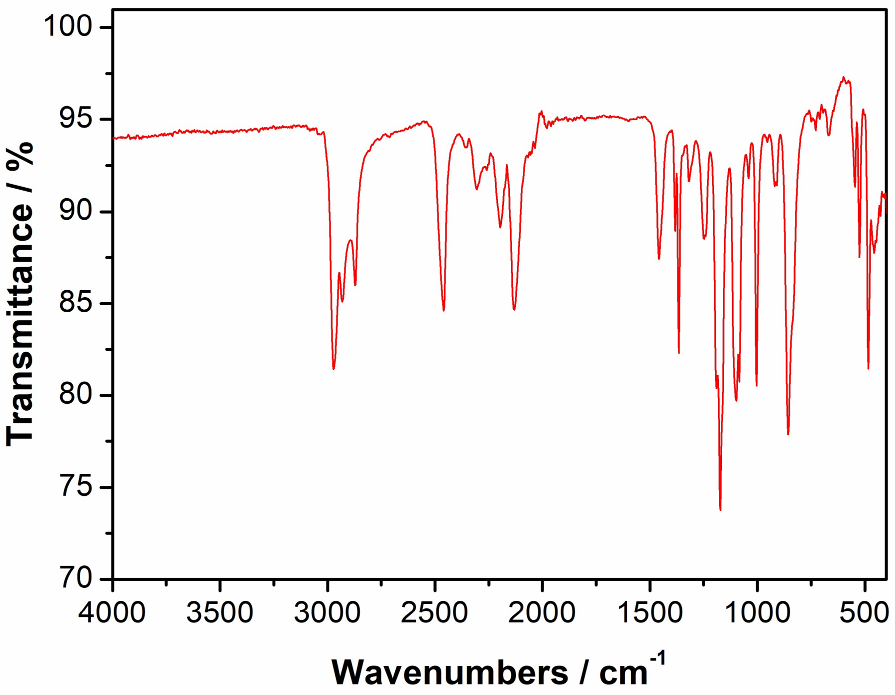

Figure S1. FTIR spectrum of [(Cp^{iPr5})Y(BH_4)$_2$(THF)].

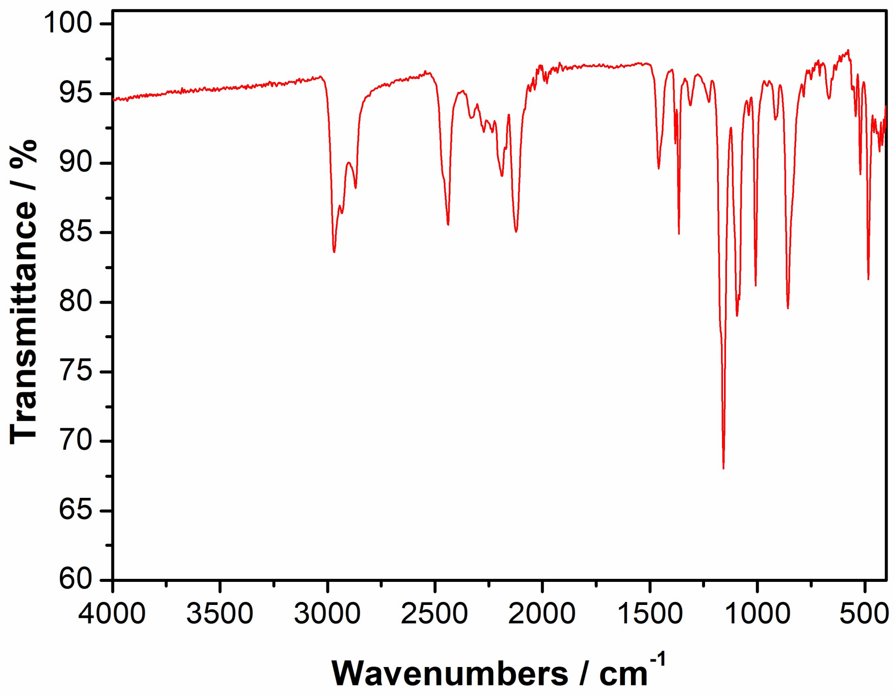

Figure S2. FTIR spectrum of [(Cp^{iPr5})La(BH_4)$_2$(THF)].

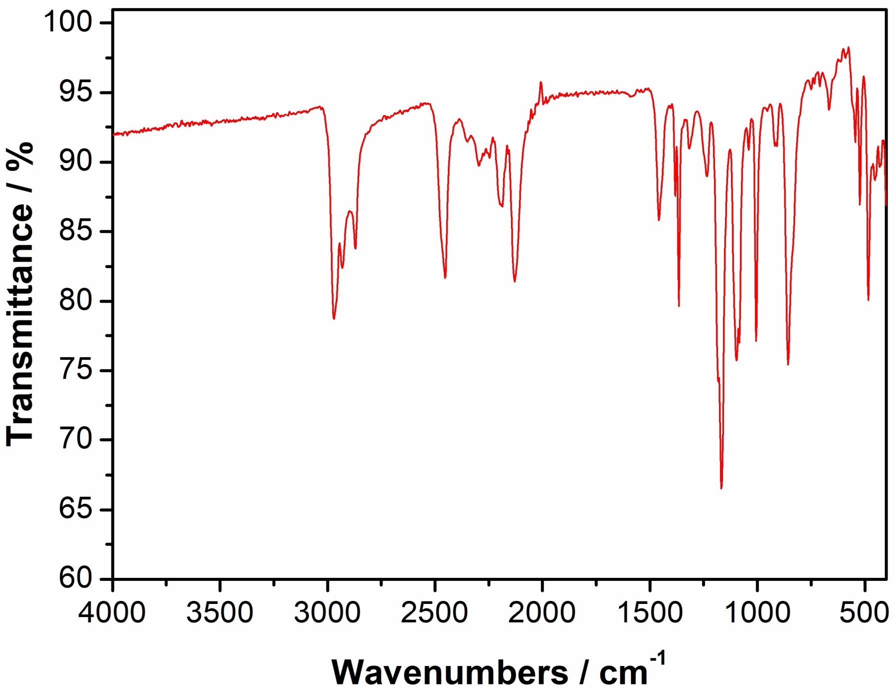

Figure S3. FTIR spectrum of [(Cp^{iPr5})Sm(BH_4)$_2$(THF)].

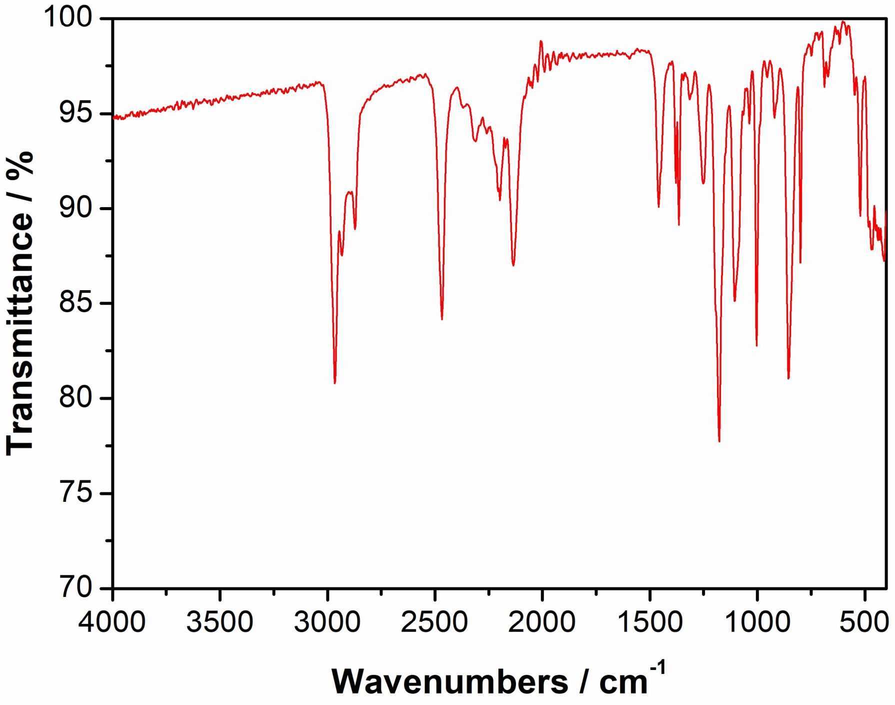

Figure S4. FTIR spectrum of [(Cp^{iPr5})Gd(BH_4)$_2$(THF)].

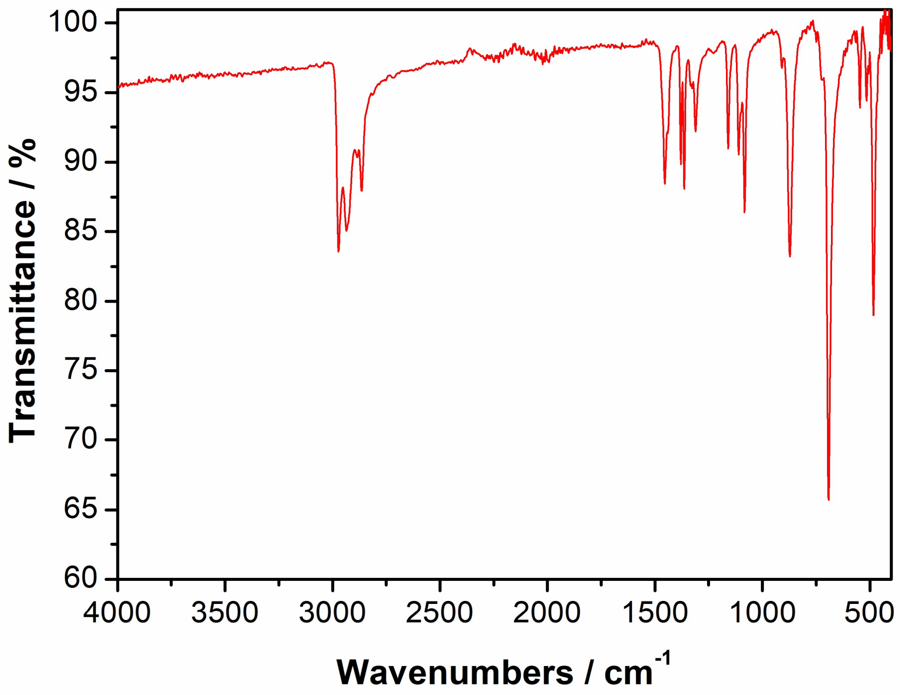

Figure S5. FTIR spectrum of $\mathbf{1}_{Y}$.

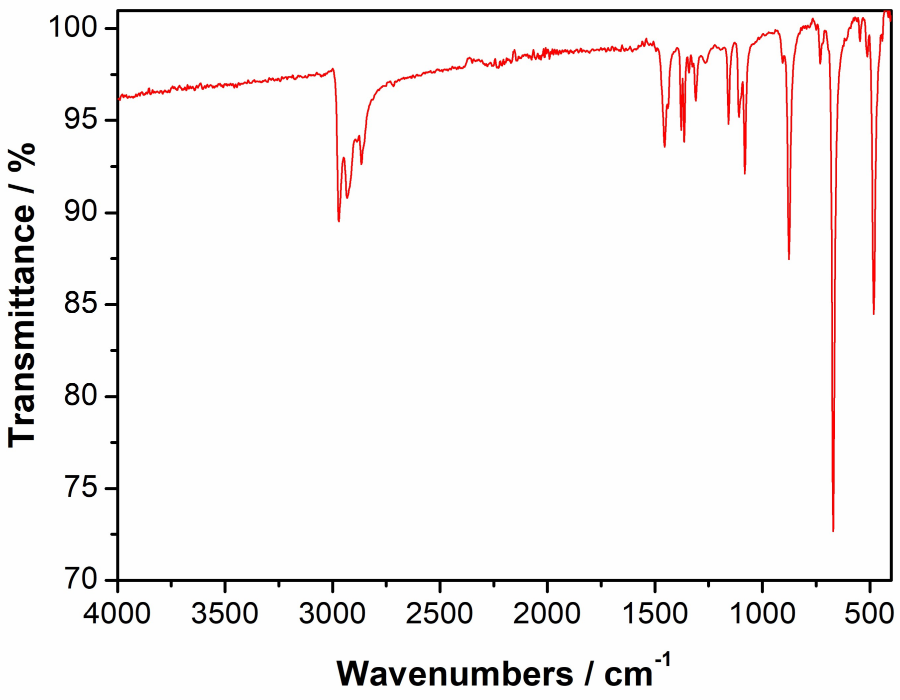

Figure S6. FTIR spectrum of $\mathbf{1}_{La}$.

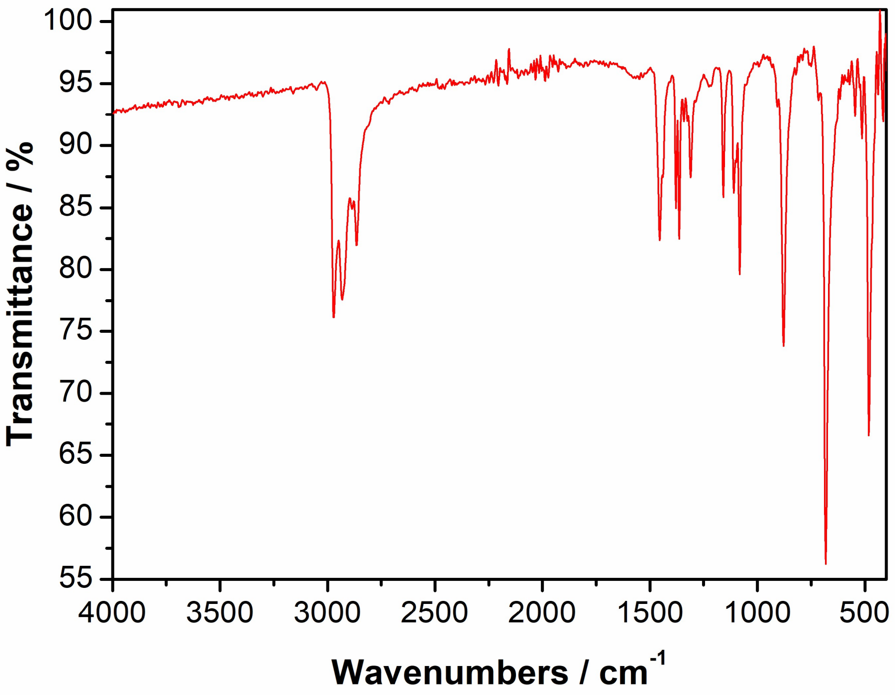

Figure S7. FTIR spectrum of $\mathbf{1}_{Sm}$.

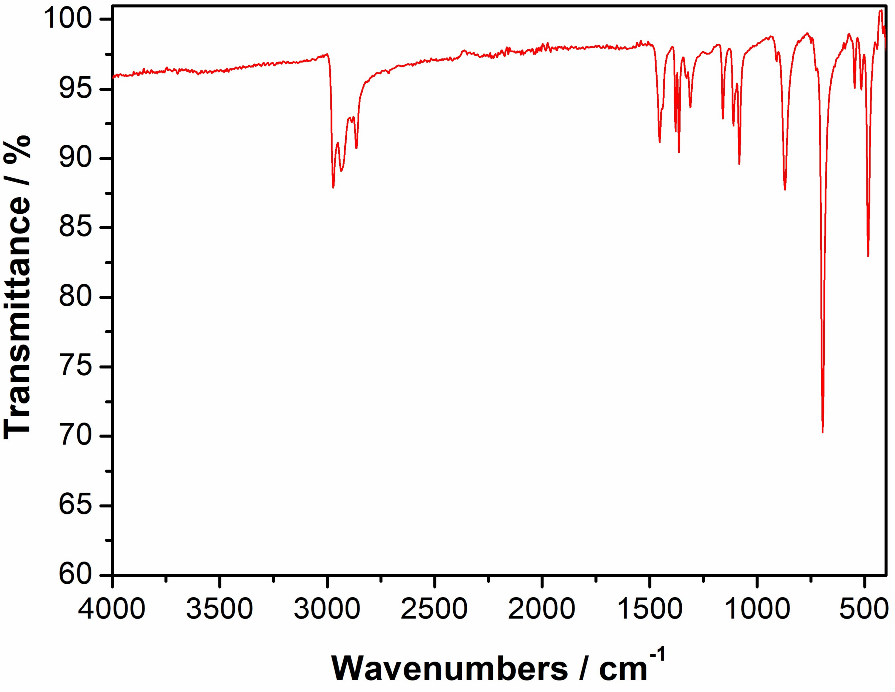

Figure S8. FTIR spectrum of $\mathbf{1}_{Gd}$.

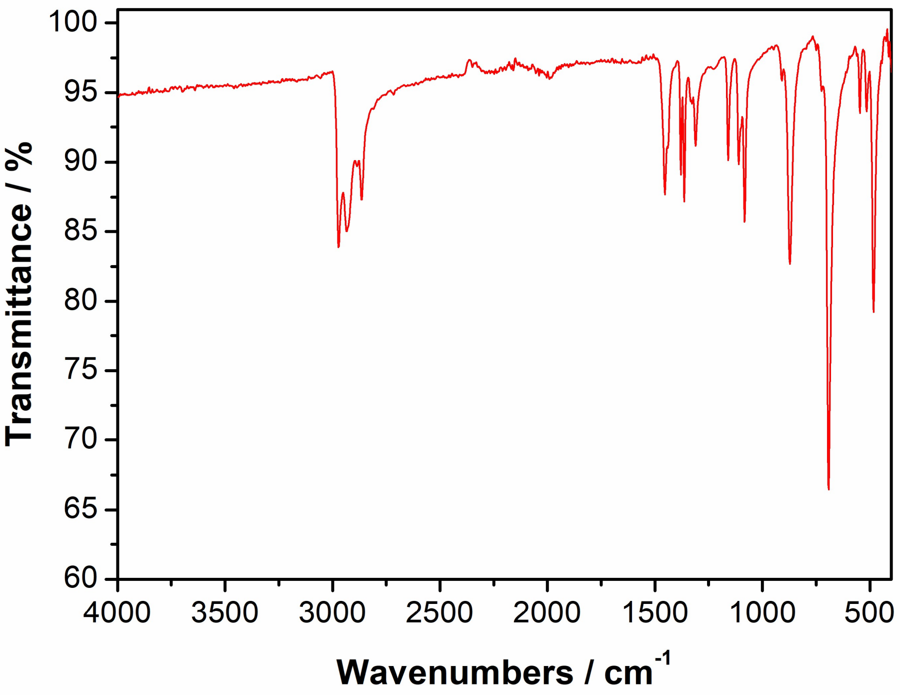

Figure S9. FTIR spectrum of $\mathbf{1}_{\mathbf{Dy}}$.

X-ray crystallography

X-ray diffraction data were collected for $[(Cp^{iPr5})M(BH_4)_2(THF)$ (M = Y, La, Gd) and $[(Cp^{iPr5})_2M_2(\mu\text{-}\eta^6{:}\eta^6\text{-}C_6H_6)]$ (M = La) on Bruker APEX-II CCD diffractometer. Data collection and processing (cell refinement, data reduction and absorption) were performed using the program *APEX 3*. X-ray diffraction data were collected on a Rigaku XtaLAB Synergy-R HyPix diffractometer for $[(Cp^{iPr5})M(BH_4)_2(THF)$ (M = Sm)and $[(Cp^{iPr5})_2M_2(\mu\text{-}\eta^6{:}\eta^6\text{-}C_6H_6)]$ (M = Y, Sm, Dy, Gd). Data collection and processing (cell refinement, data reduction and absorption) of the three complexes were performed using the program *CrysAlisPro*. Structures were solved in Olex2 with SHELXT using intrinsic phasing and were refined with SHELXL using least squares minimisation.[3–5] Anisotropic thermal parameters were used for the non-hydrogen atoms and isotropic parameters for the hydrogen atoms. Hydrogen atoms were added geometrically and refined using a riding model.

1_{Y}. The Cp^{iPr5} ligand in the structure is disordered and was refined assuming all the isopropyl groups disorder over two opposite orientations, yielding a ratio of 0.718:0.282 for the two components. The C_6H_6 ring shows disorder over the symmetry element (inversion center), and the occupancies of these two components were refined corresponding to the multiplicity (0.5) of the symmetry operator. The distances (C6A-C1, C6-C1, C9-C2, C9A-C2, C5-C18, C5-C18A, C4-C15, C4-C15A, C3-C12A, C3-C12) between the *i*Pr groups and the Cp ring were fixed to be 1.54 Å using DFIX restraints. Three carbons (C23, C24 and C26, non-positive definite) in the C_6H_6 ring could not be stabilized unless anisotropic displacement parameters were forced to behave in isotropic manner. No geometrical restraints on bond lengths and angles were applied to the refinement of the C_6H_6 plane.

1_{La}. One Cp^{iPr5} ligand (coordinates to La2) in the structure is clearly disordered and was refined assuming all the isopropyl groups disorder over two opposite orientations, yielding a ratio of 0.620:0.380 for the two components. The distances (C29-C22, C29A-C22, C32A-C23 C32-C23) between the *i*Pr groups and the Cp ring were fixed to be 1.54 Å using DFIX restraints. No geometrical restraints on bond lengths and angles were applied to the refinement of the C_6H_6 plane.

1_{Sm}. The Cp^{iPr5} ligand in the structure is disordered and was refined assuming all the isopropyl groups disorder over two opposite orientations, yielding a ratio of 0.670:0.330 for the two components. The C_6H_6 ring shows disorder over the symmetry element (inversion center), and the occupancies of these two components were refined corresponding to the multiplicity (0.5) of the symmetry operator. The distances (C6A-C1, C6-C1, C3-C12A, C3-C12, C5-C18, C5-C18A, C4-C15, C4-C15A, C2-C9A, C2-C9) between the *i*Pr groups and the Cp ring were fixed to be 1.54 Å using DFIX restraints. Three carbons (C21, C24 and C25, non-positive definite) in the C_6H_6 ring could not be stabilized unless anisotropic displacement parameters were forced to behave in isotropic manner. No geometrical restraints on bond lengths and angles were applied to the refinement of the C_6H_6 plane.

1_{Gd}. The Cp^{iPr5} ligand in the structure is disordered and was refined assuming all the isopropyl groups disorder over two opposite orientations, yielding a ratio of 0.694:0.306 for the two components. The C_6H_6 ring shows disorder over the symmetry element (inversion center), and the occupancies of these two components were refined corresponding to the multiplicity (0.5) of the symmetry operator. The distances (C6A-C1, C6-C1, C9-C2, C9A-C2, C5-C18, C5-C18A, C4-C15, C4-C15A, C3-C12A, C3-C12) between the *i*Pr groups and the Cp ring were fixed to be 1.54 Å using DFIX restraints. One carbon (C25, non-positive definite) in the C_6H_6 ring could not be stabilized unless anisotropic displacement parameters were forced to behave in am isotropic manner. No geometrical restraints on bond lengths and angles were applied to the refinement of the C_6H_6 plane.

1_{Dy}. The Cp^{iPr5} ligand in the structure is disordered and was refined assuming all the isopropyl groups disorder over two opposite orientations, yielding a ratio of 0.705:0.295 for the two components. The C_6H_6 ring shows disorder over the symmetry element (inversion center), and the occupancies of these two components were refined corresponding to the multiplicity (0.5) of the symmetry operator. Three carbons (C21, C23 and C25, non-positive definite) in the C_6H_6 ring could not be stabilized unless anisotropic displacement parameters were forced to behave in an isotropic manner. No geometrical restraints on bond lengths and angles were applied to the refinement of the C_6H_6 plane.

Table S1. Crystal data and structure refinement for [(Cp^{iPr5})M(BH_4)$_2$(THF)].

**Complex**	M = Y	M = La	M = Sm	M = Gd
CCDC	2416583	2416584	2416585	2416586
Formula	$C_{48}H_{102}B_4Y_2O_2$	$C_{48}H_{102}B_4La_2O_2$	$C_{48}H_{102}B_4Sm_2O_2$	$C_{48}H_{102}B_4Gd_2O_2$
FW	932.35	1032.35	1055.23	1069.03
Crystal system	Monoclinic	Monoclinic	Monoclinic	Monoclinic
Space group	$P2_1/c$	$P2_1/c$	$P2_1/c$	$P2_1/c$
a/Å	9.9289(3)	9.8836(6)	9.92431(11)	9.9098(4)
b/Å	33.7135(11)	34.5443(19)	34.1046(4)	33.9353(12)
c/Å	16.2591(5)	16.2328(10)	16.26132(18)	16.2429(5)
α/°	90	90	90	90
β/°	94.0940(10)	94.418(2)	94.1784(10)	94.1870(10)
γ/°	90	90	90	90
V/Å^3	5428.6(3)	5525.8(6)	5489.25(11)	5447.8(3)
Temperature/K	100.0	100.0	100.0	100.0
Z	4	4	4	4
ρ_{calc}/g cm^{-3}	1.141	1.241	1.277	1.303
Crystal size/mm^3	0.08×0.1×0.2	0.03×0.06×0.09	0.03×0.03×0.05	0.1×0.12×0.15
Radiation	Mo $K\alpha$ (λ = 0.71073)	Mo $K\alpha$ (λ = 0.71073)	Cu $K\alpha$ (λ = 1.54184)	Mo $K\alpha$ (λ = 0.71073)
2θ range/°	4.122 to 55.802	4.134 to 55.836	6.034 to 153.64	4.122 to 55.802
Reflections collected	153993	122158	39259	77110
Independent reflections	13495 [R_{int}=0.0667, R_{sigma}=0.0327]	13182 [R_{int}=0.0518, R_{sigma}=0.0266]	11107 [R_{int}=0.0503, R_{sigma}=0.0457]	13005 [R_{int}=0.0479, R_{sigma}=0.0323]
Completeness/%	100.0	100.0	99.8	100.0
Data/restraints/parameters	13495/35/617	13182/10/571	11107/0/525	13005/38/617
Goodness-of-fit on F^2	1.036	1.246	1.042	1.184
Final R indices [I>2σ(I)]	R_1= 0.0355 wR_2 = 0.0832	R_1= 0.0453 wR_2 = 0.0859	R_1= 0.0441 wR_2 = 0.1120	R_1= 0.0354 wR_2 = 0.0638
Final R indices (all data)	R_1 = 0.0499 wR_2 = 0.0892	R_1 = 0.0489 wR_2 = 0.0873	R_1 = 0.0505 wR_2 = 0.1152	R_1 = 0.0400 wR_2 = 0.0653

Table S2. Crystal data and structure refinement for $\mathbf{1_M}$.

**Complex**	$\mathbf{1_Y}$	$\mathbf{1_{La}}$	$\mathbf{1_{Sm}}$	$\mathbf{1_{Gd}}$	$\mathbf{1_{Dy}}$
CCDC	2416587	2416588	2416589	2416590	2416591
Formula	$C_{46}H_{76}Y_2$	$C_{64}H_{94}La_2$	$C_{46}H_{76}Sm_2$	$C_{46}H_{76}Gd_2$	$C_{46}H_{76}Dy_2$
FW	806.88	1141.21	929.76	943.56	954.06
Crystal system	Monoclinic	Triclinic	Monoclinic	Monoclinic	Monoclinic
Space group	*P*2/*n*	*P*-1	*P*2/*n*	*P*2/*n*	*P*2/*n*
a/Å	15.3808(5)	10.2748(5)	15.5935(4)	15.5122(2)	15.3876(2)
b/Å	9.8370(3)	17.5147(9)	9.7958(2)	9.81375(1)	9.83140(10)
c/Å	16.1980(5)	17.7960(8)	16.2243(5)	16.2046(3)	16.1993(2)
α/°	90	69.892(2)	90	90	90
β/°	116.389(4)	77.449(2)	117.017(4)	116.741(2)	116.432(2)
γ/°	90	77.574(2)	90	90	90
V/Å^3	2195.41(13)	2900.8(2)	2207.79(12)	2203.05(7)	2194.47(6)
Temperature/K	100.0	100.0	100.0	100.0	100.0
Z	2	2	2	2	2
ρ_{calc}/g cm^{-3}	1.221	1.307	1.399	1.422	1.444
Crystal size/mm^3	0.05×0.05×0.05	0.08×0.1×0.12	0.05×0.05×0.05	0.03×0.04×0.05	0.04×0.05×0.05
Radiation	Cu $K\alpha$ (λ = 1.54184)	Mo $K\alpha$ (λ = 0.71073)	Cu $K\alpha$ (λ = 1.54184)	Cu $K\alpha$ (λ = 1.54184)	Cu $K\alpha$ (λ = 1.54184)
2θ range/°	6.598 to 153.714	4.028 to 56.672	6.522 to 151.92	6.554 to 152.886	6.594 to 153.514
Reflections collected	15095	94372	14664	15512	24878
Independent reflections	4427 [R_{int}=0.0668, R_{sigma}=0.0527]	14402 [R_{int} = 0.0398, R_{sigma} = 0.0237]	4364 [R_{int}=0.0528, R_{sigma}=0.0512]	4454 [R_{int}=0.0314, R_{sigma}=0.0289]	4474 [R_{int}=0.0321, R_{sigma}=0.0181]
Completeness/%	99.7	99.3	98.2	99.8	99.9
Data/restraints/parameters	4427/28/300	14402/4/661	4364/28/300	4454/16/300	4474/28/300
Goodness-of-fit on F^2	1.122	1.063	1.042	1.034	1.091
Final *R* indices [I>2σ(I)]	R_1 = 0.0582 wR_2 = 1516	R_1= 0.0293 wR_2 = 0.0661	R_1= 0.0419 wR_2 = 0.1126	R_1= 0.0252 wR_2 = 0.0595	R_1= 0.0256 wR_2 = 0.667
Final *R* indices (all data)	R_1 = 0.0689 wR_2 = 0.1544	R_1 = 0.0344 wR_2 = 0.0691	R_1 = 0.0504 wR_2 = 0.1179	R_1 = 0.0281 wR_2 = 0.0606	R_1 = 0.0288 wR_2 = 0.0689

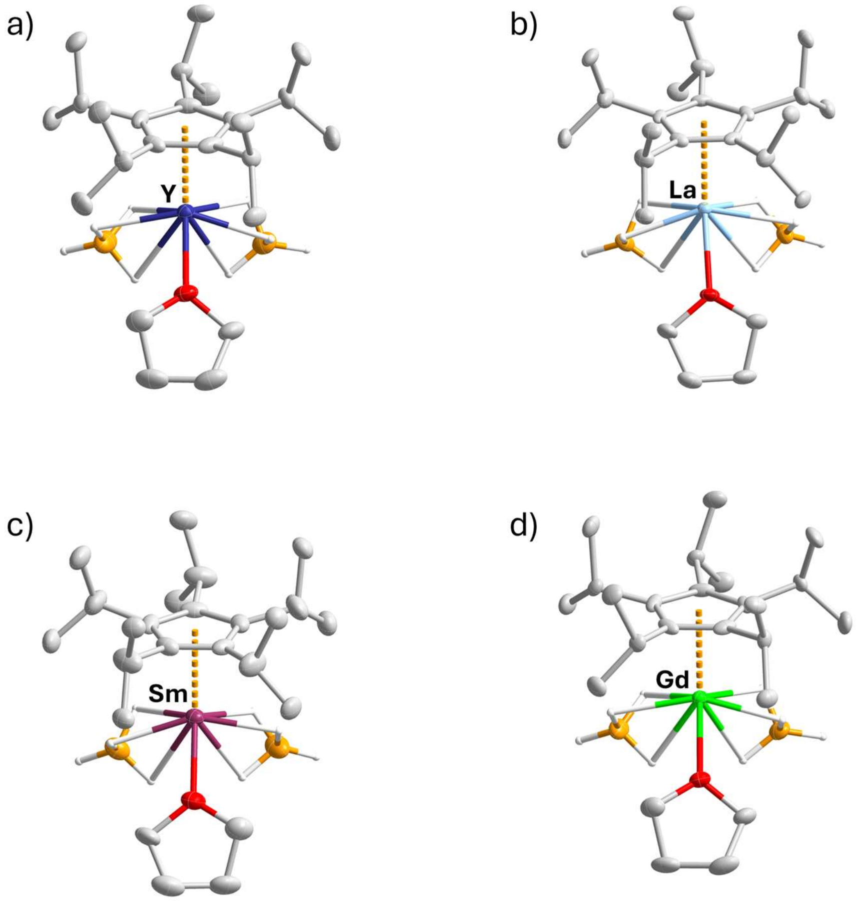

Figure S10. Thermal ellipsoid representation (50% probability) of the molecular structures of [(Cp^{iPr5})M(BH$_4$)$_2$(THF)] with: a) M = Y, b) M = La, c) M = Sm, d) M = Gd. Unlabeled atoms are carbon (grey) and hydrogen (white).

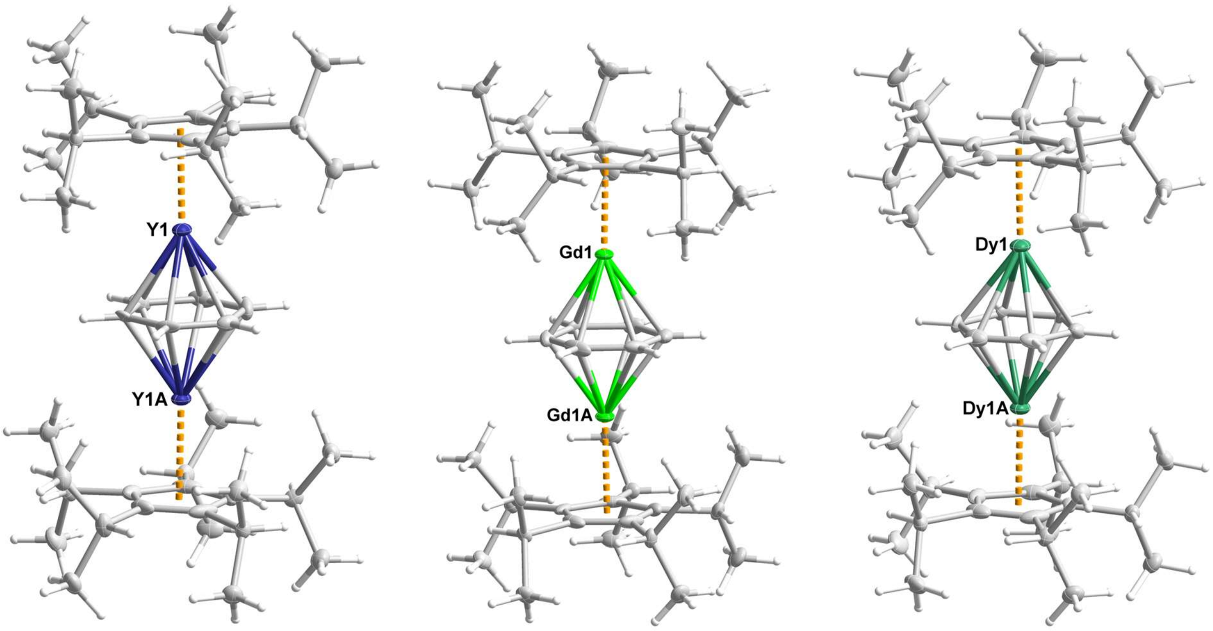

Figure S11. Thermal ellipsoid representation (50% probability) of the molecular structures of $\mathbf{1}_{\mathrm{Y}}$, $\mathbf{1}_{\mathrm{Gd}}$ and $\mathbf{1}_{\mathrm{Dy}}$. Unlabeled atoms are carbon (grey) and hydrogen (white).

Table S3. Selected bond lengths (Å) in [(Cp^{iPr5})Y(BH_4)$_2$(THF)].

Y1-O1	2.3850(15)	Y2-O2	2.3674(15)
Y1-C1	2.6698(19)	Y2-C25	2.668(2)
Y1-C2	2.632(2)	Y2-C26	2.665(2)
Y1-C3	2.639(2)	Y2-C27	2.6341(19)
Y1-C4	2.672(2)	Y2-C28	2.640(2)
Y1-C5	2.6762(19)	Y2-C29	2.662(2)
Y1-B1	2.499(3)	Y2-B3	2.502(3)
Y1-B2	2.502(3)	Y2-B4	2.505(3)
Y1-Cp$_{cent}$	2.3681(3)	Y2-Cp$_{cent}$	2.3653(3)

‡There are two unique molecules in the asymmetric unit of [(Cp^{iPr5})Y(BH_4)$_2$(THF)].

Table S4. Selected bond lengths (Å) in [(Cp^{iPr5})La(BH_4)$_2$(THF)].

La1-O1	2.545(3)	La2-O2	2.538(3)
La1-C1	2.771(4)	La2-C25	2.850(3)
La1-C2	2.835(3)	La2-C26	2.825(3)
La1-C3	2.849(3)	La2-C27	2.771(3)
La1-C4	2.826(4)	La2-C28	2.771(3)
La1-C5	2.781(4)	La2-C29	2.834(3)
La1-B1	2.665(4)	La2-B3	2.693(4)
La1-B2	2.673(4)	La2-B4	2.680(4)
La1-Cp$_{cent}$	2.5424(3)	La2-Cp$_{cent}$	2.5388(3)

‡There are two unique molecules in the asymmetric unit of [(Cp^{iPr5})La(BH_4)$_2$(THF)].

Table S5. Selected bond lengths (Å) in [(Cp^{iPr5})Sm(BH_4)$_2$(THF)].

Sm1-O1	2.465(3)	Sm2-O2	2.455(4)
Sm1-C1	2.690(4)	Sm2-C25	2.741(5)
Sm1-C2	2.737(4)	Sm2-C26	2.715(5)
Sm1-C3	2.751(4)	Sm2-C27	2.699(5)
Sm1-C4	2.730(4)	Sm2-C28	2.689(4)
Sm1-C5	2.695(5)	Sm2-C29	2.732(5)
Sm1-B1	2.553(6)	Sm2-B3	2.568(6)
Sm1-B2	2.577(6)	Sm2-B4	2.569(6)
Sm1-Cp_{cent}	2.4402(3)	Sm2-Cp_{cent}	2.4335(3)

‡There are two unique molecules in the asymmetric unit of [(Cp^{iPr5})Sm(BH_4)$_2$(THF)].

Table S6. Selected bond lengths (Å) in [(Cp^{iPr5})Gd(BH_4)$_2$(THF)].

Gd1-O1	2.431(2)	Gd2-O2	2.418(2)
Gd1-C1	2.675(3)	Gd2-C25	2.673(3)
Gd1-C2	2.711(3)	Gd2-C26	2.704(3)
Gd1-C3	2.717(3)	Gd2-C27	2.713(3)
Gd1-C4	2.711(3)	Gd2-C28	2.710(3)
Gd1-C5	2.664(3)	Gd2-C29	2.668(3)
Gd1-B1	2.544(4)	Gd2-B3	2.545(4)
Gd1-B2	2.532(4)	Gd2-B4	2.548(4)
Gd1-Cp_{cent}	2.40997(9)	Gd2-Cp_{cent}	2.40721(8)

‡There are two unique molecules in the asymmetric unit of [(Cp^{iPr5})Gd(BH_4)$_2$(THF)].

Table S7. Selected bond lengths (Å) and angle (°) in **1_{Y}**.

Y1-C21	2.501(19)	C21-C22	1.46(2)
Y1-C22	2.504(14)	C21-C26	1.44(3)
Y1-C23	2.482(19)	C22-C23	1.44(2)
Y1-C24	2.421(19)	C23-C24	1.47(3)
Y1-C25	2.387(14)	C24-C25	1.42(2)
Y1-C26	2.400(18)	C25-C26	1.47(2)
Y1-C1	2.654(5)	Y1-Cp_{cent}	2.368(3)
Y1-C2	2.657(5)	Y1-$(C_6)_{cent}$	1.974(9)
Y1-C3	2.652(5)	Y1A-$(C_6)_{cent}$	2.000(9)
Y1-C4	2.656(5)	Y1-$(C_6)_{cent}$-Y1A	173.3(4)
Y1-C5	2.658(5)	Cp_{cent}-Y1-$(C_6)_{cent}$	176.5(2)
Y1···Y1A	3.9673(8)	Cp_{cent}A-Y1A-$(C_6)_{cent}$	176.4(2)

Table S8. Selected bond lengths (Å) and angle (°) in **1_{La}**.

La1-C1	2.846(2)	La2-C21	2.845(2)
La1-C2	2.856(2)	La2-C22	2.850(2)
La1-C3	2.851(2)	La2-C23	2.861(2)
La1-C4	2.846(2)	La2-C24	2.855(2)
La1-C5	2.843(2)	La2-C25	2.848(2)
La1-C41	2.607(3)	La2-C41	2.611(2)
La1-C42	2.611(3)	La2-C42	2.605(3)
La1-C43	2.618(3)	La2-C43	2.611(3)
La1-C44	2.620(3)	La2-C44	2.619(3)
La1-C45	2.610(3)	La2-C45	2.617(3)
La1-C46	2.610(2)	La2-C46	2.615(2)
C41-C42	1.451(5)	La1-Cp_{cent}	2.58126(19)
C41-C46	1.448(5)	La2-Cp_{cent}	2.58738(19)
C42-C43	1.432(6)	La1-$(C_6)_{cent}$	2.18609(18)
C43-C44	1.405(6)	La2-$(C_6)_{cent}$	2.18657(18)
C44-C45	1.410(5)	La1-$(C_6)_{cent}$-La2	178.837(7)
C45-C46	1.439(5)	Cp_{cent}-La1-$(C_6)_{cent}$	176.521(9)
La1··· La2	4.3724(3)	Cp_{cent}-La2-$(C_6)_{cent}$	176.665(9)

Table S9. Selected bond lengths (Å) and angle (°) in $\mathbf{1}_{\mathbf{Sm}}$.

Sm1-C21	2.515(14)	C21-C22	1.447(18)
Sm1-C22	2.559(11)	C21-C26	1.44(2)
Sm1-C23	2.578(12)	C22-C23	1.45(2)
Sm1-C24	2.53(3)	C23-C24	1.43(3)
Sm1-C25	2.477(11)	C24-C25	1.47(2)
Sm1-C26	2.474(13)	C25-C26	1.47(2)
Sm1-C1	2.736(15)	Sm1-Cp_{cent}	2.4639(3)
Sm1-C2	2.740(4)	Sm1-$(C_6)_{cent}$	2.0636(3)
Sm1-C3	2.736(5)	Sm1A-$(C_6)_{cent}$	2.0996(3)
Sm1-C4	2.739(5)	Sm1-$(C_6)_{cent}$-Sm1A	174.0374(16)
Sm1-C5	2.752(5)	Cp_{cent}-Sm1-$(C_6)_{cent}$	177.494(15)
Sm1··· Sm1A	4.1576(6)	Cp_{cent}A-Sm1A-$(C_6)_{cent}$	176.142(16)

Table S10. Selected bond lengths (Å) and angle (°) in $\mathbf{1}_{\mathbf{Gd}}$.

Gd1-C21	2.565(7)	C21-C22	1.452(13)
Gd1-C22	2.566(10)	C21-C26	1.449(11)
Gd1-C23	2.51(2)	C22-C23	1.448(16)
Gd1-C24	2.455(8)	C23-C24	1.438(15)
Gd1-C25	2.472(11)	C24-C25	1.465(13)
Gd1-C26	2.523(11)	C25-C26	1.455(14)
Gd1-C1	2.702(2)	Gd1-Cp_{cent}	2.4220(14)
Gd1-C2	2.699(3)	Gd1-$(C_6)_{cent}$	2.055(6)
Gd1-C3	2.701(2)	Gd1A-$(C_6)_{cent}$	2.022(6)
Gd1-C4	2.703(3)	Gd1-$(C_6)_{cent}$-Gd1A	173.4(2)
Gd1-C5	2.709(2)	Cp_{cent}-Gd1-$(C_6)_{cent}$	176.10(12)
Gd1···Gd1A	4.0707(2)	Cp_{cent}A-Gd1A-$(C_6)_{cent}$	176.71(13)

Table S11. Selected bond lengths (Å) and angle (°) in $\mathbf{1}_{\mathbf{Dy}}$.

Dy1-C21	2.501(7)	C21-C22	1.418(12)
Dy1-C22	2.508(7)	C21-C26	1.485(16)
Dy1-C23	2.491(10)	C22-C23	1.459(11)
Dy1-C24	2.398(9)	C23-C24	1.488(13)
Dy1-C25	2.372(7)	C24-C25	1.437(12)
Dy1-C26	2.42(2)	C25-C26	1.455(17)
Dy1-C1	2.669(3)	Dy1-Cp_{cent}	2.3806(15)
Dy1-C2	2.671(3)	Dy1-$(C_6)_{cent}$	1.969(5)
Dy1-C3	2.662(3)	Dy1A-$(C_6)_{cent}$	2.019(6)
Dy1-C4	2.659(3)	Dy1-$(C_6)_{cent}$-Dy1A	172.5(3)
Dy1-C5	2.670(2)	Cp_{cent}-Dy1-$(C_6)_{cent}$	176.10(13)
Dy1···Dy1A	3.9798(3)	Cp_{cent}A-Dy1A-$(C_6)_{cent}$	176.19(12)

NMR Spectroscopy

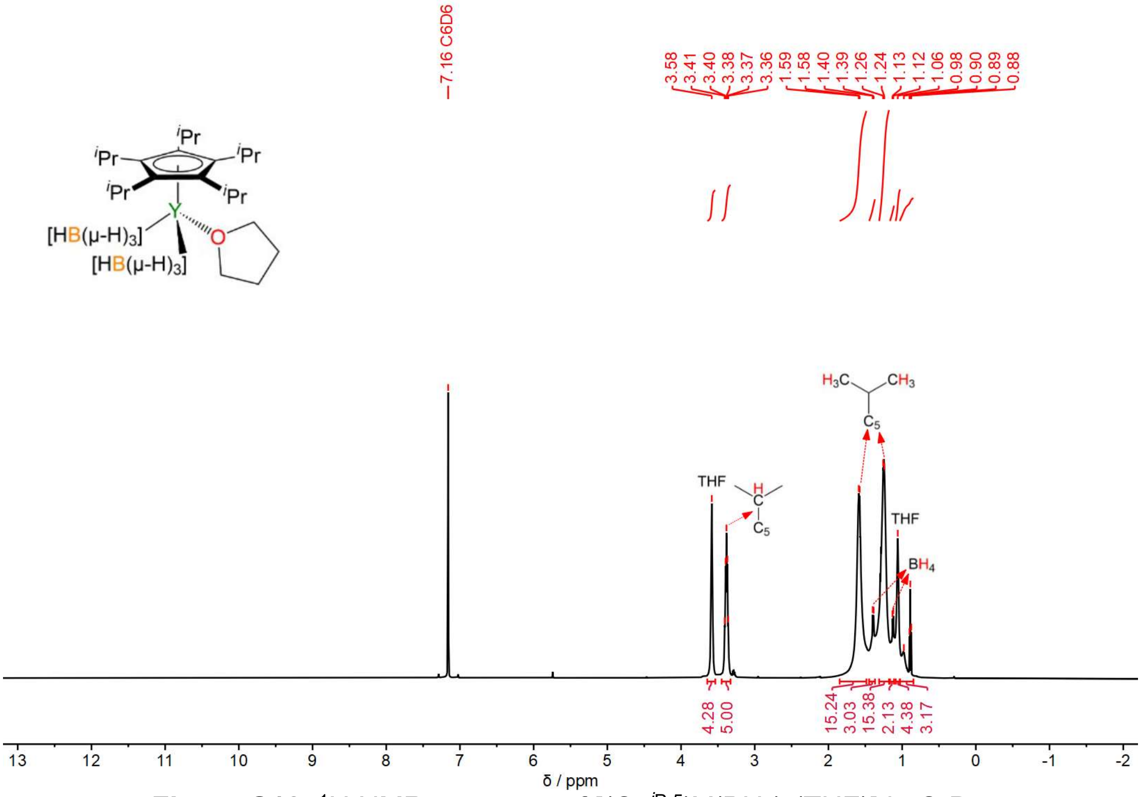

Figure S12. ^{1}H NMR spectrum of $[(Cp^{iPr5})Y(BH_4)_2(THF)]$ in C_6D_6.

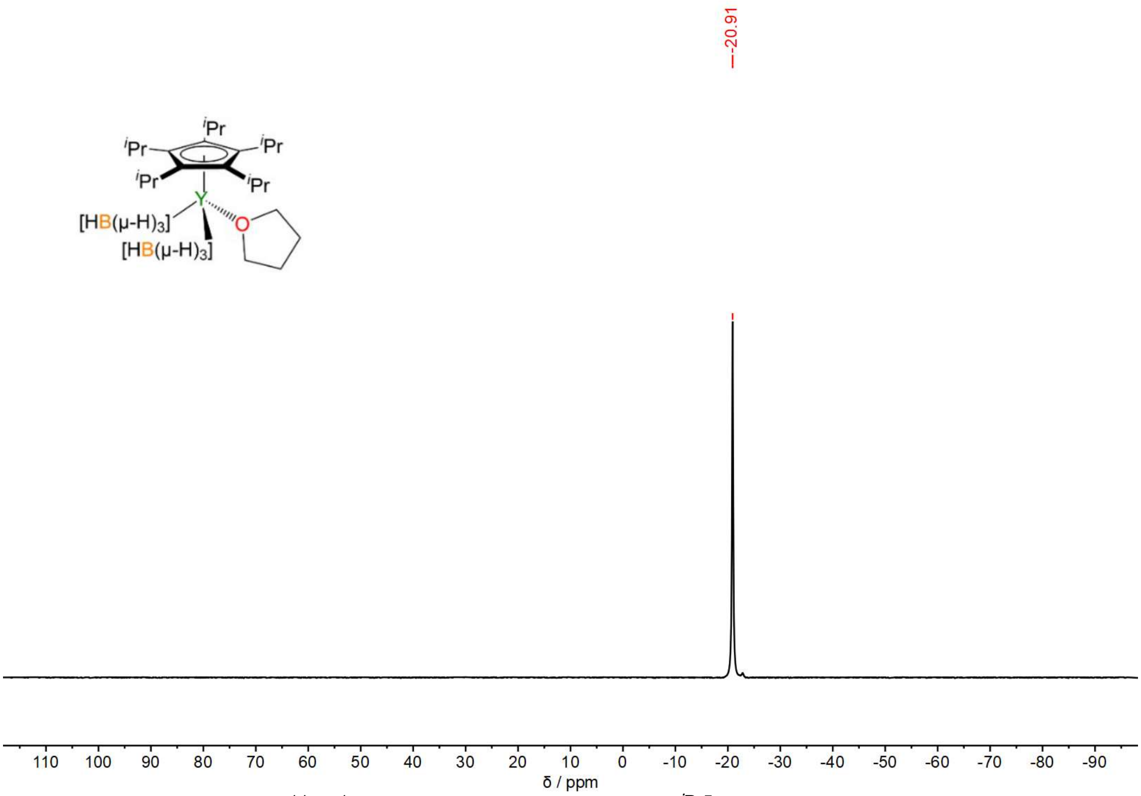

Figure S13. $^{11}B\{^{1}H\}$ NMR spectrum of $[(Cp^{iPr5})Y(BH_4)_2(THF)]$ in C_6D_6.

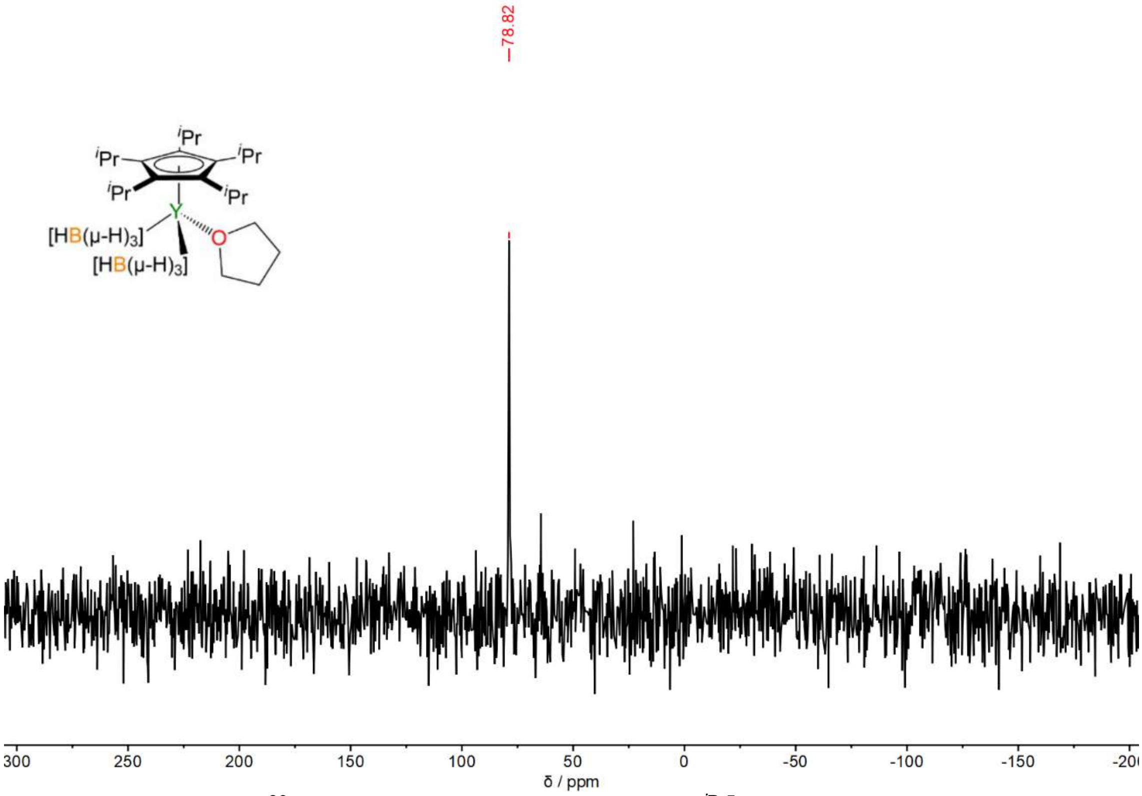

Figure S14. ^{89}Y INEPT NMR spectrum of [(Cp^{iPr5})Y(BH$_4$)$_2$(THF)] in C_6D_6.

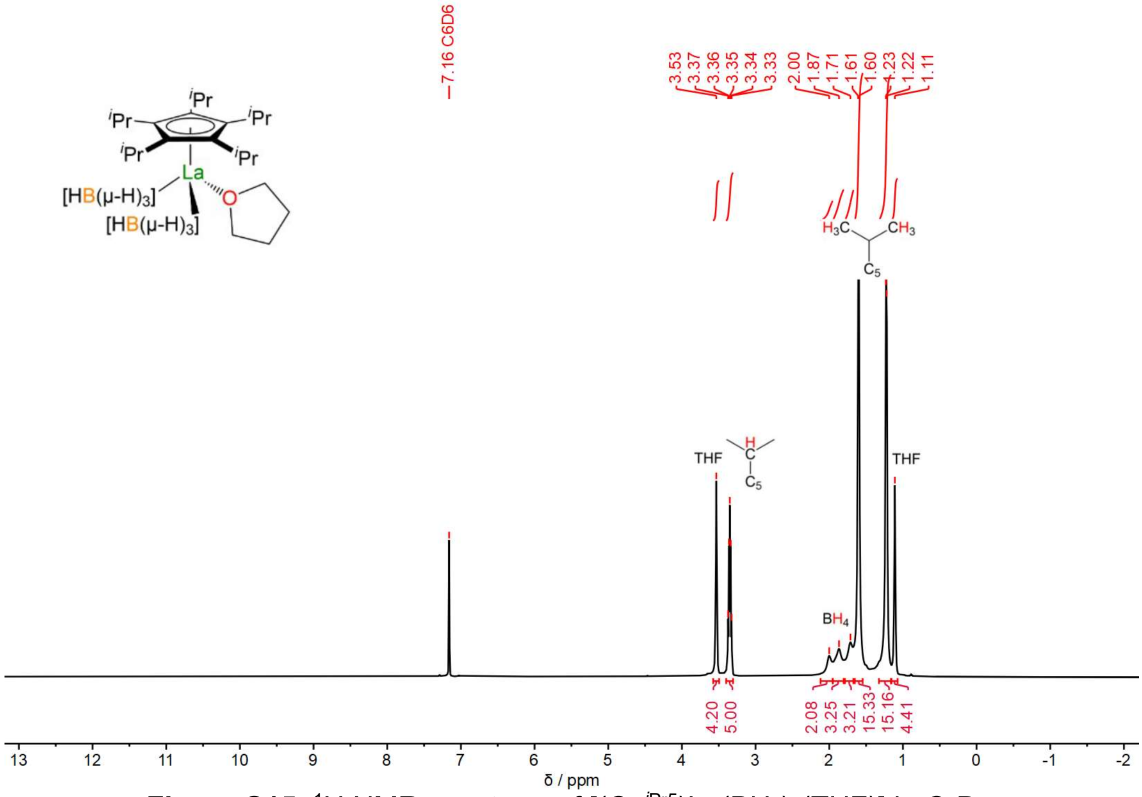

Figure S15. ^{1}H NMR spectrum of [(Cp^{iPr5})La(BH$_4$)$_2$(THF)] in C_6D_6.

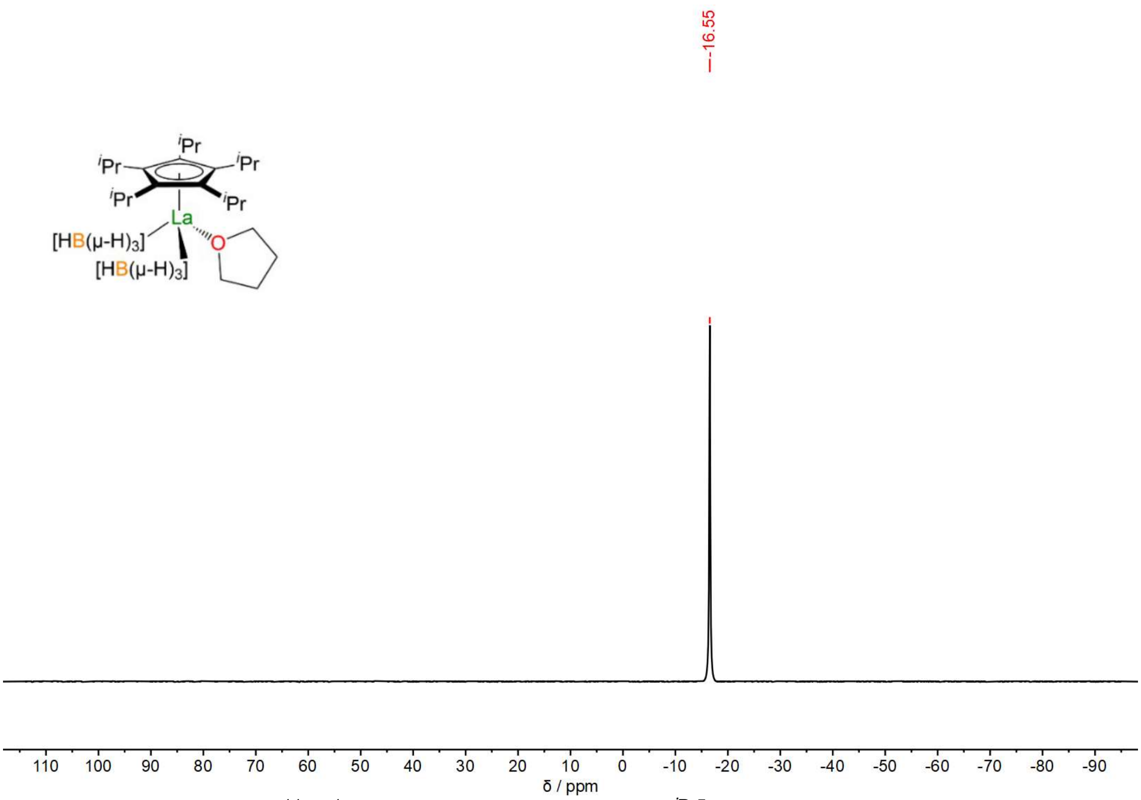

Figure S16. $^{11}B\{^1H\}$ NMR spectrum of $[(Cp^{iPr5})La(BH_4)_2(THF)]$ in C_6D_6.

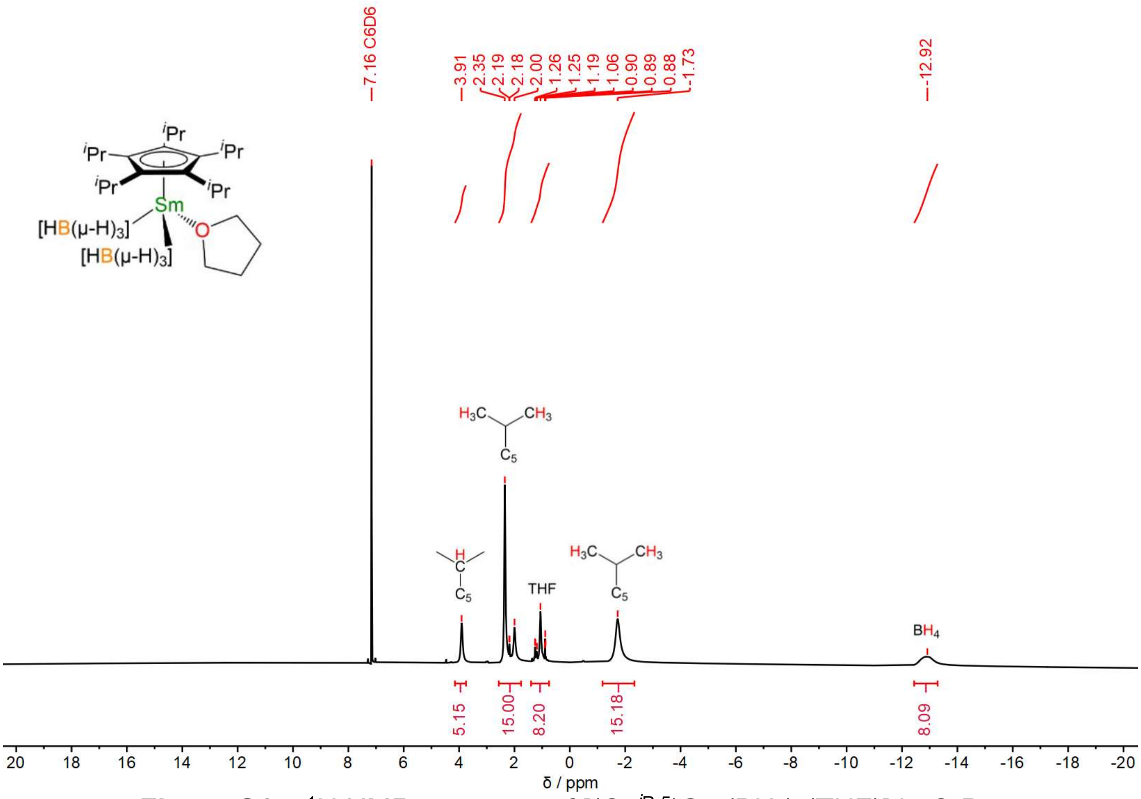

Figure S17. ^1H NMR spectrum of $[(Cp^{iPr5})Sm(BH_4)_2(THF)]$ in C_6D_6.

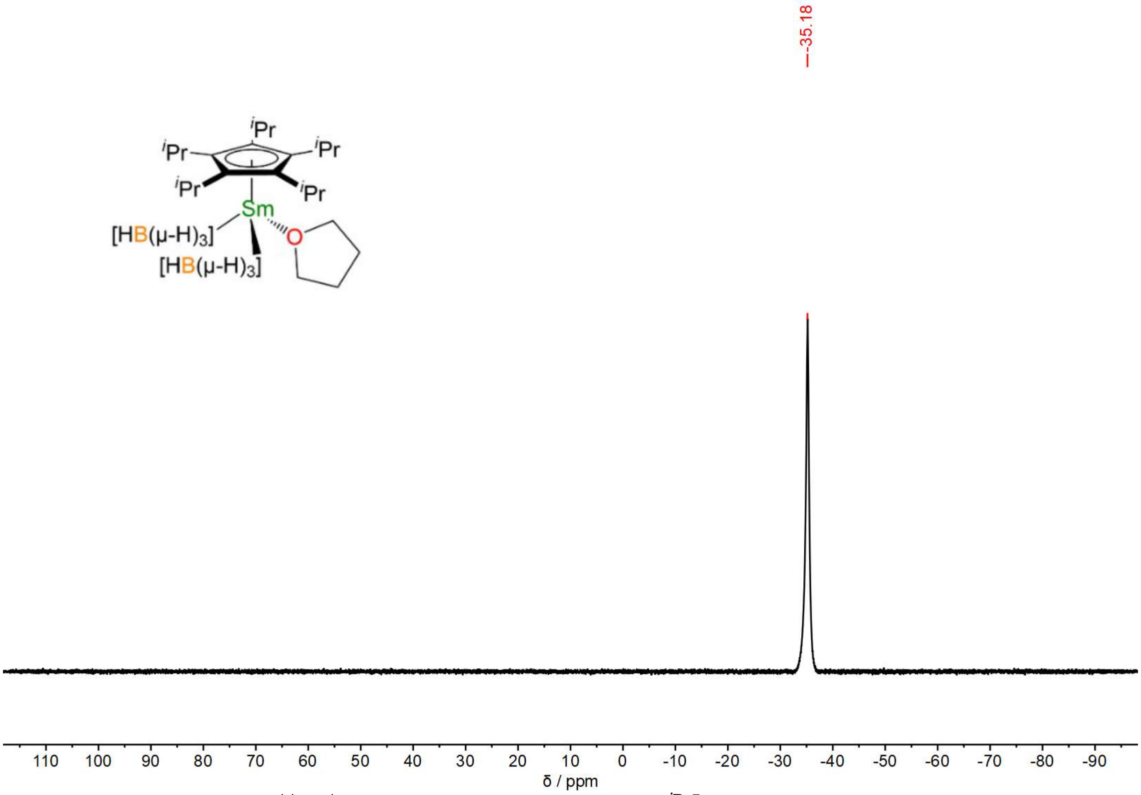

Figure S18. $^{11}B\{^{1}H\}$ NMR spectrum of $[(Cp^{iPr5})Sm(BH_4)_2(THF)]$ in C_6D_6.

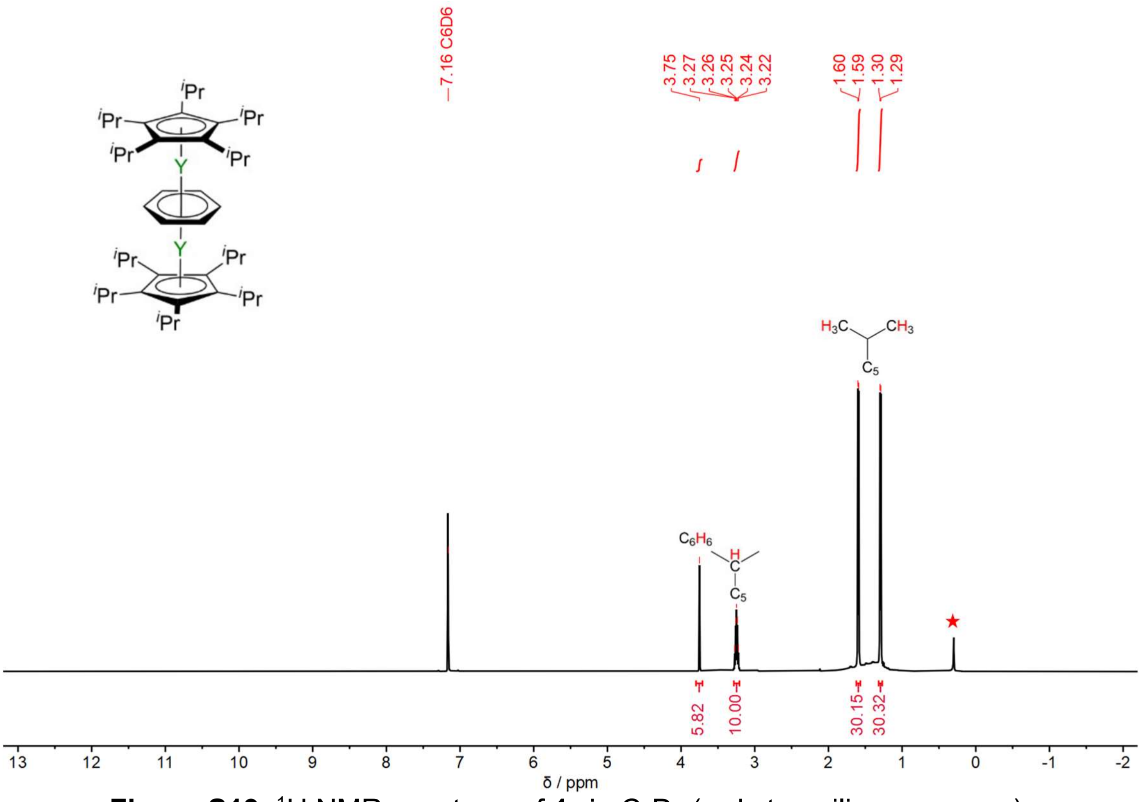

Figure S19. ^{1}H NMR spectrum of $\mathbf{1_Y}$ in C_6D_6 (red star: silicone grease).

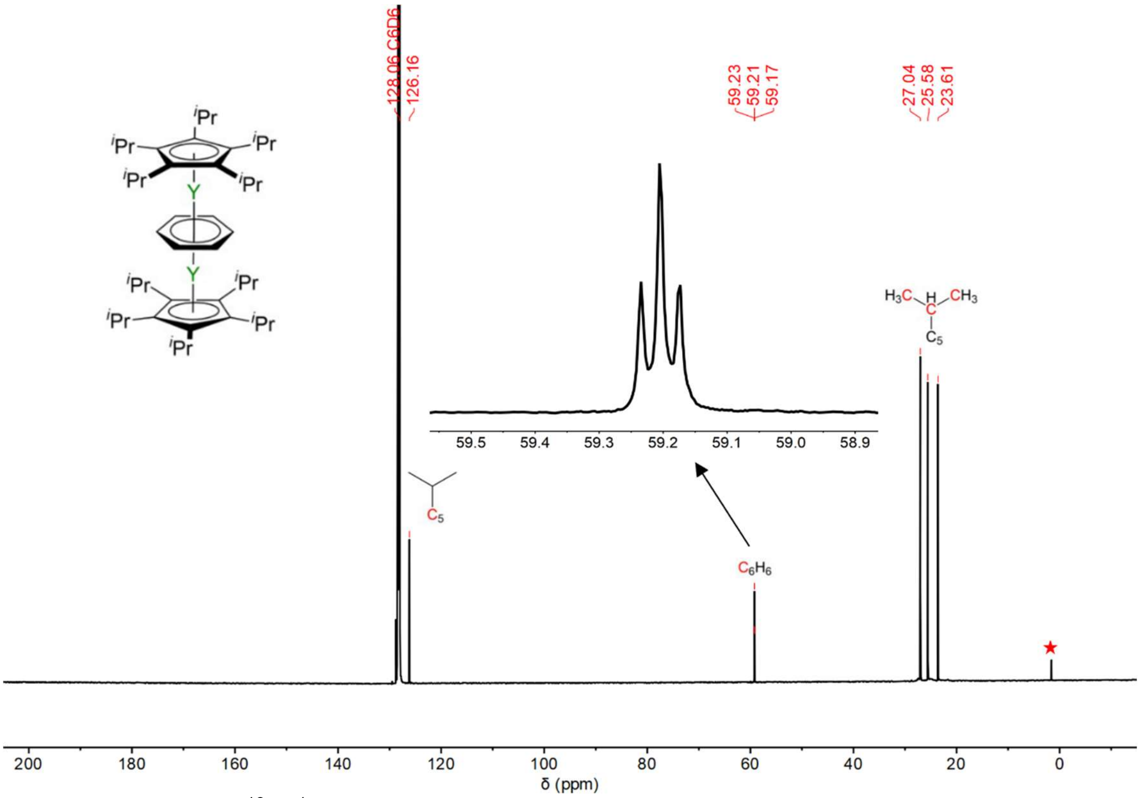

Figure S20. $^{13}C\{^1H\}$ NMR spectrum of $\mathbf{1_Y}$ in C_6D_6 (red star: silicone grease).

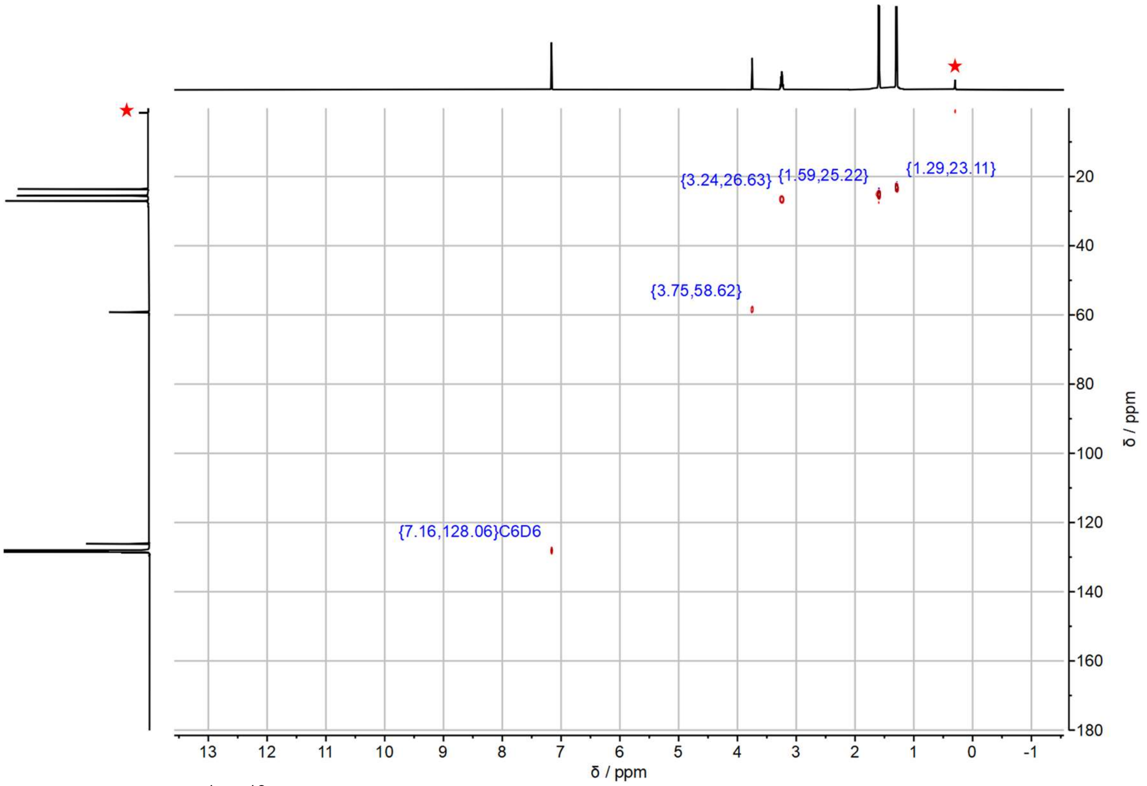

Figure S21. ^1H-^{13}C HSQC NMR spectrum of $\mathbf{1_Y}$ in C_6D_6 (red star: silicone grease).

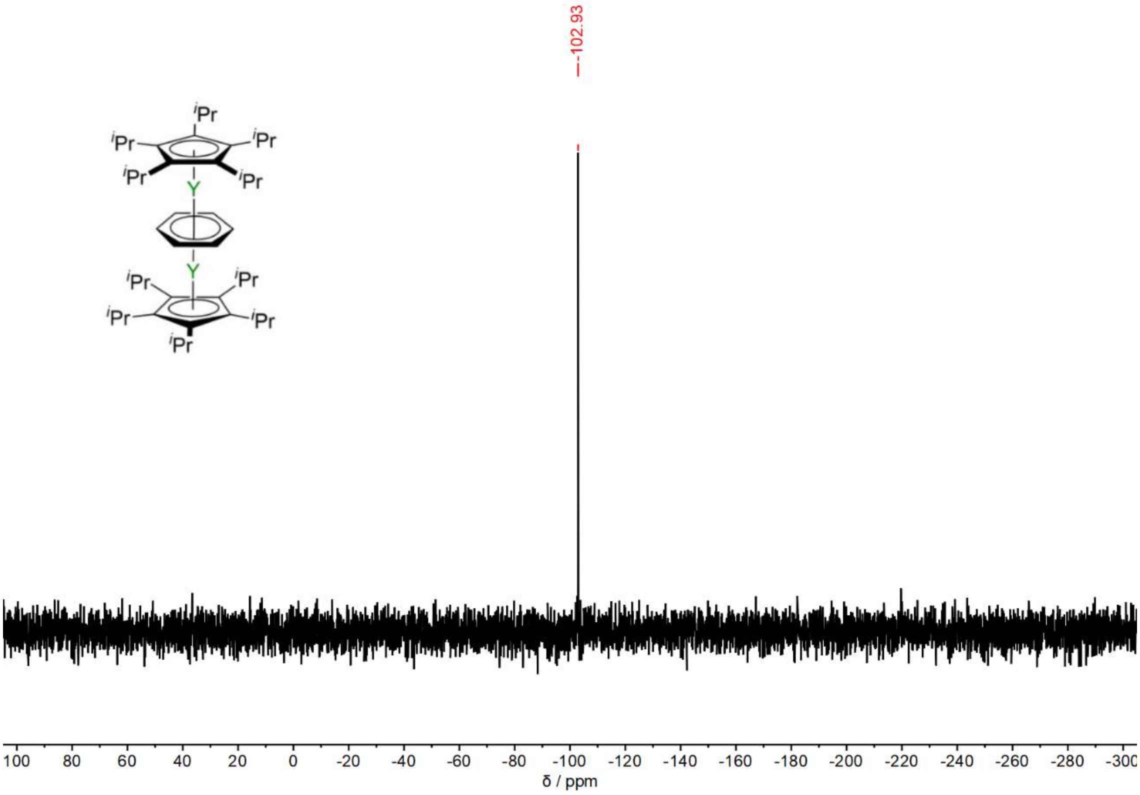

Figure S22. ^{89}Y INEPT NMR spectrum of $\mathbf{1_Y}$ in C_6D_6.

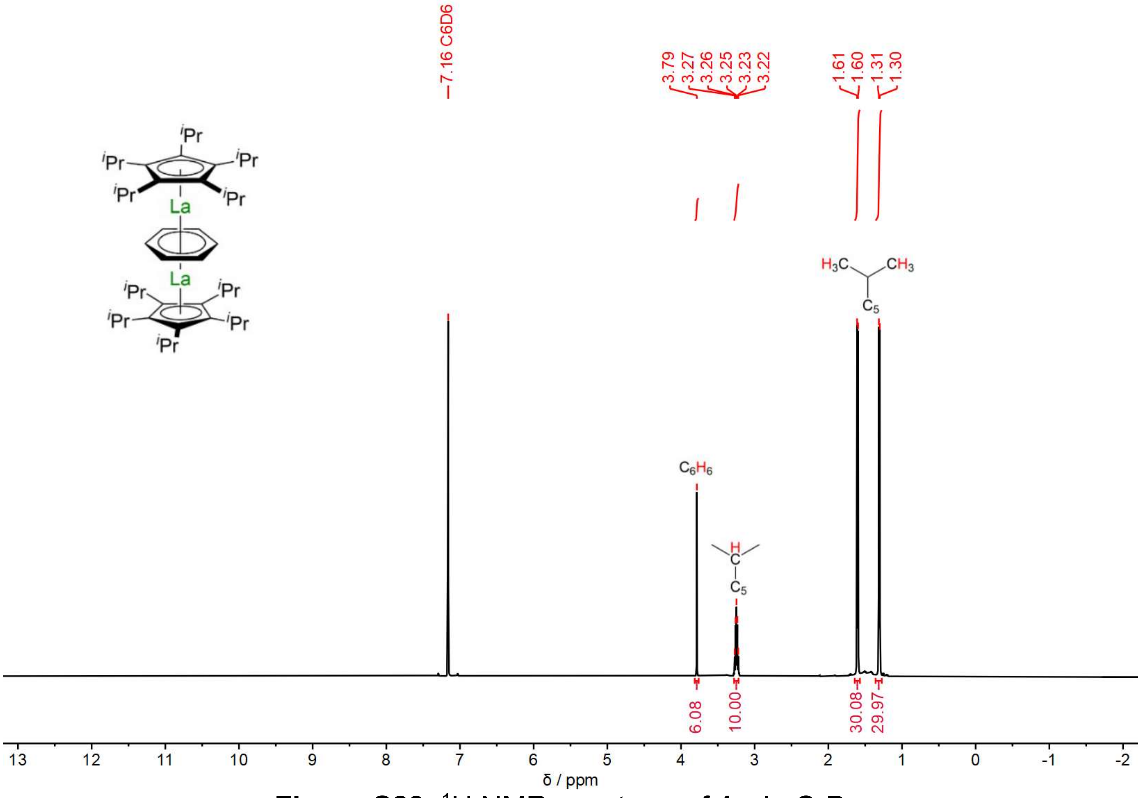

Figure S23. ^{1}H NMR spectrum of $\mathbf{1_{La}}$ in C_6D_6.

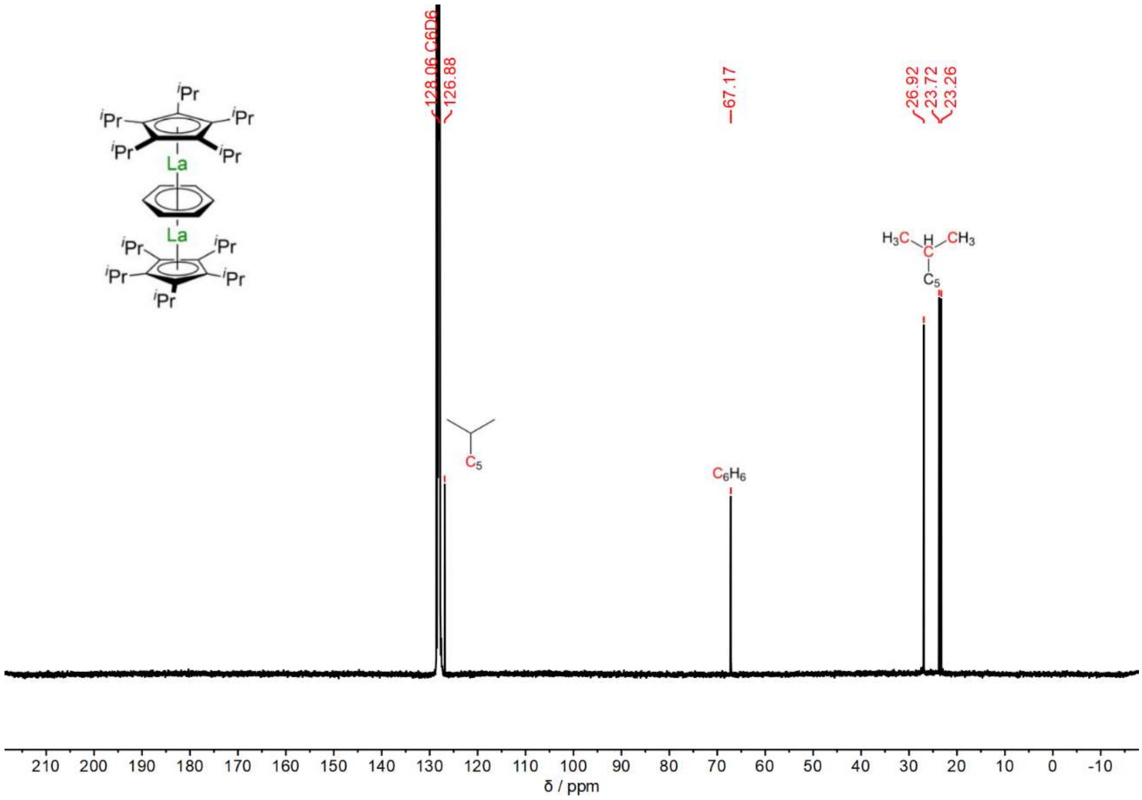

Figure S24. $^{13}C\{^{1}H\}$ NMR spectrum of $\mathbf{1_{La}}$ in C_6D_6.

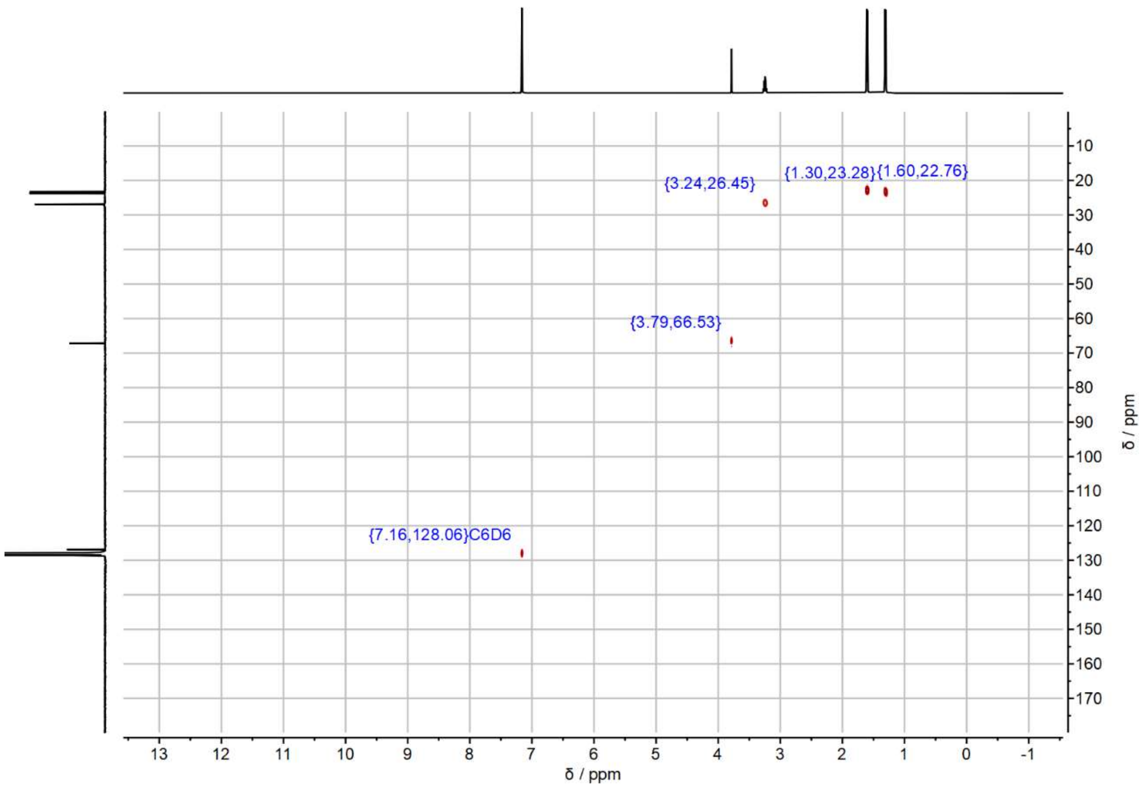

Figure S25. ^{1}H-^{13}C HSQC NMR spectrum of $\mathbf{1_{La}}$ in C_6D_6.

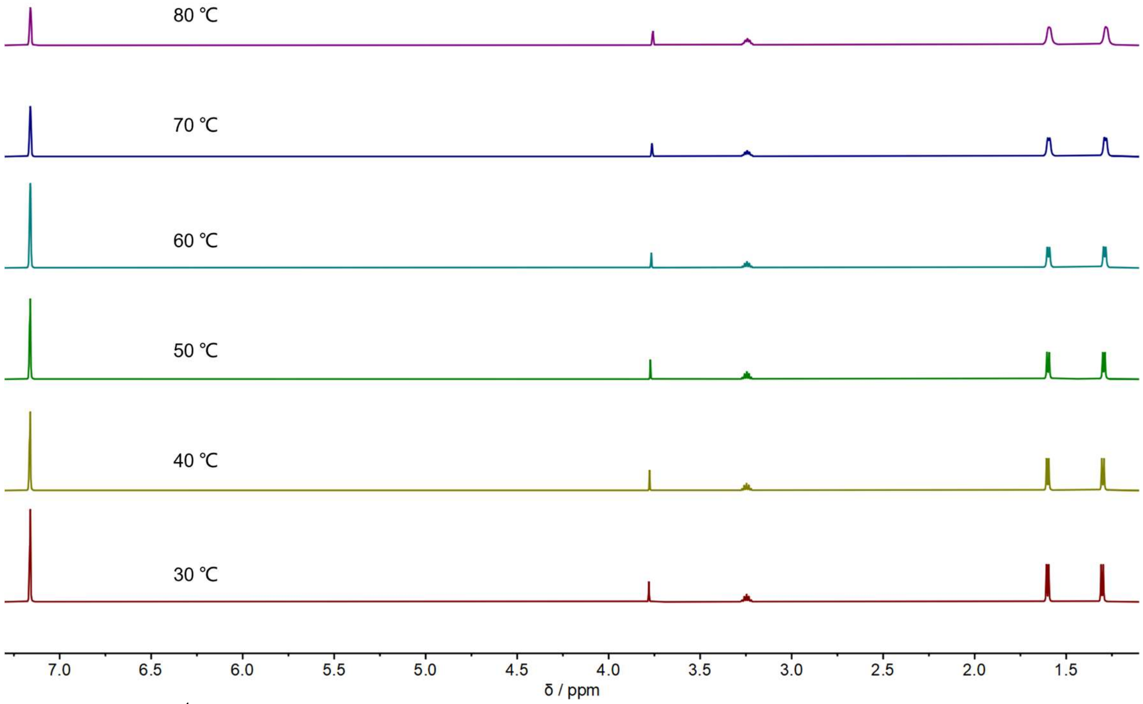

Figure S26. ^{1}H NMR spectra of $\mathbf{1_{La}}$ in C_6D_6 measured at 30, 40, 50, 60, 70 and 80 °C.

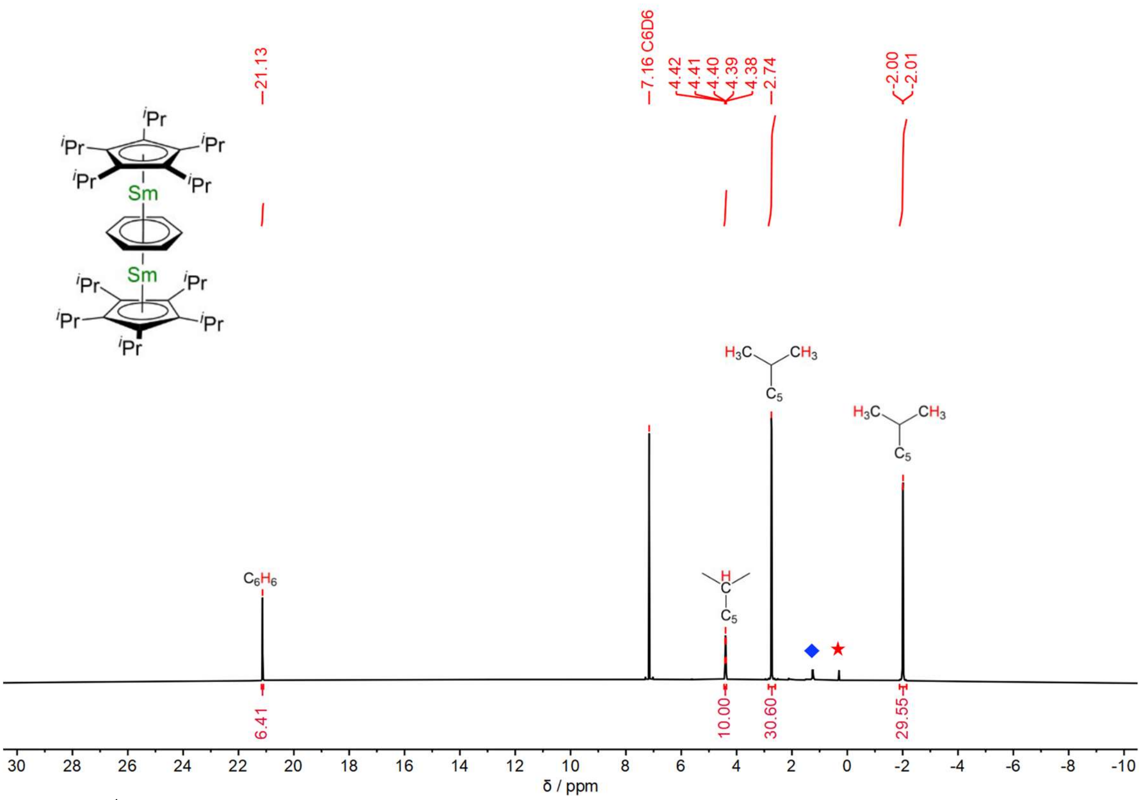

Figure S27. ^{1}H NMR spectrum of $\mathbf{1_{Sm}}$ in C_6D_6 (blue rhomboid: hexane, red star: silicone grease).

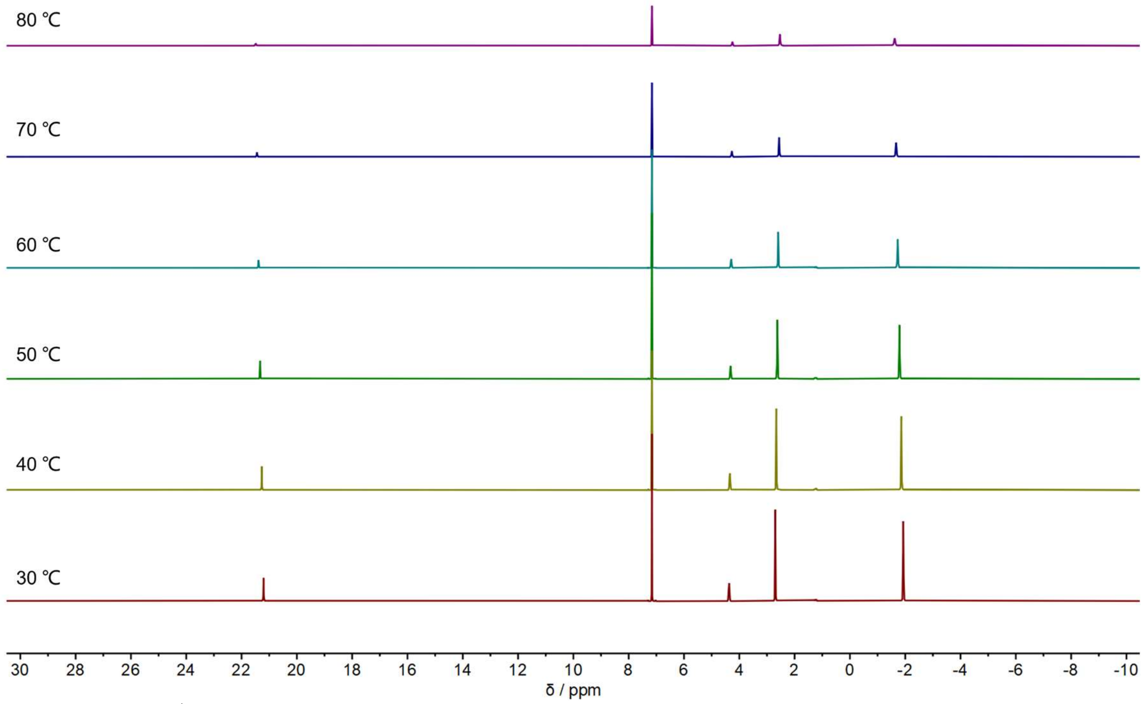

Figure S28. ^{1}H NMR spectra of $\mathbf{1_{Sm}}$ in C_6D_6 measured at 30, 40, 50, 60, 70 and 80 °C.

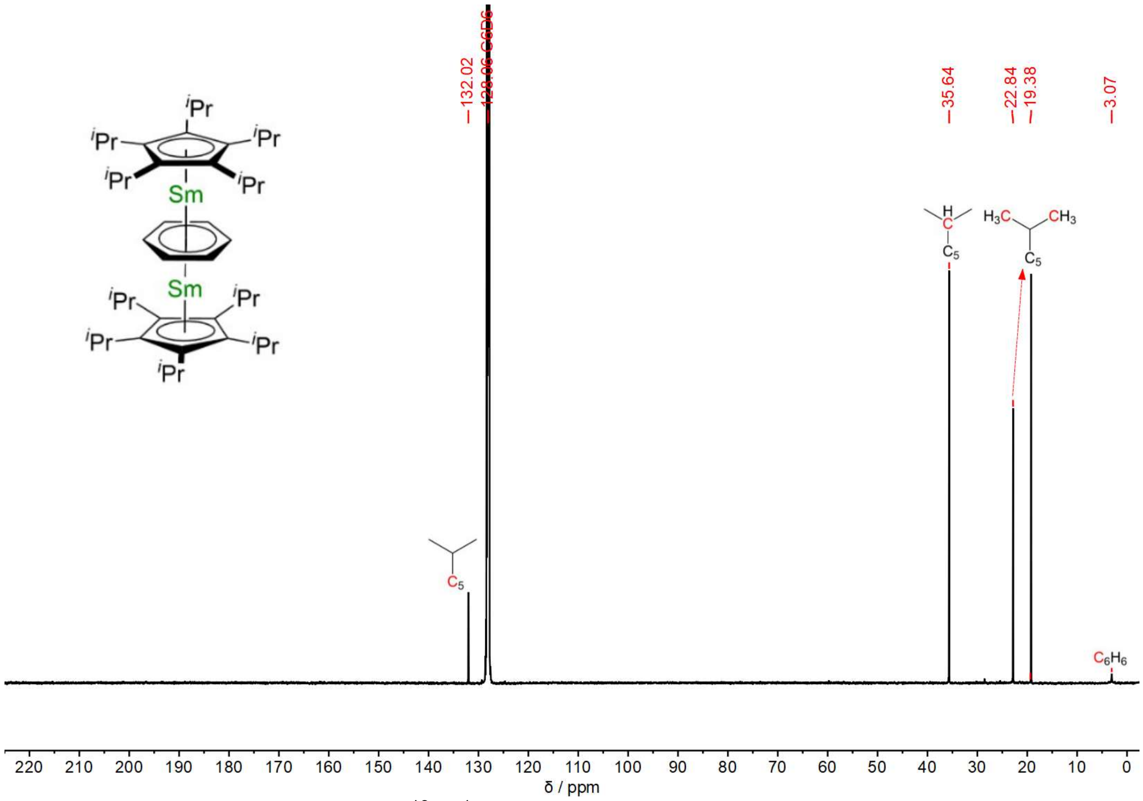

Figure S29. $^{13}C\{^{1}H\}$ NMR spectrum of $\mathbf{1_{Sm}}$ in C_6D_6.

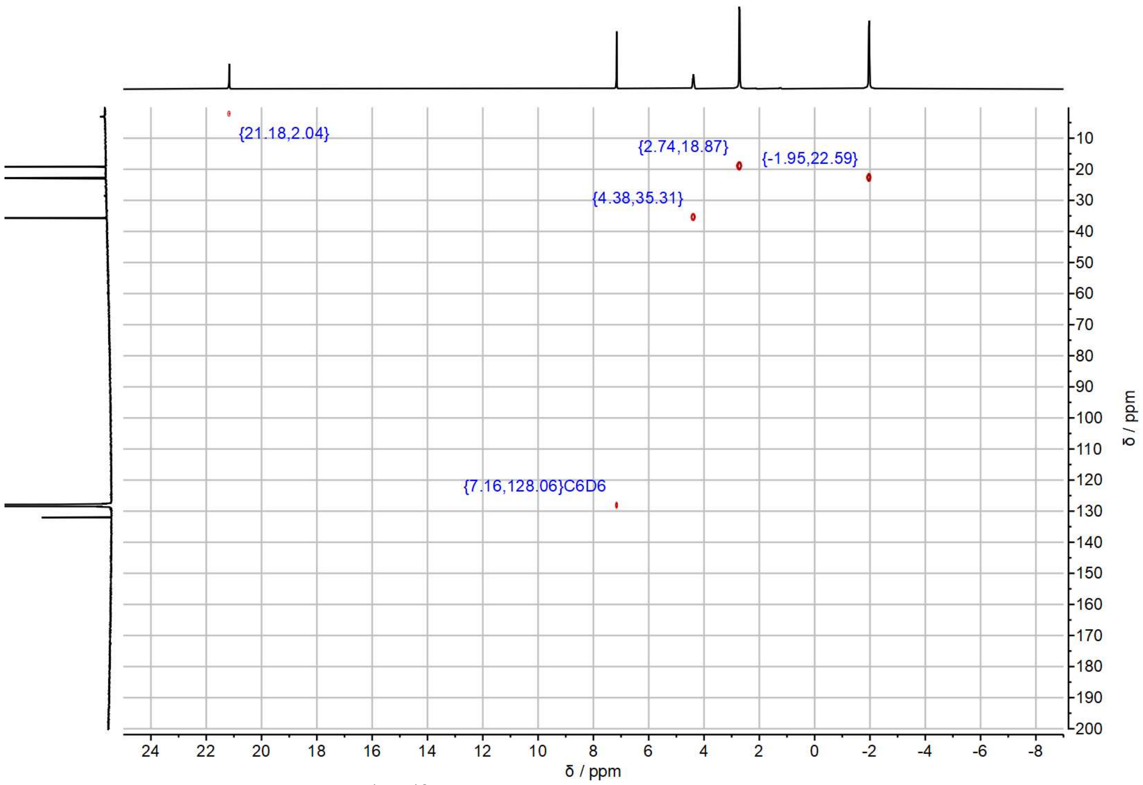

Figure S30. ^{1}H-^{13}C HSQC NMR spectrum of $\mathbf{1_{Sm}}$ in C_6D_6.

UV/vis/NIR Spectroscopy

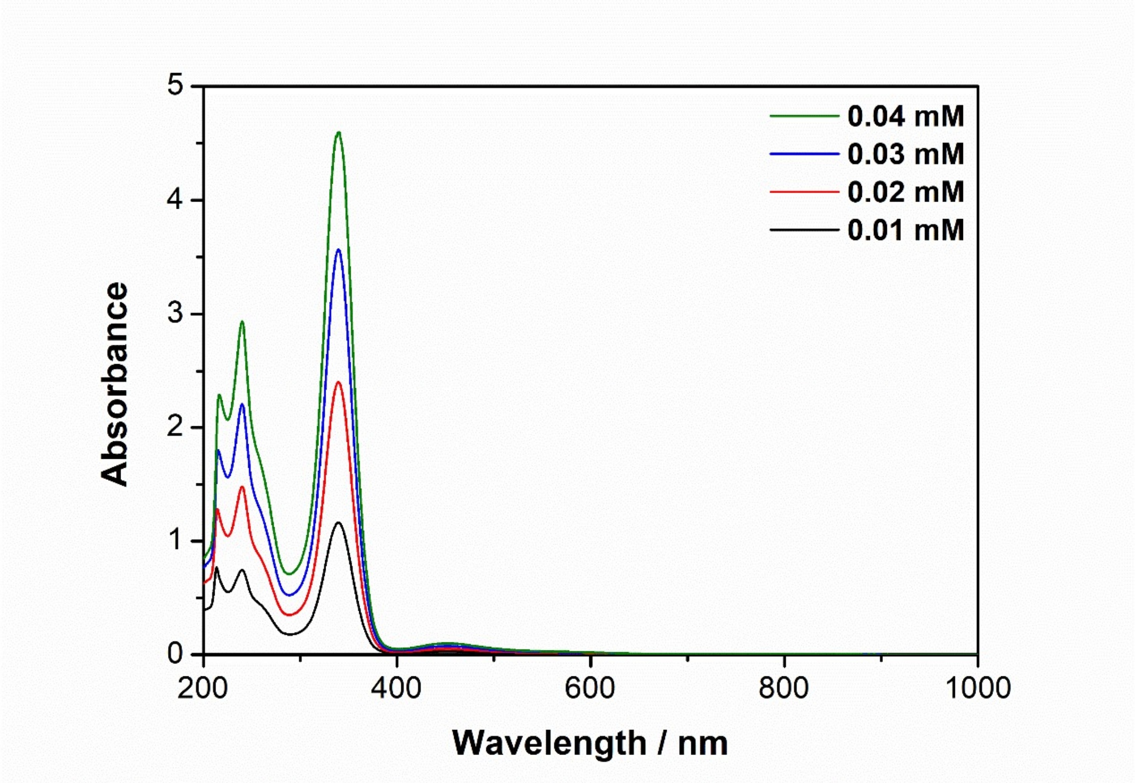

Figure S31. UV/vis/NIR spectrum of $\mathbf{1}_{\mathrm{Y}}$ in hexane at different concentrations. Significant absorptions occur at λ = 216, 240, 340 and 455 nm.

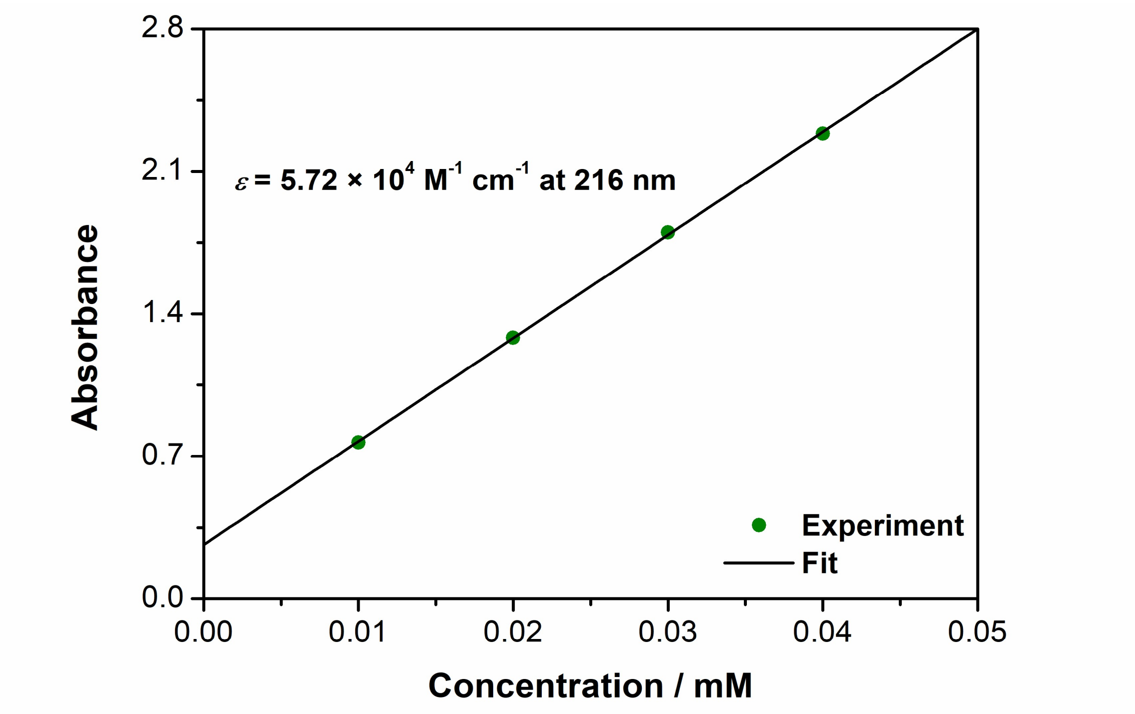

Figure S32. Plot of absorbance versus concentration for $\mathbf{1}_{\mathrm{Y}}$ at 216 nm in the UV/vis/NIR spectrum. The green points are from the UV/vis/NIR spectrum. The solid black line is the best fit to the data to extract the extinction coefficient.

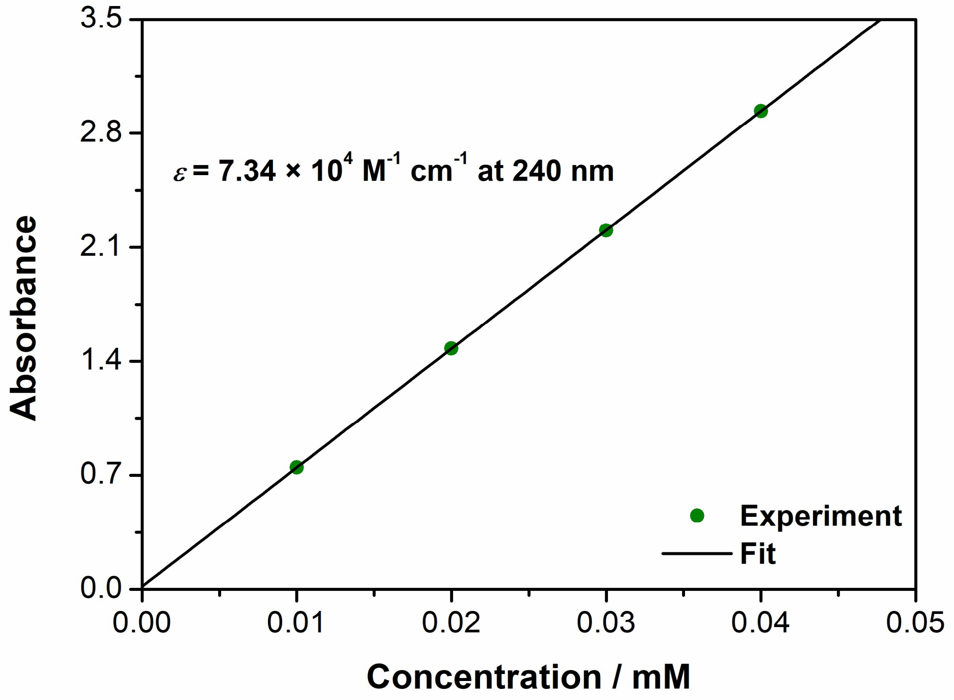

Figure S33. Plot of absorbance versus concentration for **1**$_Y$ at 240 nm in the UV/vis/NIR spectrum. The green points are from the UV/vis/NIR spectrum. The solid black line is the best fit to the data to extract the extinction coefficient.

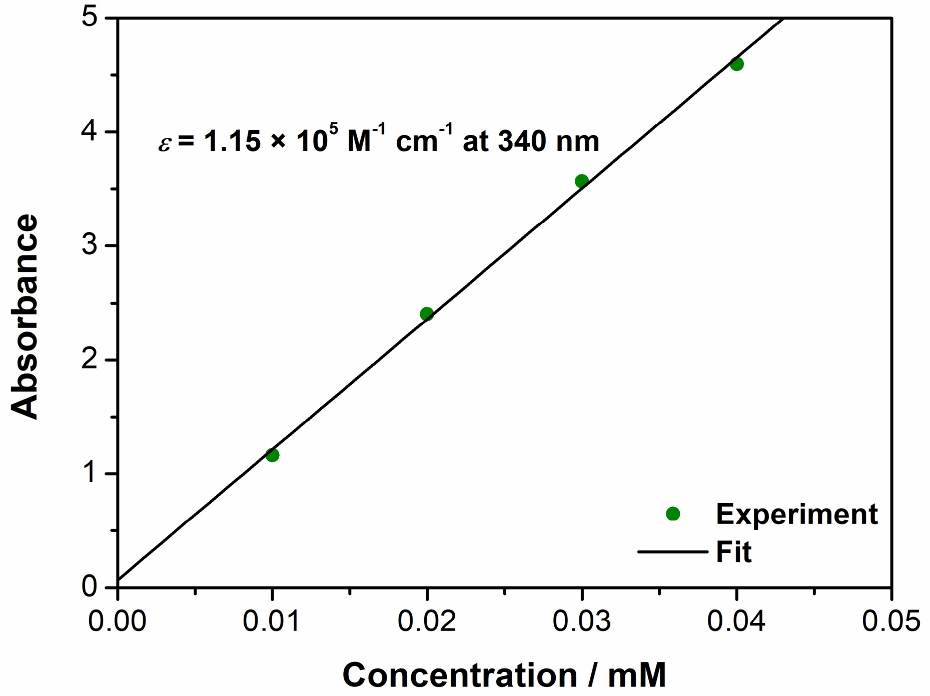

Figure S34. Plot of absorbance versus concentration for **1**$_Y$ at 340 nm in the UV/vis/NIR spectrum. The green points are from the UV/vis/NIR spectrum. The solid black line is the best fit to the data to extract the extinction coefficient.

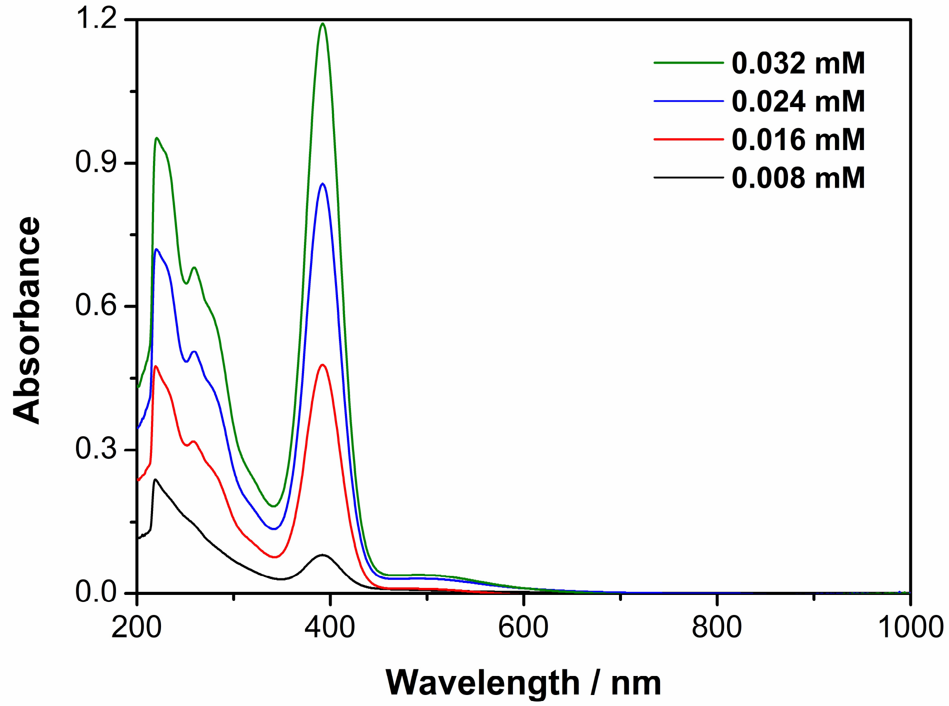

Figure S35. UV/vis/NIR spectrum of $\mathbf{1}_{\mathbf{La}}$ in hexane at different concentrations. Significant absorptions occur at λ = 220, 260 and 392 nm.

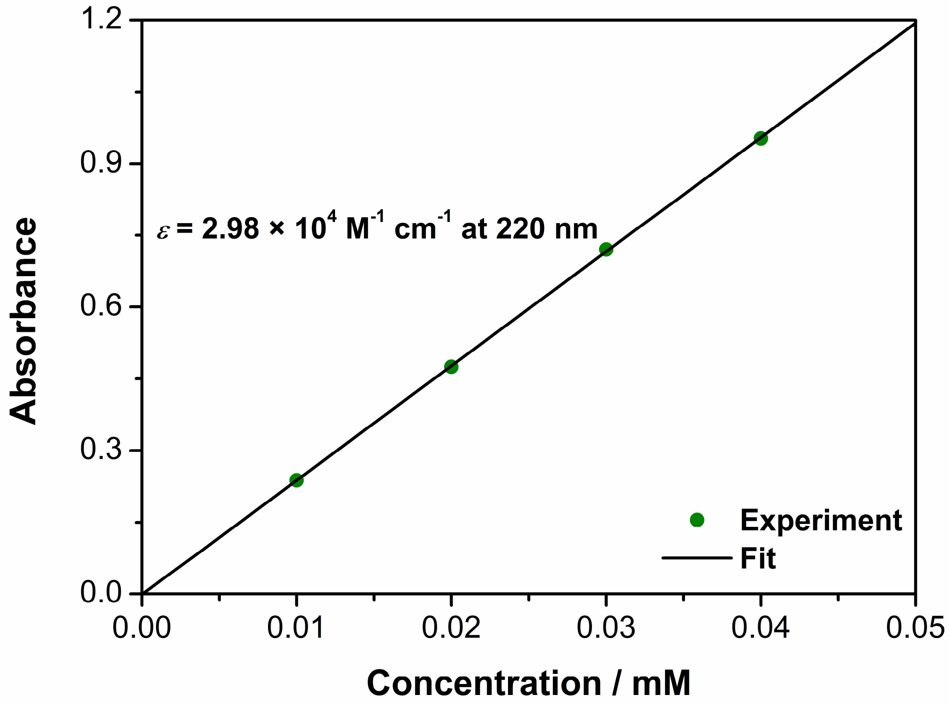

Figure S36. Plot of absorbance versus concentration for $\mathbf{1}_{\mathbf{La}}$ at 220 nm in the UV/vis/NIR spectrum. The green points are from the UV/vis/NIR spectrum. The solid black line is the best fit to the data to extract the extinction coefficient.

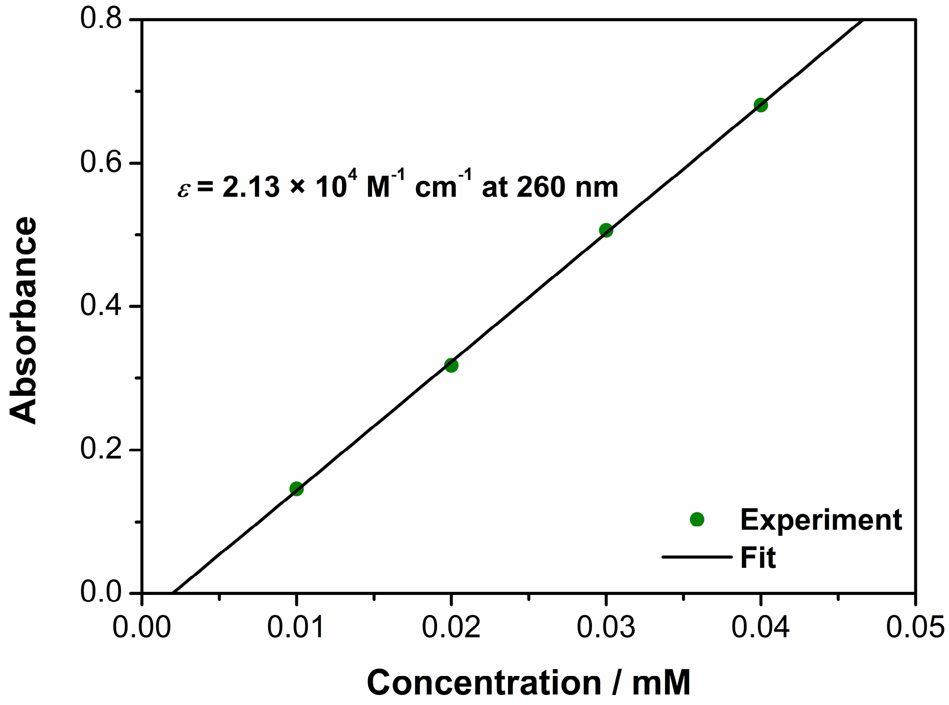

Figure S37. Plot of absorbance versus concentration for $\mathbf{1}_{La}$ at 260 nm in the UV/vis/NIR spectrum. The green points are from the UV/vis/NIR spectrum. The solid black line is the best fit to the data to extract the extinction coefficient.

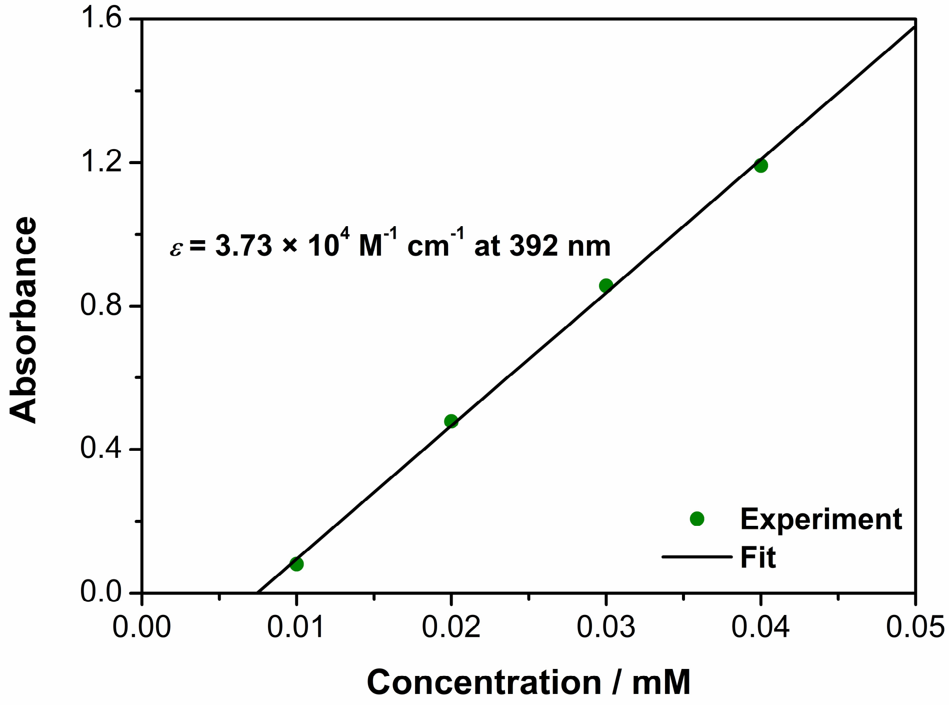

Figure S38. Plot of absorbance versus concentration for $\mathbf{1}_{La}$ at 392 nm in the UV/vis/NIR spectrum. The green points are from the UV/vis/NIR spectrum. The solid black line is the best fit to the data to extract the extinction coefficient.

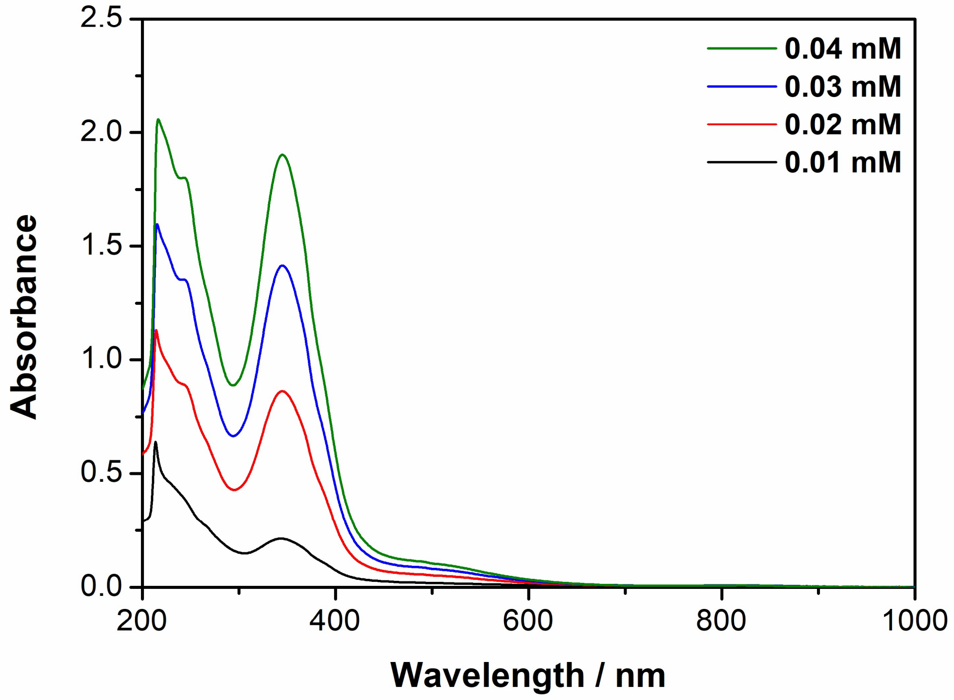

Figure S39. UV/vis/NIR spectrum of $\mathbf{1_{Sm}}$ in hexane at different concentrations. Significant absorptions occur at λ = 216, 244 and 345 nm.

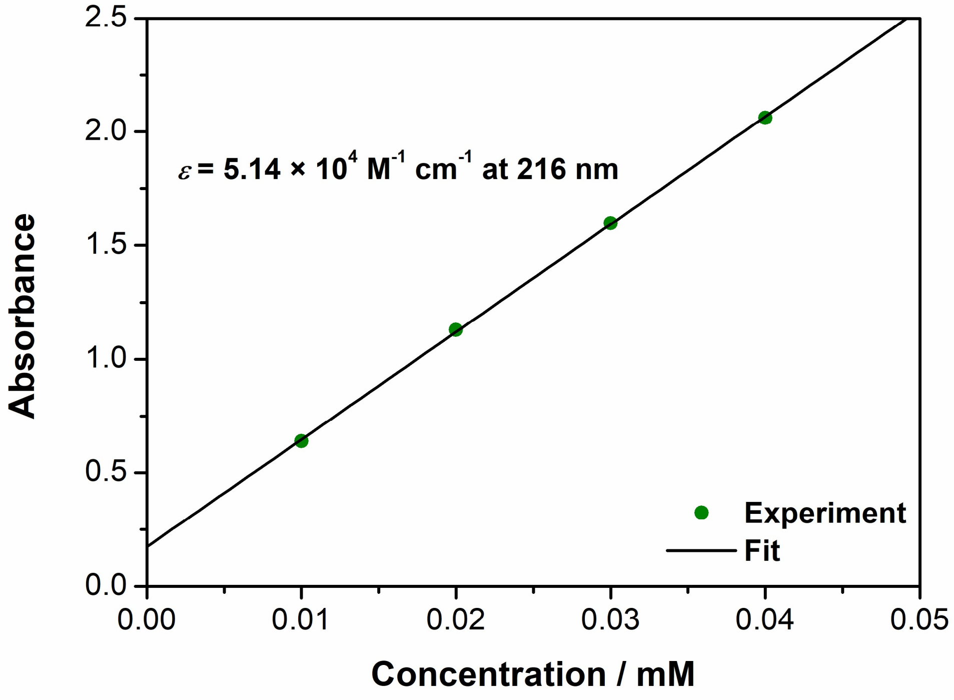

Figure S40. Plot of absorbance versus concentration for $\mathbf{1_{Sm}}$ at 216 nm in the UV/vis/NIR spectrum. The green points are from the UV/vis/NIR spectrum. The solid black line is the best fit to the data to extract the extinction coefficient.

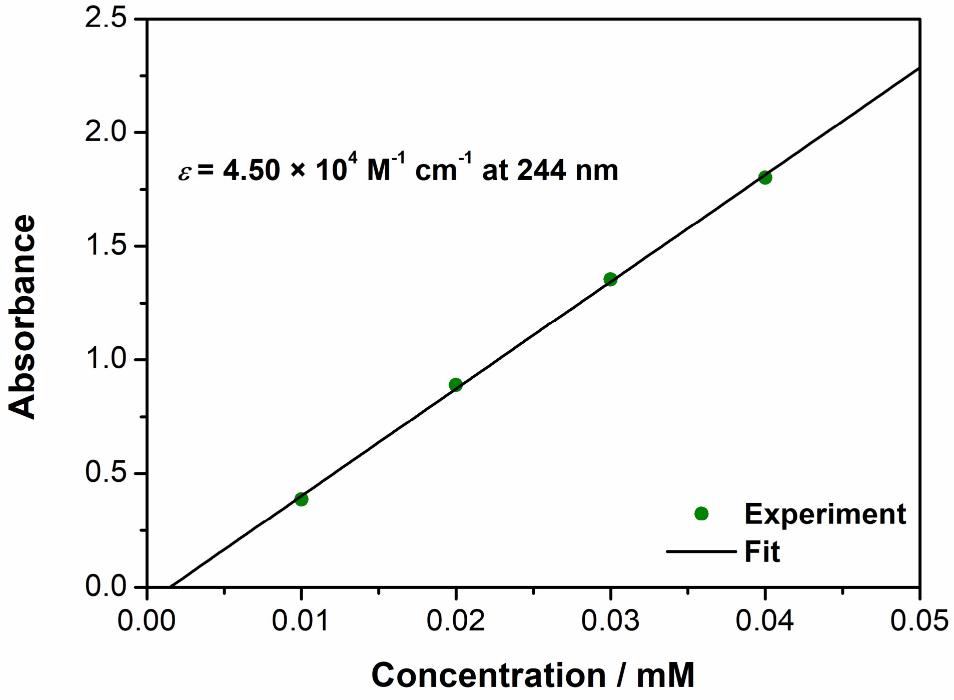

Figure S41. Plot of absorbance versus concentration for $\mathbf{1}_{\mathrm{Sm}}$ at 244 nm in the UV/vis/NIR spectrum. The green points are from the UV/vis/NIR spectrum. The solid black line is the best fit to the data to extract the extinction coefficient.

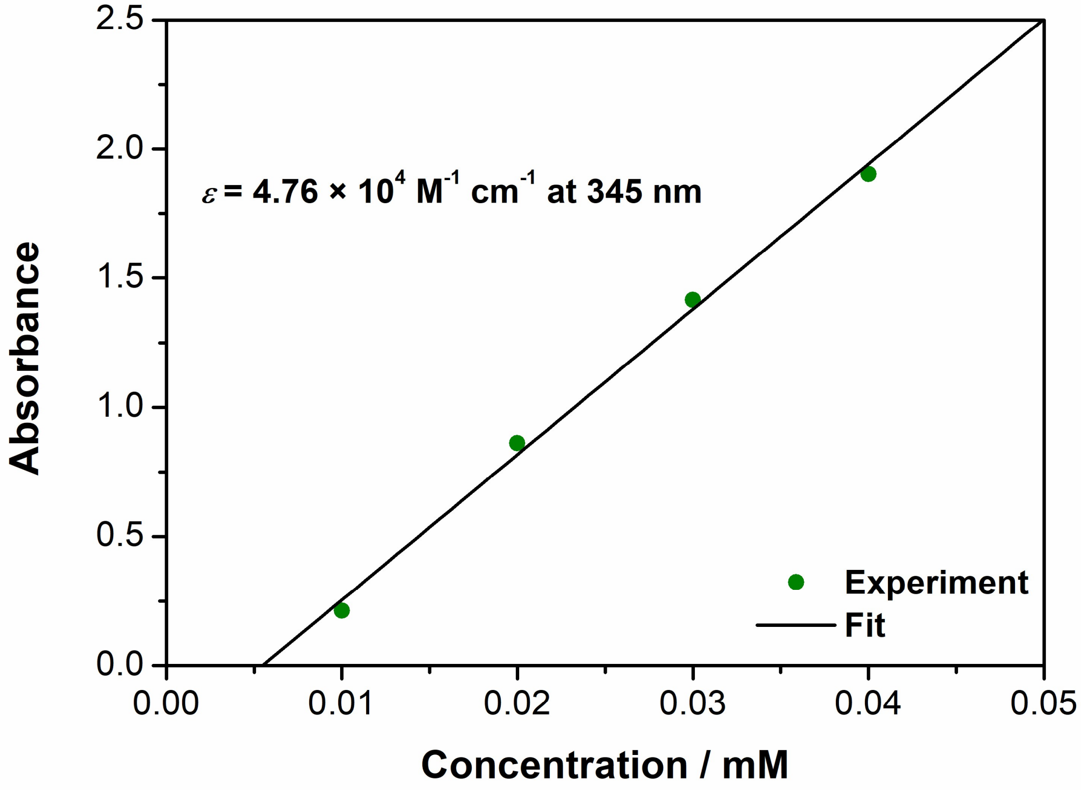

Figure S42. Plot of absorbance versus concentration for $\mathbf{1}_{\mathrm{Sm}}$ at 345 nm in the UV/vis/NIR spectrum. The green points are from the UV/vis/NIR spectrum. The solid black line is the best fit to the data to extract the extinction coefficient.

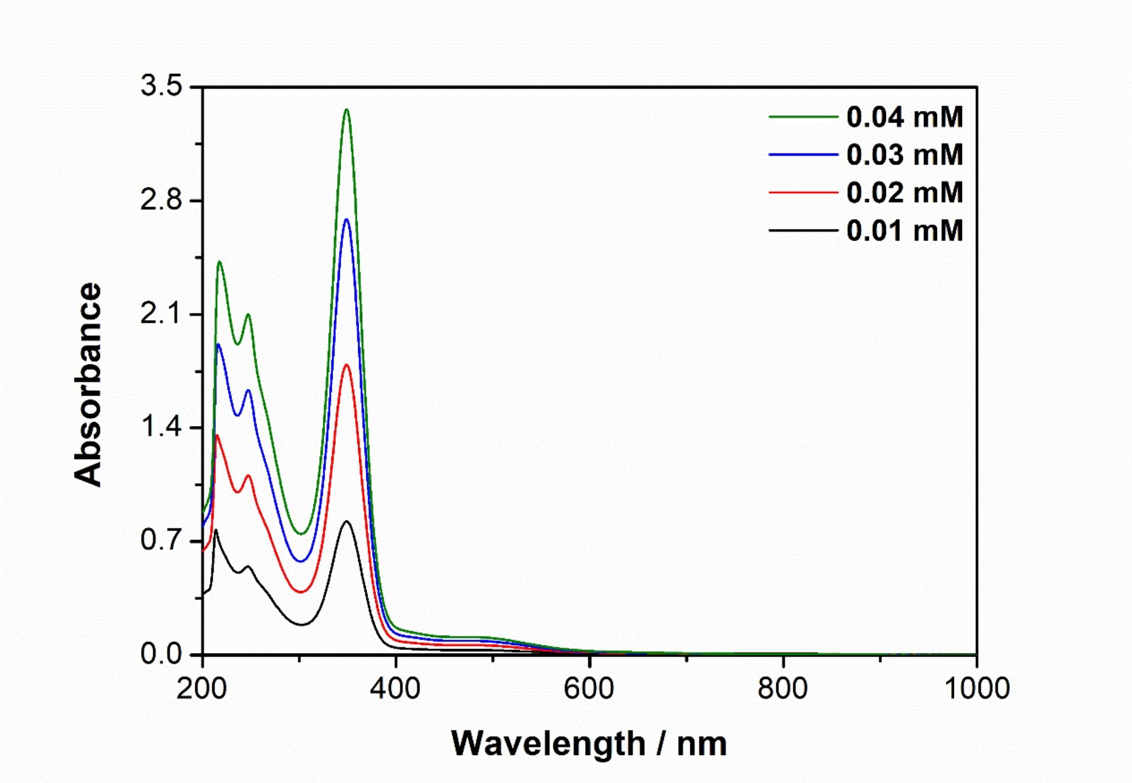

Figure S43. UV/vis/NIR spectrum of $\mathbf{1}_{Gd}$ in hexane at different concentrations. Significant absorptions occur at λ = 216, 247, 349 and 496 nm.

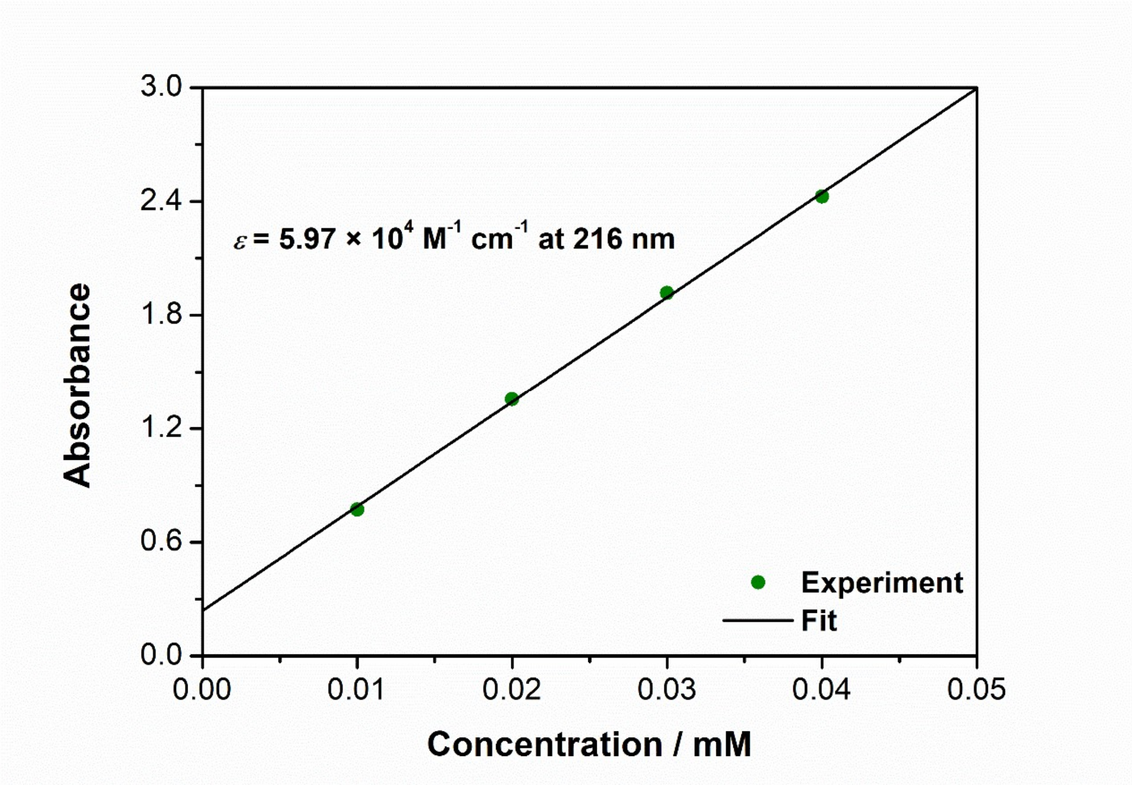

Figure S44. Plot of absorbance versus concentration for $\mathbf{1}_{Gd}$ at 216 nm in the UV/vis/NIR spectrum. The green points are from the UV/vis/NIR spectrum. The solid black line is the best fit to the data to extract the extinction coefficient.

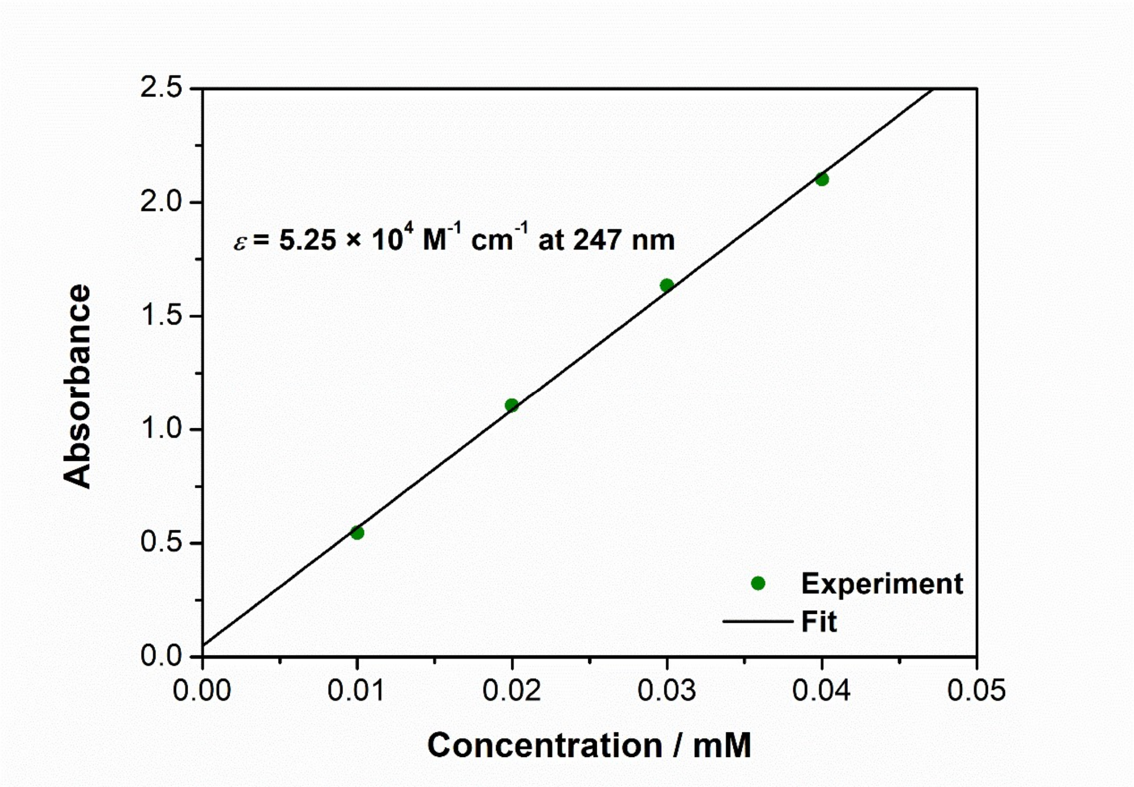

Figure S45. Plot of absorbance versus concentration for $\mathbf{1}_{Gd}$ at 247 nm in the UV/vis/NIR spectrum. The green points are from the UV/vis/NIR spectrum. The solid black line is the best fit to the data to extract the extinction coefficient.

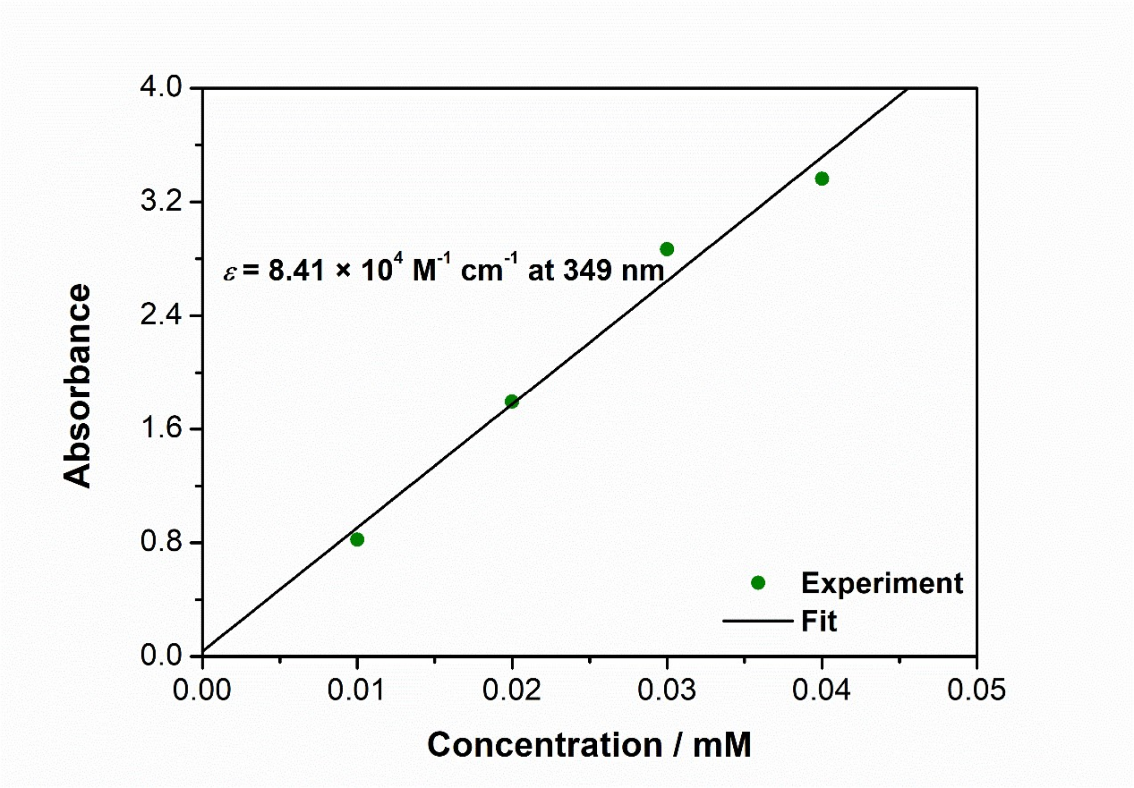

Figure S46. Plot of absorbance versus concentration for $\mathbf{1}_{Gd}$ at 349 nm in the UV/vis/NIR spectrum. The green points are from the UV/vis/NIR spectrum. The solid black line is the best fit to the data to extract the extinction coefficient.

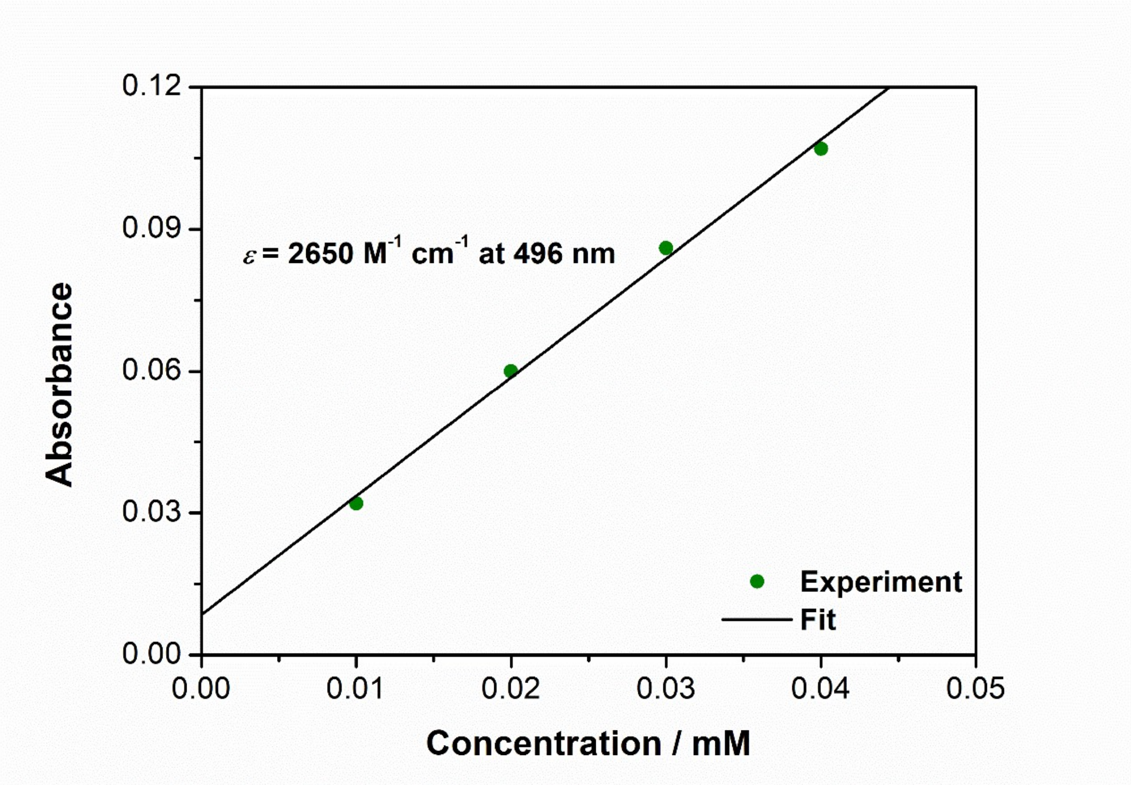

Figure S47. Plot of absorbance versus concentration for **1**$_{Gd}$ at 496 nm in the UV/vis/NIR spectrum. The green points are from the UV/vis/NIR spectrum. The solid black line is the best fit to the data to extract the extinction coefficient.

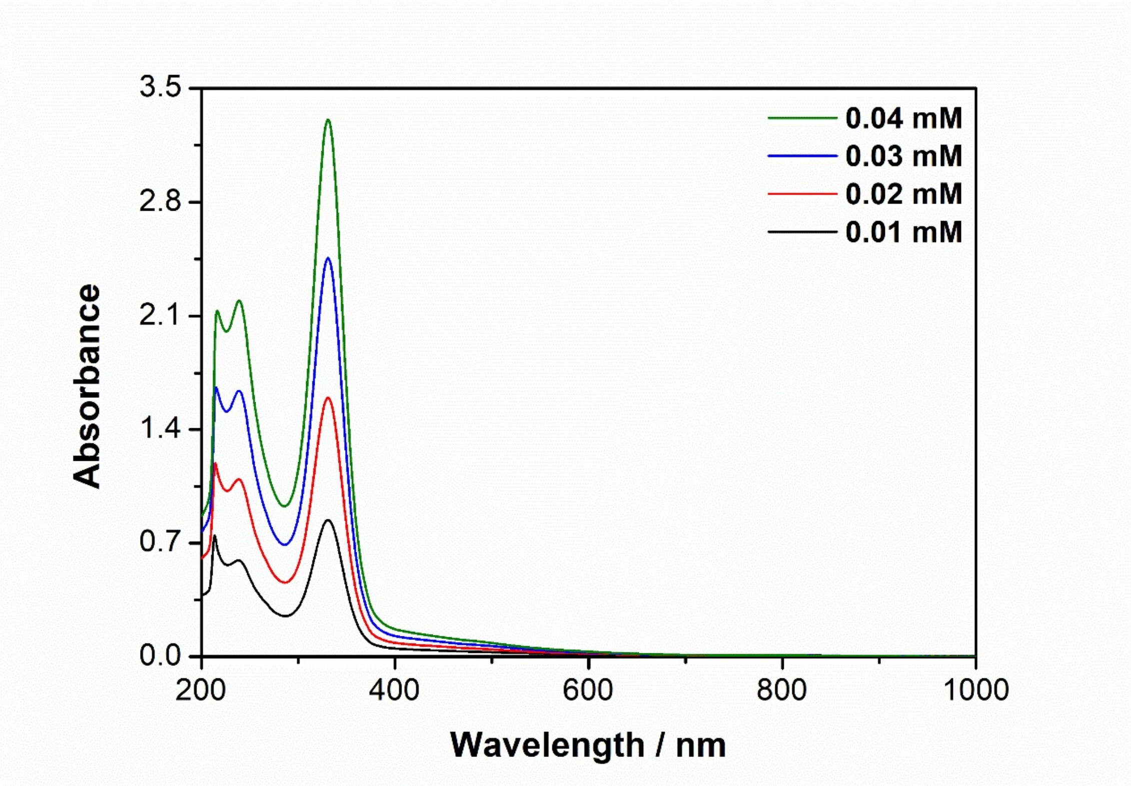

Figure S48. UV/vis/NIR spectrum of **1**$_{Dy}$ in hexane at different concentrations. Significant absorptions occur at λ = 216, 239 and 331 nm.

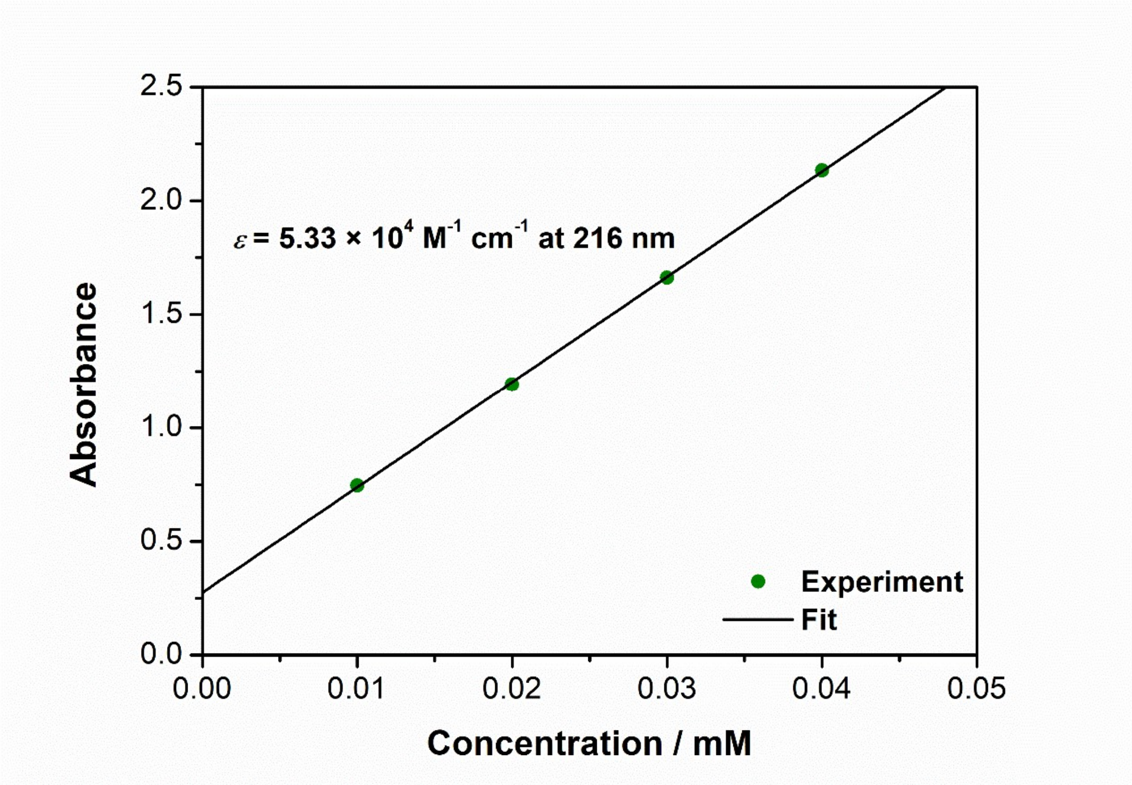

Figure S49. Plot of absorbance versus concentration for $\mathbf{1_{Dy}}$ at 216 nm in the UV/vis/NIR spectrum. The green points are from the UV/vis/NIR spectrum. The solid black line is the best fit to the data to extract the extinction coefficient.

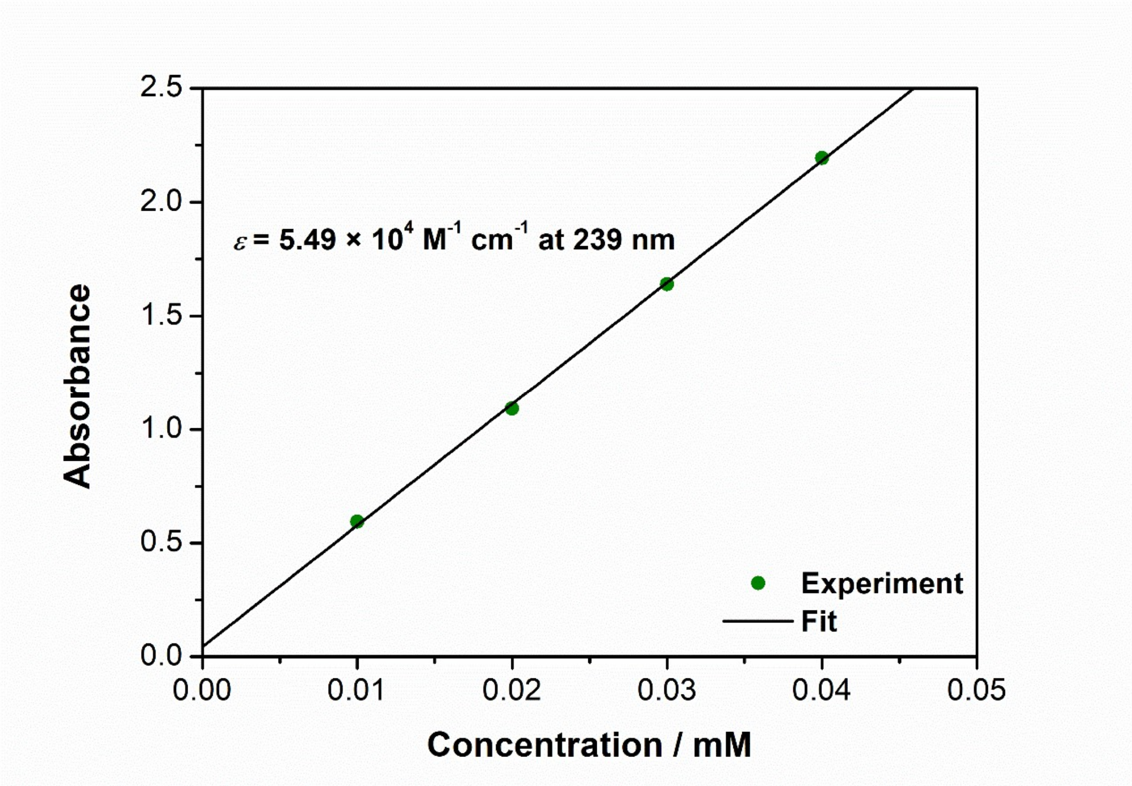

Figure S50. Plot of absorbance versus concentration for $\mathbf{1_{Dy}}$ at 239 nm in the UV/vis/NIR spectrum. The green points are from the UV/vis/NIR spectrum. The solid black line is the best fit to the data to extract the extinction coefficient.

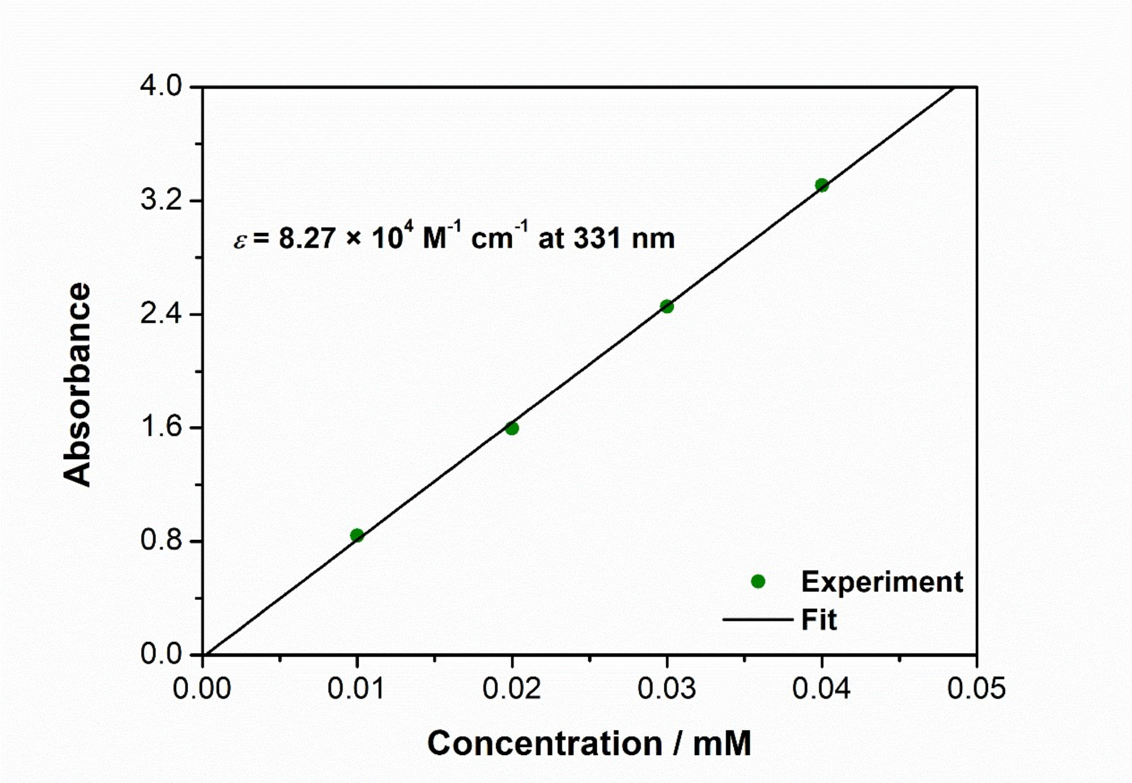

Figure S51. Plot of absorbance versus concentration for $\mathbf{1}_{Dy}$ at 331 nm in the UV/vis/NIR spectrum. The green points are from the UV/vis/NIR spectrum. The solid black line is the best fit to the data to extract the extinction coefficient.

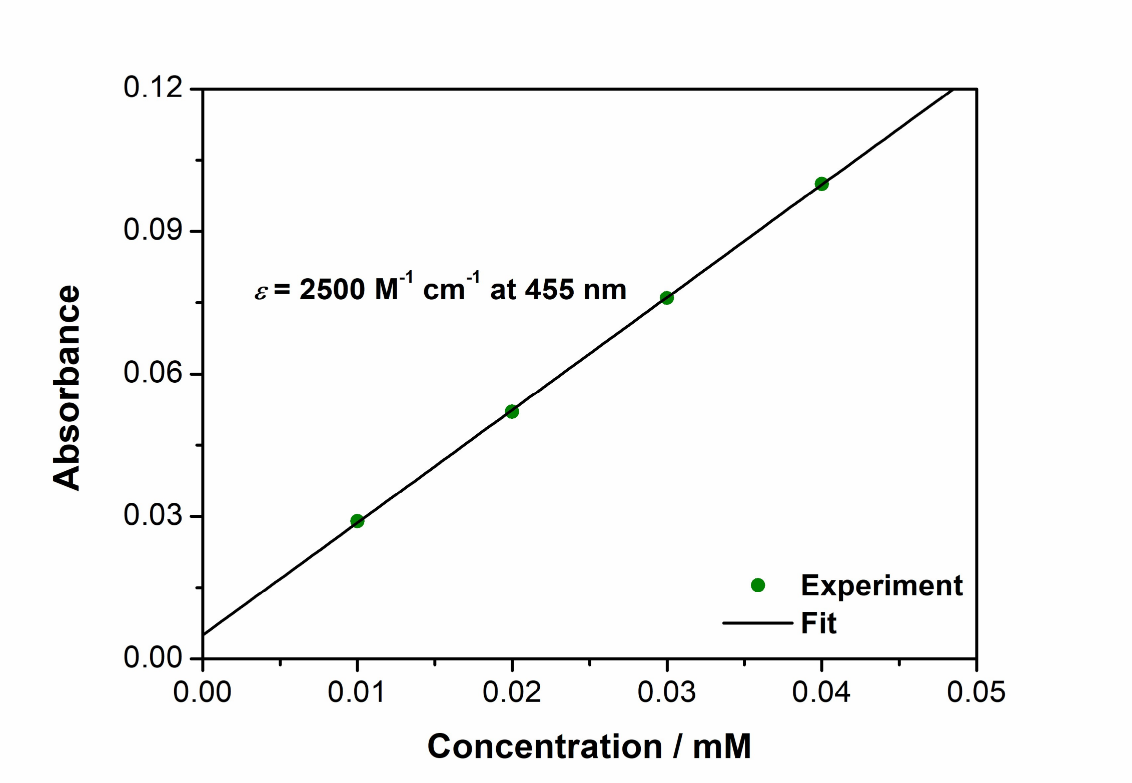

Figure S52. Plot of absorbance versus concentration for $\mathbf{1}_{Y}$ at 455 nm in the UV/vis/NIR spectrum. The green points are from the UV/vis/NIR spectrum. The solid black line is the best fit to the data to extract the extinction coefficient.

Magnetic Measurements

The samples of $[(Cp^{iPr5})_2M_2(\mu\text{-}\eta^6\text{:}\eta^6\text{-}C_6H_6)]$ (M = La, Sm, Dy, Gd) were restrained in eicosane and sealed in NMR tubes. The eicosane was melted in a water bath at 40 °C to prevent the orientation of the crystals. Direct current (DC) magnetic susceptibility and magnetization data (VSM mode) were collected using a Quantum Design MPMS3 magnetometer in cooling mode. Alternating current (AC) magnetic susceptibility measurements were performed using a Quantum Design MPMS3 magnetometer using an oscillating field of 2 Oe. Diamagnetic corrections were performed using Pascal's coefficients.[6]

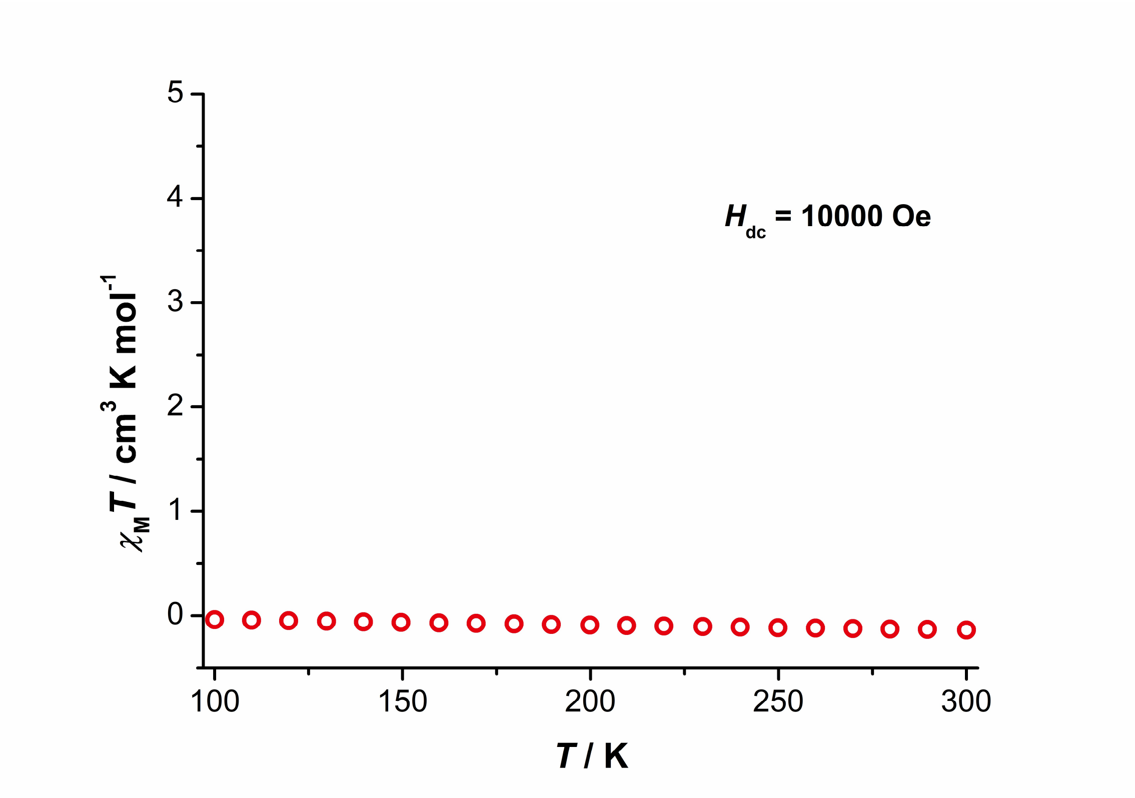

Figure S53. Plot of $\chi_M T$ versus temperature for $\mathbf{1}_{\mathbf{La}}$ in an applied field of 10 kOe.

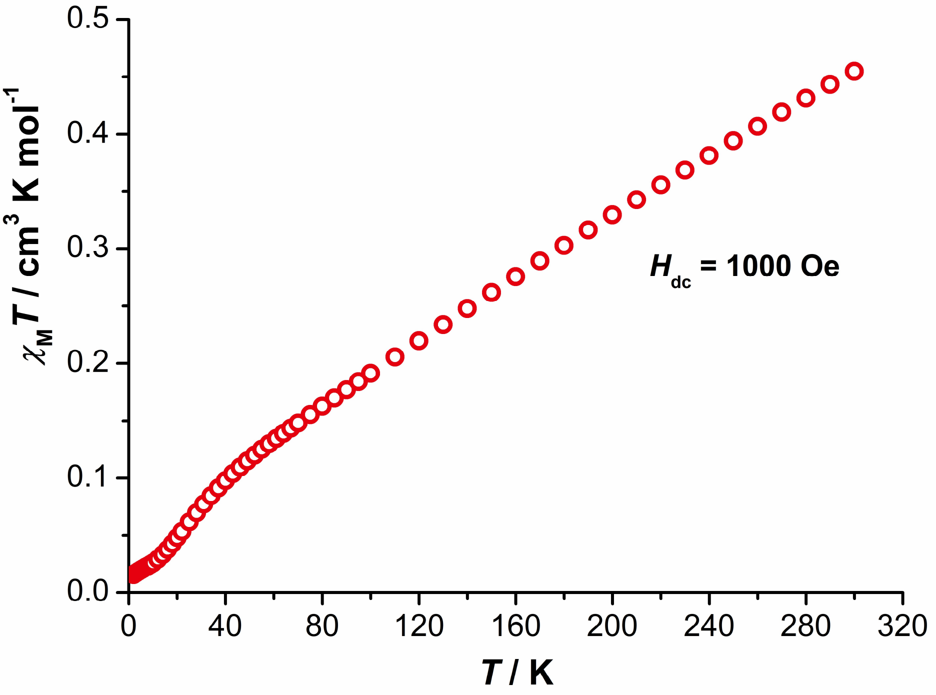

Figure S54. Plot of $\chi_M T$ versus temperature for $\mathbf{1}_{\mathbf{Sm}}$ in an applied field of 1 kOe. $\chi_M T$(300 K) = 0.455 cm^3 K mol^{-1}, $\chi_M T$(2 K) = 0.016 cm^3 K mol^{-1}.

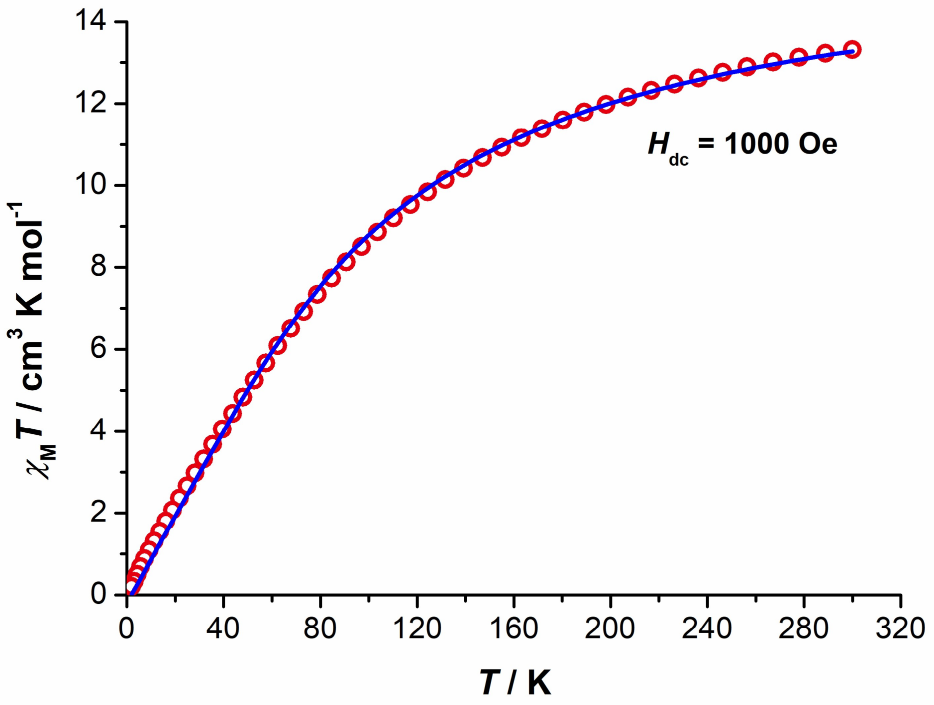

Figure S55. Plot of $\chi_M T$ versus temperature for $\mathbf{1}_{\mathbf{Gd}}$ in an applied field of 1 kOe. $\chi_M T$(300 K) = 13.31 cm^3 K mol^{-1}, $\chi_M T$(2 K) = 0.21 cm^3 K mol^{-1}. The susceptibility data were using the isotropic spin Hamiltonian ($\hat{H} = -J\hat{S}_1 \cdot \hat{S}_2 + g\mu_B \hat{S}_z H$), where $S_1 = S_2 = {}^7/_2$, and the best fitting (blue line) results give J = –3.35(1) cm^{-1} and g = 2.01(1).

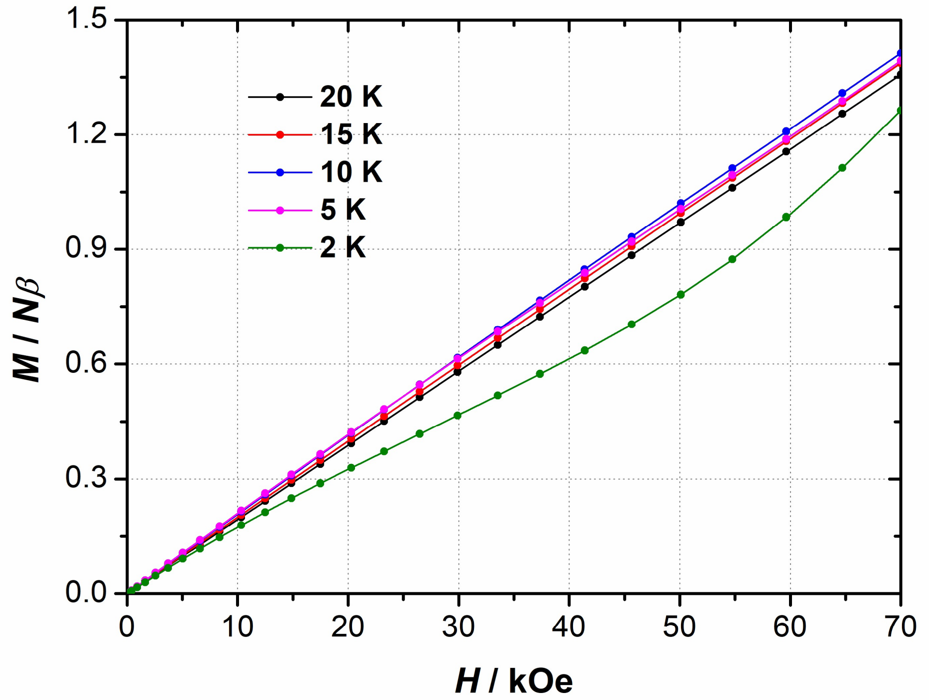

Figure S56. Field dependence of the magnetization (*M*) at 2 K (green), 5 K (magenta), 10 K (blue), 15 K (red) and 20 K (black) for $\mathbf{1}_{\mathbf{Gd}}$. Solid lines are a guide to the eye. *M* = 1.26 Nβ at 2 K and 70 kOe.

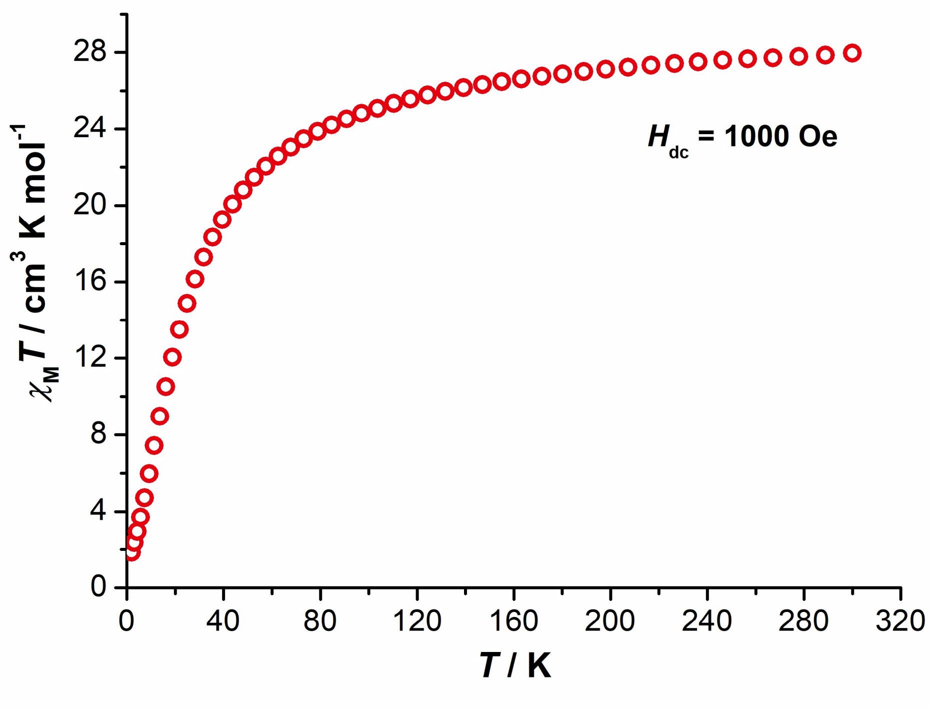

Figure S57. Plot of $\chi_M T$ versus temperature for $\mathbf{1}_{\mathbf{Dy}}$ in an applied field of 1 kOe. $\chi_M T$(300 K) = 27.96 cm^3 K mol^{-1}, $\chi_M T$(2 K) = 1.87 cm^3 K mol^{-1}.

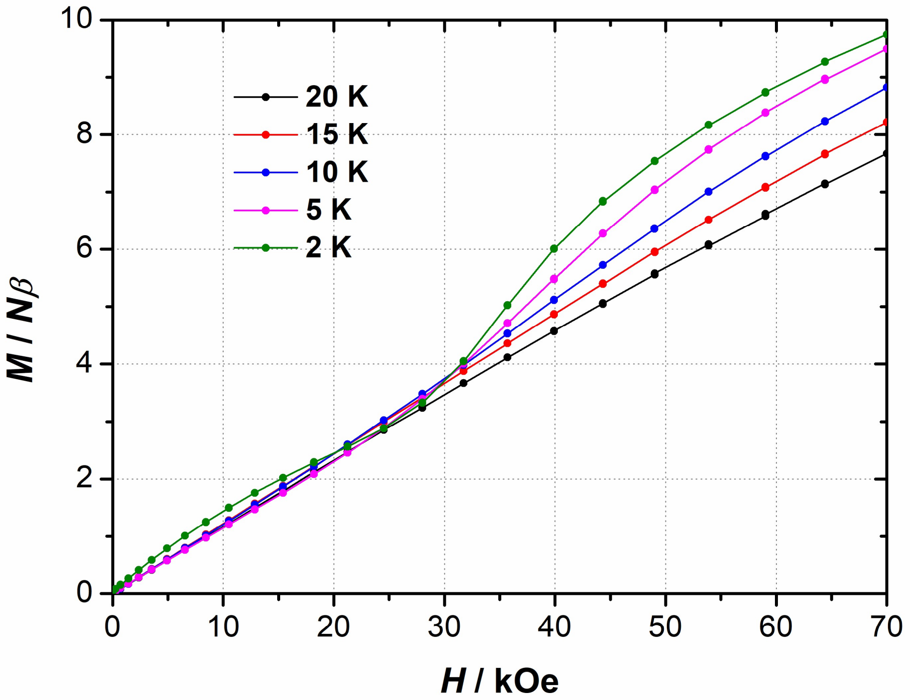

Figure S58. Field dependence of the magnetization (*M*) at 2 K (green), 5 K (magenta), 10 K (blue), 15 K (red) and 20 K (black) for $\mathbf{1}_{\mathbf{Dy}}$. Solid lines are a guide to the eye. *M* = 9.75 Nβ at 2 K and 70 kOe.

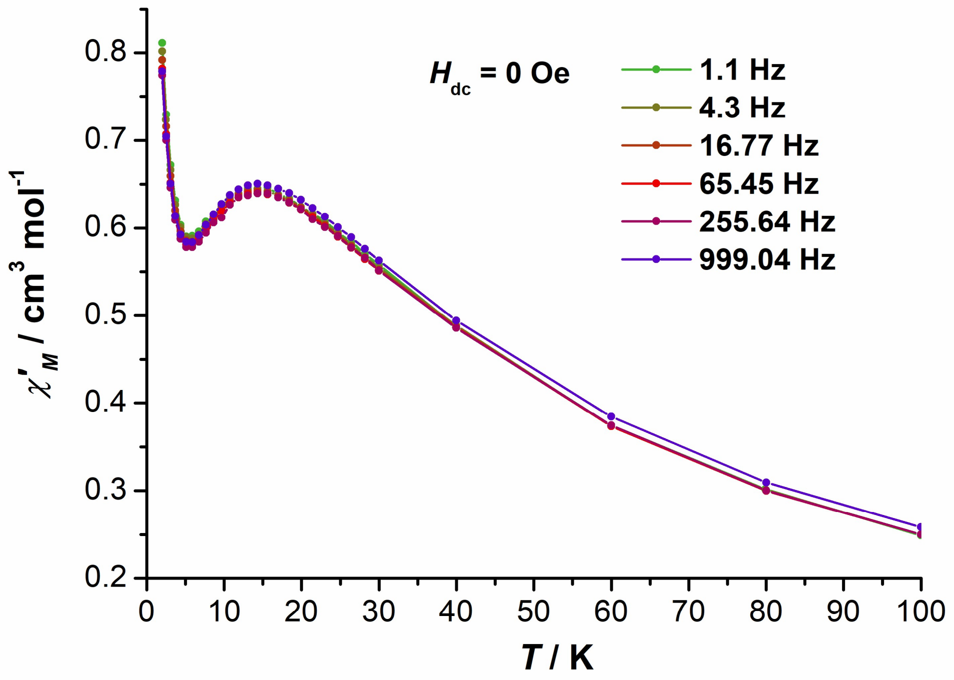

Figure S59. Temperature dependence of the in-phase (χ') AC susceptibility components for $\mathbf{1_{Dy}}$ at different frequencies in zero DC field.

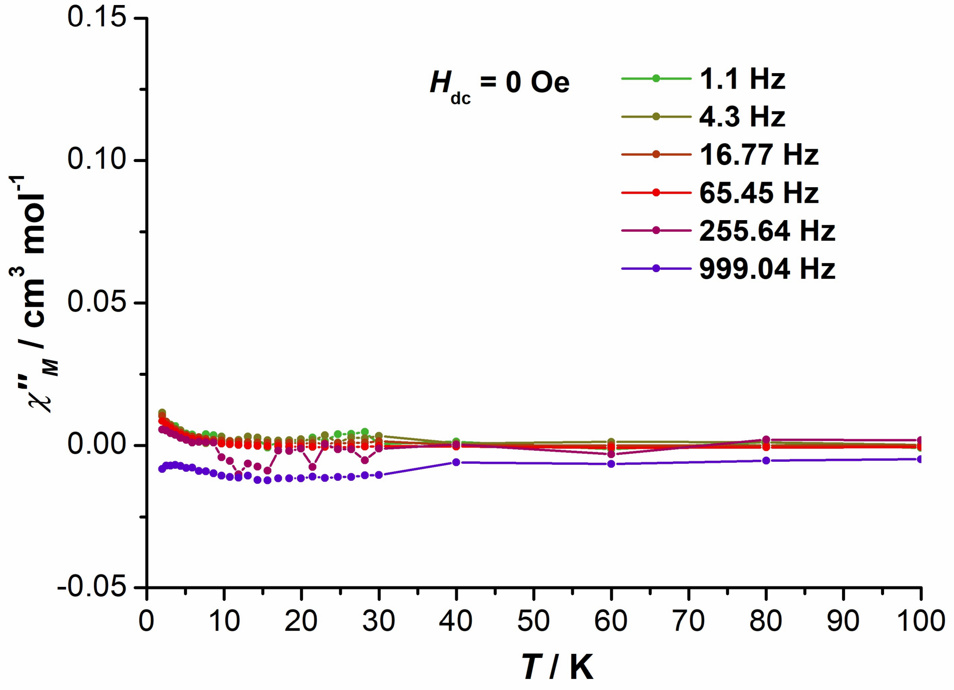

Figure S60. Temperature dependence of the out-of-phase (χ'') AC susceptibility components for $\mathbf{1_{Dy}}$ at different frequencies in zero DC field.

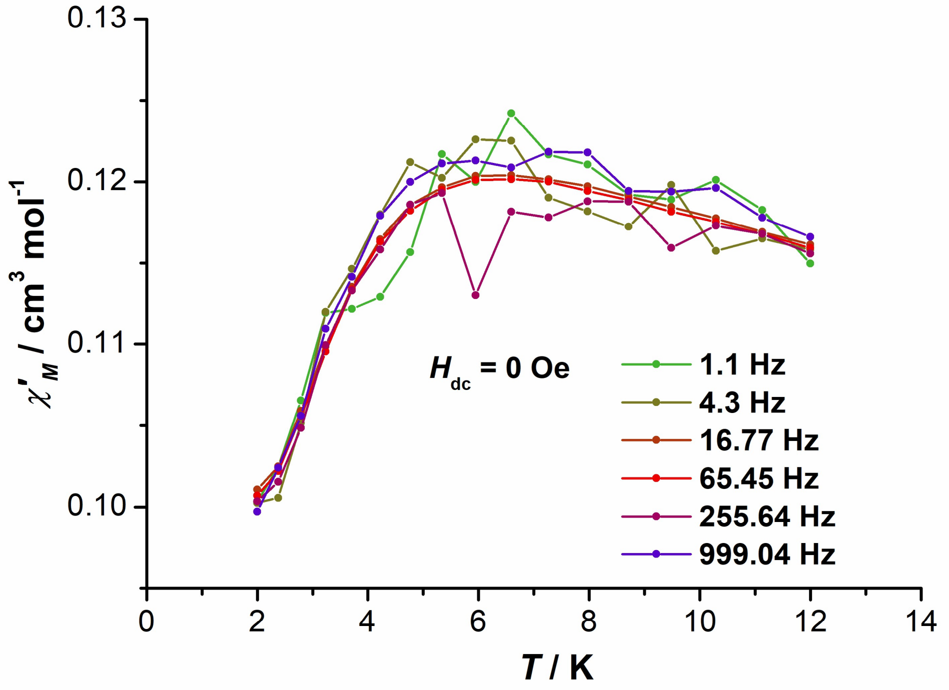

Figure S61. Temperature dependence of the in-phase (χ') AC susceptibility components for $\mathbf{1}_{Gd}$ at different frequencies in zero DC field.

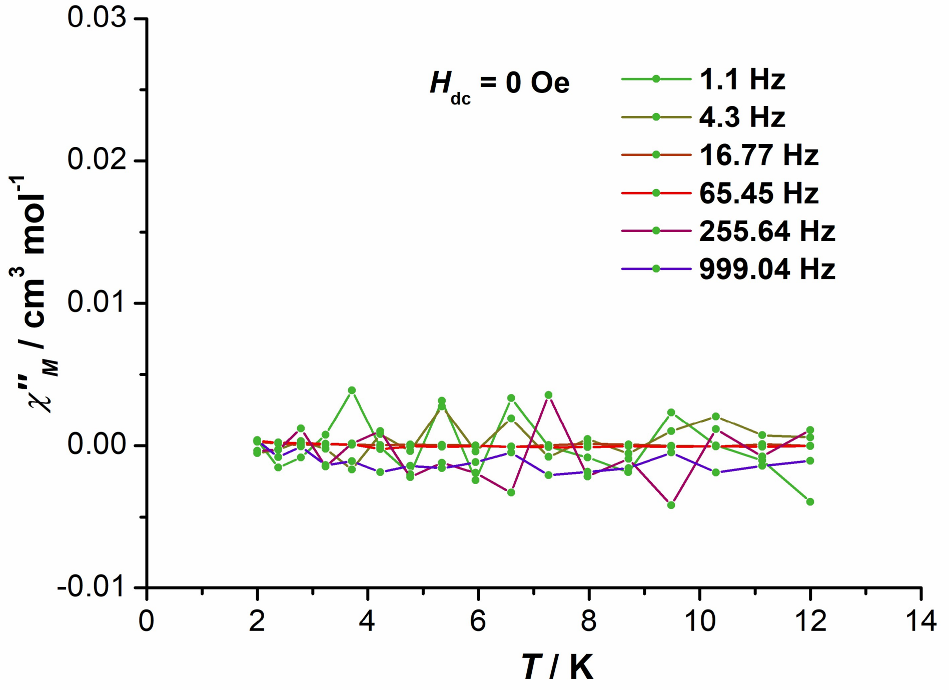

Figure S62. Temperature dependence of the out-of-phase (χ'') AC susceptibility components for $\mathbf{1}_{Gd}$ at different frequencies in zero DC field.

Computational details

DFT and TD-DFT Calculations

DFT calculations were performed on the coordinates obtained from the X-ray structure using the ORCA 5.0.2 software package.[7,8] The position of hydrogen atoms was optimized at the DFT level using the pure GGA PBE exchange-correlation functional, keeping the positions of other atoms constant.[9,10] We used the TPSSh functional[11] and relativistic effects were included with the Douglas-Kroll-Hess Hamiltonian (DKH), together with the scalar relativistic contracted version of the basis functions def2-QZVP for lanthanum, and def2-TZVP for yttrium, carbon and hydrogen.[12,13] The SARC/J auxiliary basis set[14] and RIJCOSX approximation[15,16] with TightSCF convergence threshold were also used throughout. TD-DFT calculations were performed with 50 excited states for both complexes with a CPCM implicit solvent model for hexane.[17,18] To probe the aromaticity, the Nucleus Independent Chemical Shift (NICS) calculations were performed with the whole molecule at the indicated positions using the b3lyp/def-TZVP/6-31g* level of theory in Gaussian16.[19,20]

BS-DFT Calculations

For $\mathbf{1_{Gd}}$, we performed DFT calculations in combination with the broken-symmetry (BS) approach using the ORCA 5.0.2 software package.[7,8] Relativistic effects were included with the Douglas-Kroll-Hess Hamiltonian (DKH), together with the scalar relativistic contracted version of the basis functions def2-QZVP for gadolinium and def2-TZVP for carbon and hydrogen atoms. In these calculations, the well-known B3LYP functional was employed to extract the isotropic exchange coupling using the method of Yamaguchi (eqn. S1), where the exchange coupling is determined from the energies (E) and spin expectation values ($<S^2>$) of the triplet and broken-symmetry singlet states based on the spin Hamiltonian $H = -2J \cdot S_{\mathrm{Gd1}} \cdot S_{\mathrm{Gd2}}$.

$$J = \frac{-(E_T - E_{BSS})}{<S^2>_T - <S^2>_{BSS}} \qquad \text{eqn. S1}$$

Multireference Calculations

Calculations were carried out on $\mathbf{1_{Dy}}$ using the coordinates obtained from the X-ray structure using ORCA 5.0.2.[7,8] The position of hydrogen atoms was optimized at the DFT level using the pure GGA PBE exchange correlation functional,[9,10] keeping the position of other atoms constant. To avoid the convergence problem, we replaced Dy^{3+} with Y^{3+} during the optimizations. The def2-TZVP basis sets with effective core potential (ECP) were used to treat the core electrons of yttrium throughout the DFT calculations.[21,22] Calculations were of the CASSCF/QDPT type and the DKH (Douglas-Kroll-Hess) Hamiltonian was used throughout the calculations to consider relativistic effects. We employed the SARC2-DKH-QZVP basis set for Dy^{3+}, and all other atoms were treated with the DKH-def2-SVP basis set in combination with 'AutoAux' auxiliary basis set.[13,23] The active space CAS(9,7) was constructed from nine electrons in seven f-orbitals. In the configuration interaction procedure, 21 sextets, 128 quartets, and 130 doublets were computed for all the complexes. To consider the spin-orbit coupling, we also used the quasi-degenerate perturbation theory (QDPT) approach using SA-CASSCF wave functions with the spin–orbit mean field (SOMF-IX) operator.[24] The SINGLE_ANISO module[25] as implemented in ORCA was used to compute the *g*-tensors and crystal field parameters of the low-lying excited state using previously calculated spin-orbit states. To represent the 4f orbital splitting, we also performed ab initio ligand field theory (AILFT) calculations at both CASSCF/NEVPT2 levels of theory for $\mathbf{1_{Dy}}$.[26]

To quantify the exchange interaction in $\mathbf{1_{Dy}}$, we simulated the molar magnetic susceptibility and magnetization data using the PHI software[27] and the Hamiltonian in equation S2.

$$\hat{H} = -2J_{\mathrm{tot}} \cdot (S_{\mathrm{Dy1}} \cdot S_{\mathrm{Dy2}}) + B_0^2 C_0^2 + B_0^4 C_0^4 + B_0^6 C_0^6 + zJ' \qquad \text{eqn. S2}$$

Crystal field parameters obtained from the ab initio calculation (Table S20) and an intermolecular interaction ($zJ' = -0.001$ $\mathrm{cm^{-1}}$) were included. The best simulation of susceptibility and magnetization (Figure S20) gives $J_{\mathrm{tot}} = -0.78$ $\mathrm{cm^{-1}}$.

Table S12. Excitation wavelengths (λ) and oscillator strengths (*f*) in length representation for **1**$_{Y}$.

**Excitation**	λ **/ nm**	***f***	**Assignment (major contribution)**
214a → 216a	558	0.00058	HOMO→LUMO+1
213a → 216a	546	0.00006	HOMO-1→LUMO+1
214a → 217a<br>214a → 218a	507	0.00002	HOMO→LUMO+2<br>HOMO→LUMO+3
214a → 219a	348	0.0005	HOMO→LUMO+4
212a → 215a<br>211a -> 215a	316	0.0008	HOMO-2→LUMO<br>HOMO-3→LUMO
213a → 218a<br>214a → 217a	315	0.2065	HOMO-1→LUMO+3<br>HOMO→LUMO+2
213a → 224a<br>213a → 225a	284	0.0050	HOMO-1→LUMO+9<br>HOMO-1→LUMO+10
209a → 218a<br>210a → 217a	263	0.00226	HOMO-5→LUMO+3<br>HOMO-4→LUMO+3
213a → 228a<br>214a → 229a	253	0.00203	HOMO-1→LUMO+13<br>HOMO→LUMO+14
213a → 231a<br>214a → 232a	242	0.00050	HOMO-1→LUMO+16<br>HOMO→LUMO+17
214a -> 233a	236	0.00013	HOMO→LUMO+18

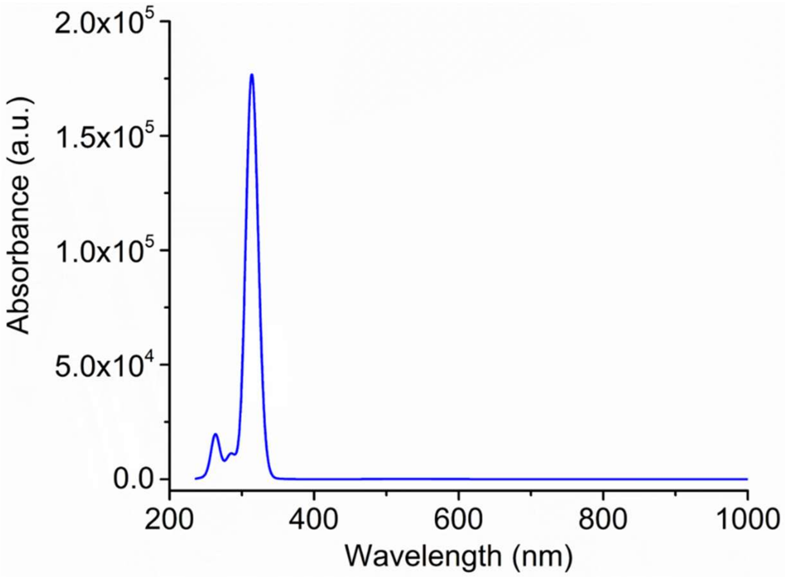

Figure S63. Calculated UV-vis spectrum of **1**$_{Y}$.

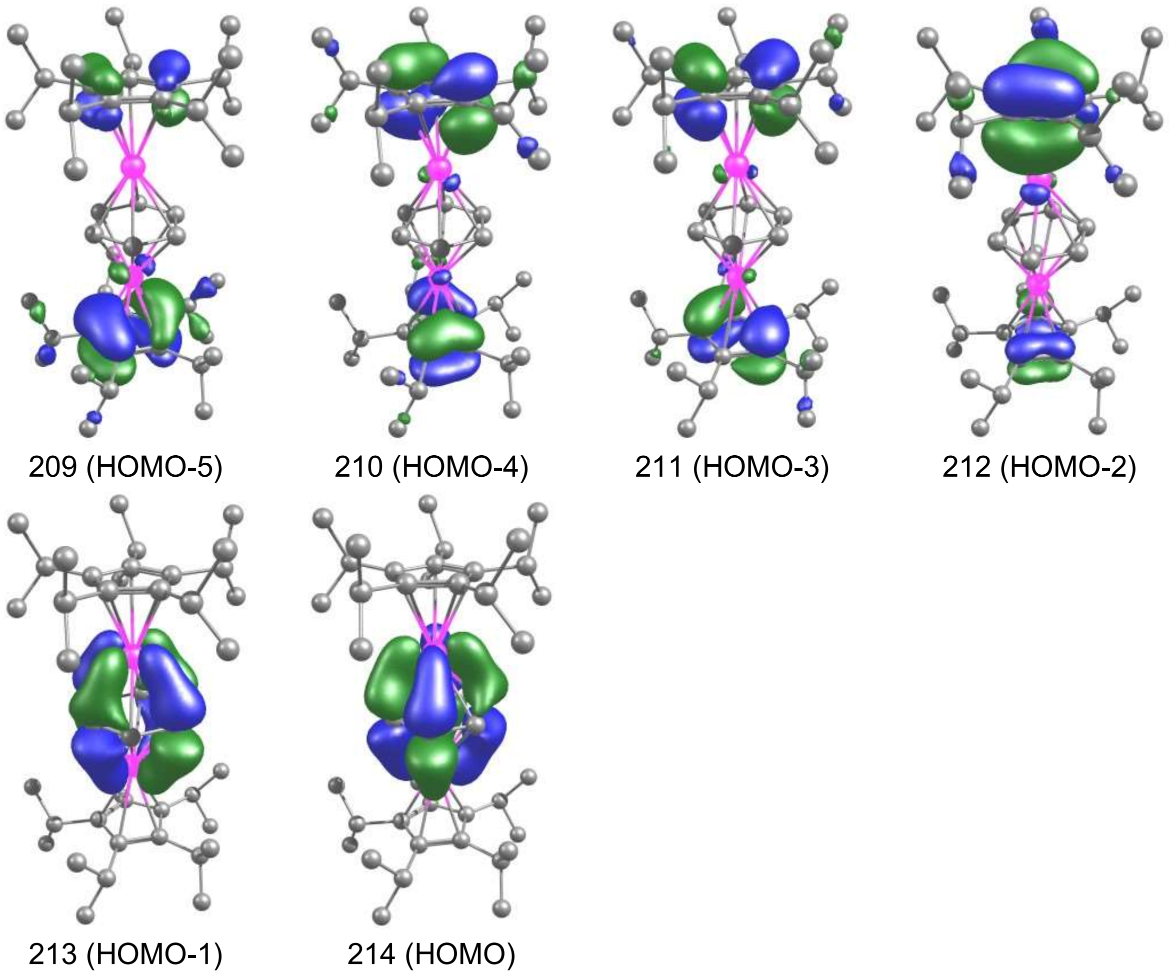

Figure S64. Selected occupied MOs for $\mathbf{1}_{\mathbf{Y}}$ (isosurface value 0.04 a.u.).

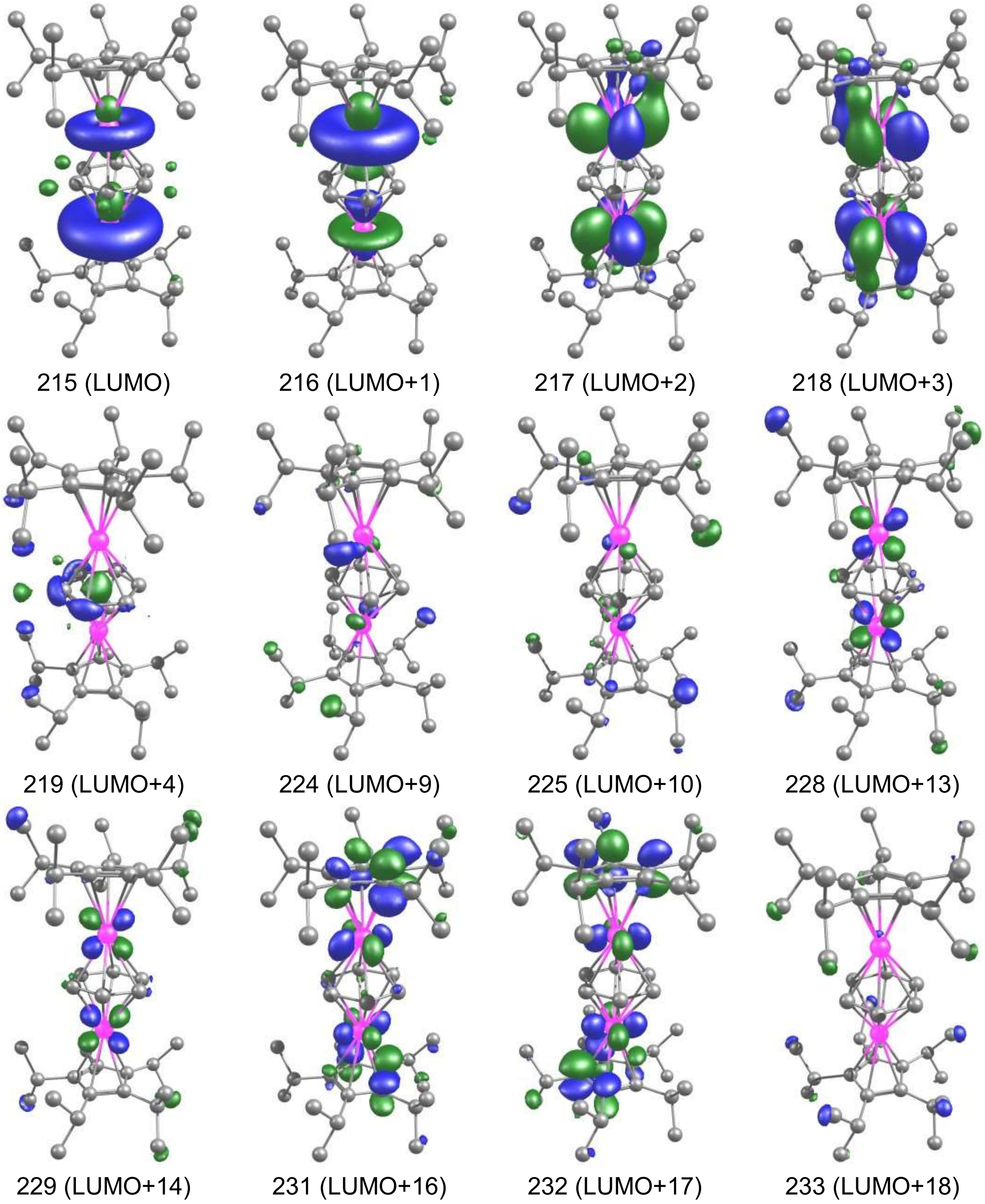

Figure S65. Selected unoccupied MOs for **1**$_{\mathbf{Y}}$ (isosurface value 0.04 a.u.).

Table S13. Excitation wavelengths (λ) and oscillator strengths (*f*) in length representation for $\mathbf{1}_{\mathbf{La}}$.

Excitation	λ / nm	*f*	Assignment (major contribution)
232a → 234a<br>231a →236a<br>232a → 235a	581	0.00071	HOMO→LUMO+1<br>HOMO-1→LUMO+3<br>HOMO→LUMO+2
231a → 235a<br>231a → 236a<br>232a → 234a	551	0.00007	HOMO-1→LUMO+2<br>HOMO-1→LUMO+3<br>HOMO→LUMO+1
232a → 235a<br>232a → 236a<br>232a → 234a	543	0.00005	HOMO→LUMO+2<br>HOMO→LUMO+3<br>HOMO→LUMO+1
232a → 240a<br>231a → 241a	454	0.00028	HOMO→LUMO+7<br>HOMO-1→LUMO+8
231a → 241a<br>231a → 240a<br>232a → 240a	446	0.00126	HOMO-1→LUMO+8<br>HOMO-1→LUMO+7<br>HOMO→LUMO+7
231a → 239a<br>231a → 234a	436	0.15365	HOMO-1→LUMO+6<br>HOMO-1→LUMO+1
231a → 246a<br>232a → 246a	386	0.01558	HOMO-1→LUMO+13<br>HOMO→LUMO+13
231a → 253a<br>232a → 251a	352	0.01722	HOMO-1→LUMO+20<br>HOMO→LUMO+18
231a -> 251a<br>232a -> 246a	345	0.08561	HOMO-1→LUMO+18<br>HOMO→LUMO+13

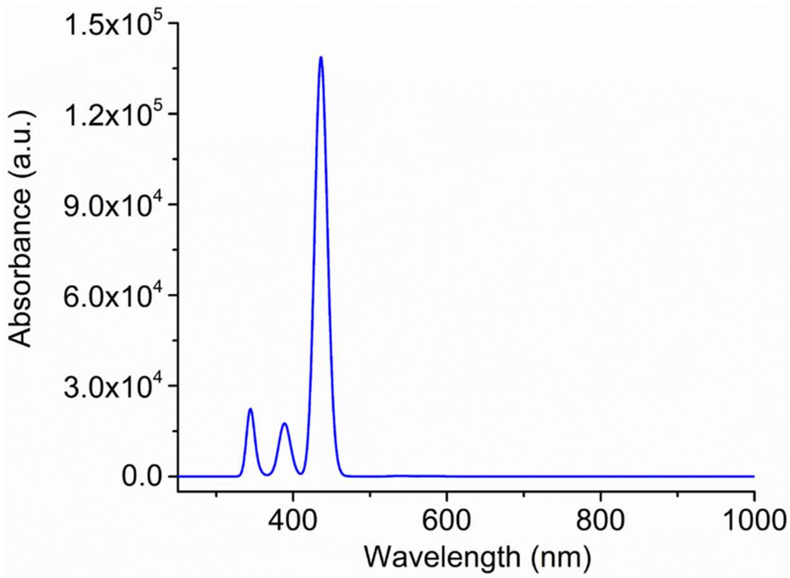

Figure S66. Calculated UV-vis spectrum of $\mathbf{1}_{\mathbf{La}}$.

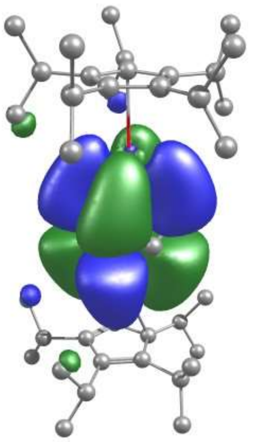
231 (HOMO-1)

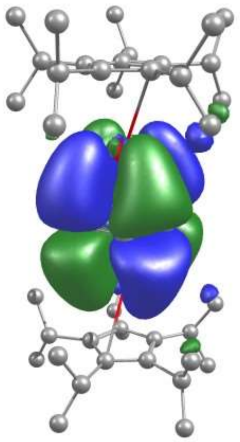
232 (HOMO)

Figure S67. Selected occupied MOs for $\mathbf{1}_{\mathbf{La}}$ (isosurface value 0.02 a.u.).

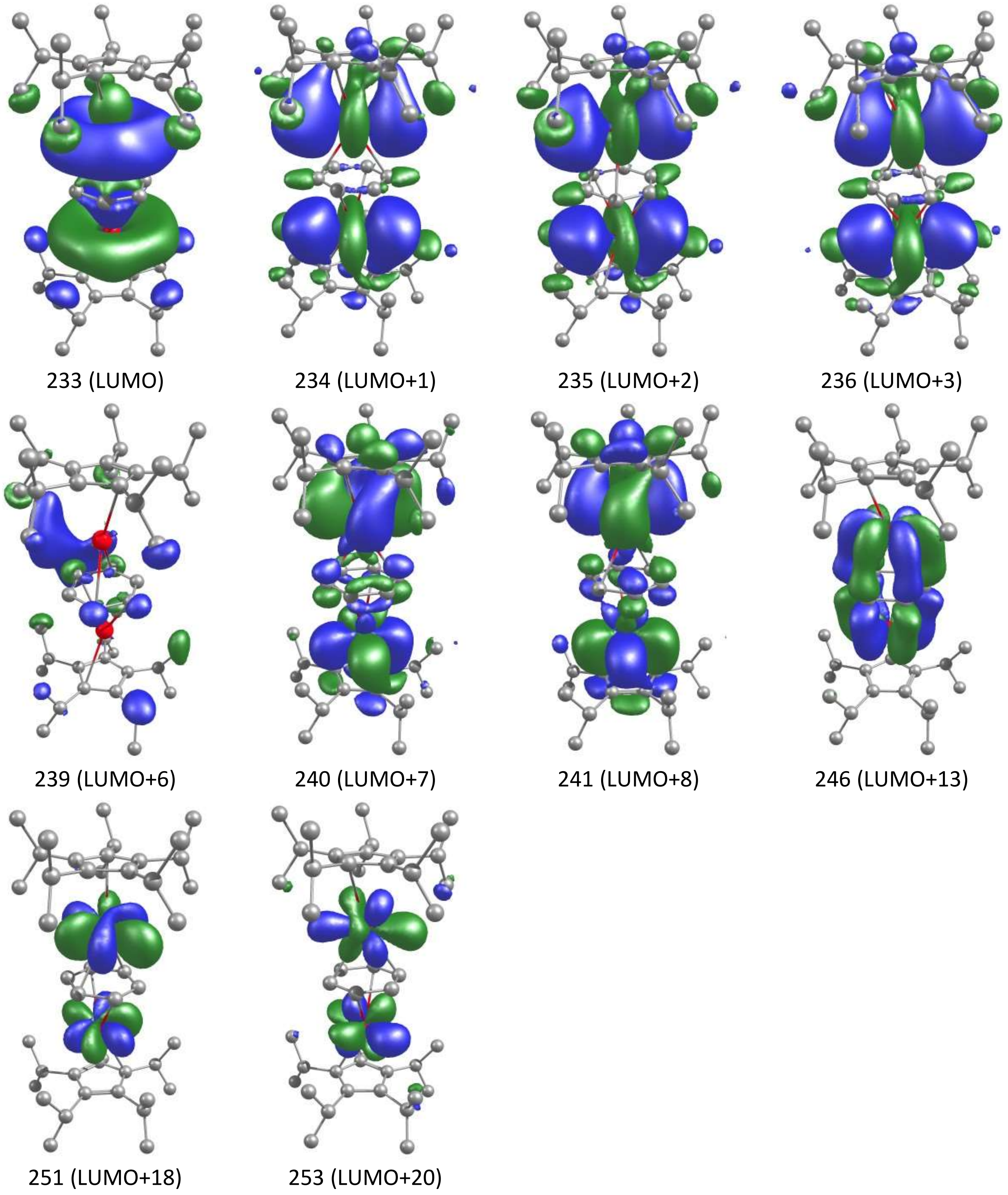

Figure S68. Selected unoccupied MOs for $\mathbf{1_{La}}$ (isosurface value 0.02 a.u.).

Table S14. Energy of the high-spin (HS) and broken-symmetry (BS) states for $\mathbf{1}_{Gd}$.

Spin state	Energy / hartree	$<S^2>$	Coupling constant (J_{ex}) / cm^{-1}
HS	-24328.628762	56.0378	–2.86
BS	-24328.629400	7.0774	

Table S15. Spin density for the gadolinium and benzene carbon atoms in the high-spin (HS) and broken-symmetry (BS) state of $\mathbf{1}_{Gd}$.

Selected Atoms	HS	BS
Gd	7.086008	7.245467
Gd	7.094801	-7.248832
C	-0.028362	-0.000058
C	-0.027726	0.000707
C	-0.028152	-0.000083
C	-0.028498	0.000212
C	-0.028886	0.000159
C	-0.029149	0.001151

Table S16. Ab initio calculated low-lying spin-orbit energy states for $\mathbf{1_{Dy}}$.

**Energy / cm^{-1}**
0.000
0.000
61.3415
61.3415
116.7193
116.7193
176.4089
176.4089
206.1734
206.1734
307.8012
307.8012
335.2000
335.2000
400.1325
400.1325

Table S17. Computed energy of the KDs, g, and wavefuctions composition for a Dy^{3+} centre in $\mathbf{1_{Dy}}$.

KD	*E* / cm^{-1}	g_x	g_y	g_z	Wavefunction composition
1	0.000	0.1900	0.447	17.15	62.1%|±15/2>, 9.30%|±13/2>, 15.80%|±11/2>, 0.70%|±9/2>, 8.50%|±7/2>, 0.70%|±5/2>, 1.03%|±3/2>, 1.80%|±1/2>
2	61.342	0.8702	0.933	13.14	4.90%|±15/2>, 38.10%|±13/2>, 3.60%|±11/2>, 28.50%|±9/2>, 0.70%|±7/2>, 14.20%|±5/2>, 8.90%|±3/2>, 1.20%|±1/2>
3	116.719	0.772	1.284	13.02	27.50%|±15/2>, 4.0%|±13/2>, 14.60%|±11/2>, 0.60%|±9/2>, 13.40%|±7/2>, 10.90%|±5/2>, 5.80%|±3/2>, 23.0%|±1/2>
4	176.409	0.208	0.701	15.59	1.80%|±15/2>, 33.40%|±13/2>, 11.30%|±11/2>, 11.10%|±9/2>, 9.0%|±7/2>, 0.6%|±5/2>, 17.90%|±3/2>, 14.80%|±1/2>
5	206.173	0.916	2.963	9.33	2.30%|±15/2>, 3.70%|±13/2>, 18.50%|±11/2>, 3.10%|±9/2>, 6.60%|±7/2>, 10.0%|±5/2>, 29.70%|±3/2>, 2.6.10%|±1/2>
6	307.801	6.175	5.019	3.35	0.80%|±15/2>, 6.40%|±13/2>, 9.60%|±11/2>, 5.60%|±9/2>, 17.20%|±7/2>, 34.30%|±5/2>, 18.90%|±3/2>, 17.10%|±1/2>
7	335.200	0.054	0.123	19.48	0.20%|±15/2>, 0.10%|±13/2>, 4.30%|±11/2>, 12.90%|±9/2>, 23.40%|±7/2>, 26.40%|±5/2>, 20.40%|±3/2>, 12.2%|±1/2>
8	400.132	0.936	4.607	15.74	0.30%|±15/2>, 4.90%|±13/2>, 22.50%|±11/2>, 37.50%|±9/2>, 21.20%|±7/2>, 2.90%|±5/2>, 7.20%|±3/2>, 3.70%|±1/2>

Table S18. Transition magnetic moment matrix elements (in Bohr magneton) for $\mathbf{1_{Dy}}$.

Climbing Transition			Crossing Transition		
Initial KD	Final KD	Magnitude	Initial KD	Final KD	Magnitude
1	2	2.1454	1	1	0.10619
1	3	1.4523	1	2	0.18682
1	4	0.52399	1	3	0.12296
1	5	0.36668	1	4	0.13954
1	6	0.9990	1	5	0.16590
1	7	0.11535	1	6	0.08682
1	8	0.07358	1	7	0.01174
2	3	2.1174	1	8	0.030132
2	4	1.3359	2	2	0.30892
2	5	1.8649	2	3	0.43908
2	6	0.24034	2	4	0.25715
2	7	0.61304	2	5	0.21976
2	8	0.08155	2	6	0.14764
3	4	1.9903	2	7	0.38444
3	5	2.3995	2	8	0.03422
3	6	0.66404	3	3	0.40863
3	7	0.62663	3	4	0.44098
3	8	0.15660	3	5	0.43875
4	5	1.4328	3	6	0.42493
4	6	1.1889	3	7	0.06231
4	7	1.3954	3	8	0.07882
4	8	0.20415	4	4	0.21215
5	6	3.0991	4	5	0.85426
5	7	0.98579	4	6	0.78529
5	8	0.57306	4	7	0.069278
6	7	0.35063	4	8	0.16671
6	8	2.1027	5	5	1.2024
7	8	0.2567	5	6	1.90830
			5	7	0.32080
			5	8	0.25964
			6	6	1.6595
			6	7	0.39151
			6	8	1.9149
			7	7	0.06405
			7	8	0.47951
			8	8	1.0034

Table S19. Crystal-field parameters B_q^k for **1**$_{\mathbf{Dy}}$.

k	*q*	
	-2	-0.6136E-02
	-1	-0.2620E-01
2	0	-0.1034E+01
	1	-0.2029E+01
	2	0.1271E+01
	-4	-0.9174E-03
	-3	-0.5457E-02
	-2	-0.1516E-02
	-1	0.3608E-03
4	0	-0.3348E-02
	1	0.3123E-02
	2	0.2378E-01
	3	0.7990E-01
	4	0.1030E-01
	-6	0.4865E-04
	-5	-0.1486E-03
	-4	0.4598E-04
	-3	-0.9507E-05
	-2	0.9645E-05
	-1	-0.1845E-04
6	0	0.1142E-04
	1	0.3123E-03
	2	-0.7586E-04
	3	-0.5039E-03
	4	-0.2826E-03
	5	-0.4454E-03
	6	-0.5468E-04

The following Hamiltonian is used to calculate the crystal field parameters:

$$\hat{H}_{\mathrm{CF}} = \sum_{k=-q}^{q} B_q^k \, \hat{O}_q^k$$

Where $\hat{O}_q^k$ is the extended stevens operator, k is the rank of the ITO (2,4,6), and q is the component of the ITO and takes values of $-k, -k+1, \dots, 0, 1, \dots k$.

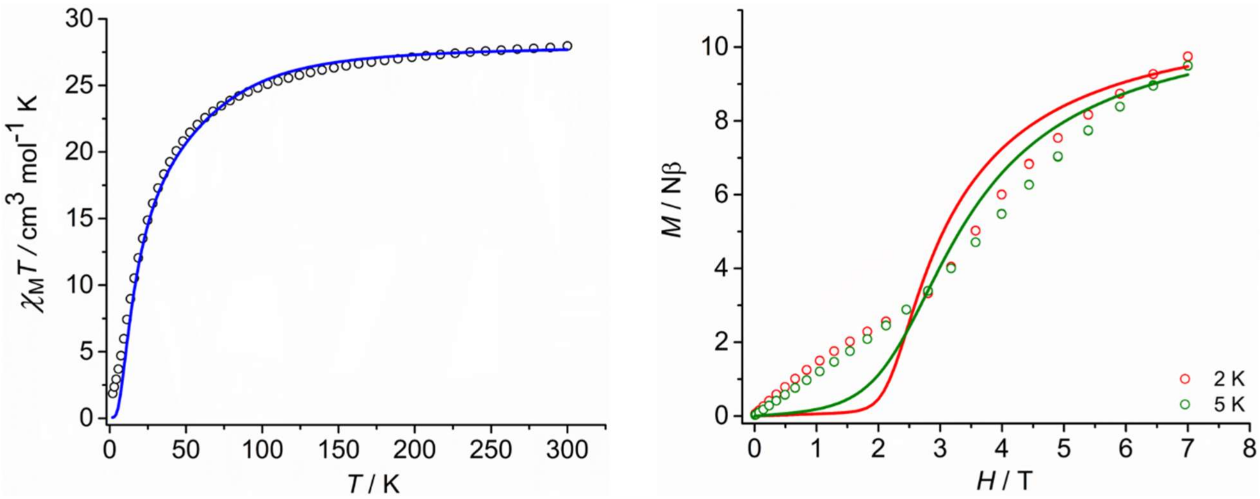

Figure S69. Experimental and simulated $\chi_M T(T)$ data (left) and $M(H)$ data (right) for $\mathbf{1}_{\mathbf{Dy}}$. The solid lines represent the simulations using equation S2 and the parameters in Table S20.

Table S20. Parameters used to simulate the magnetic properties of $\mathbf{1}_{\mathbf{Dy}}$.

***J* / cm⁻¹**	**g_J**	**CF parameters / cm⁻¹**
–0.78	1.33	$B_0^2 = -1.034$<br>$B_0^4 = -0.00335$<br>$B_0^6 = +0.00001142$

References

(1) Guo, F.-S.; Day, B. M.; Chen, Y.-C.; Tong, M.-L.; Mansikkamäki, A.; Layfield, R. A. Magnetic Hysteresis up to 80 Kelvin in a Dysprosium Metallocene Single-Molecule Magnet. *Science* **2018**, *362*, 1400–1403.

(2) Evans, W. J.; Kozimor, S. A.; Ziller, J. W.; Kaltsoyannis, N. Structure, Reactivity, and Density Functional Theory Analysis of the Six-Electron Reductant, $[(C_5Me_5)_2U]2(\mu\text{-}\eta^6\text{:}\eta^6\text{-}C_6H_6)$, Synthesized via a New Mode of $(C_5Me_5)_3M$ Reactivity. *J. Am. Chem. Soc.* **2004**, *126*, 14533–14547.

(3) Dolomanov, O. V; Bourhis, L. J.; Gildea, R. J.; Howard, J. A. K.; Puschmann, H. OLEX2: A Complete Structure Solution, Refinement and Analysis Program. *J. Appl. Crystallogr.* **2009**, *42*, 339–341.

(4) Sheldrick, G. M. A Short History of SHELX. *Acta Cryst. A* **2008**, *64*, 112–122.

(5) Sheldrick, G. M. Crystal Structure Refinement with SHELXL. *Acta Cryst. C* **2015**, *71*, 3–8.

(6) Bain, G. A.; Berry, J. F. Diamagnetic Corrections and Pascal's Constants. *J. Chem. Educ.* **2008**, *85*, 532.

(7) Neese, F. Software Update: The ORCA Program System—Version 5.0. *WIREs Comput. Mol. Sci.* **2022**, *12*, e1606.

(8) Neese, F.; Wennmohs, F.; Becker, U.; Riplinger, C. The ORCA Quantum Chemistry Program Package. *J. Chem. Phys.* **2020**, *152*, 224108.

(9) Perdew, J. P.; Burke, K.; Ernzerhof, M. Generalized Gradient Approximation Made Simple. *Phys. Rev. Lett.* **1996**, *77*, 3865–3868.

(10) Perdew, J. P.; Burke, K.; Ernzerhof, M. Generalized Gradient Approximation Made Simple. *Phys. Rev. Lett.* **1997**, *78*, 1396.

(11) Tao, J.; Perdew, J. P.; Staroverov, V. N.; Scuseria, G. E. Climbing the Density Functional Ladder: Nonempirical Meta--Generalized Gradient Approximation Designed for Molecules and Solids. *Phys. Rev. Lett.* **2003**, *91*, 146401.

(12) Aravena, D.; Neese, F.; Pantazis, D. A. Improved Segmented All-Electron Relativistically Contracted Basis Sets for the Lanthanides. *J. Chem. Theory. Comput.* **2016**, *12*, 1148–1156.

(13) Chmela, J.; Harding, M. E. Optimized Auxiliary Basis Sets for Density Fitted Post-Hartree–Fock Calculations of Lanthanide Containing Molecules. *Mol. Phys.* **2018**, *116*, 1523–1538.

(14) Rolfes, J. D.; Neese, F.; Pantazis, D. A. All-Electron Scalar Relativistic Basis Sets for the Elements Rb–Xe. *J. Comput. Chem.* **2020**, *41*, 1842–1849.

(15) Izsák, R.; Neese, F. An Overlap Fitted Chain of Spheres Exchange Method. *J. Chem. Phys.* **2011**, *135*, 144105.

(16) Neese, F.; Wennmohs, F.; Hansen, A.; Becker, U. Efficient, Approximate and Parallel Hartree–Fock and Hybrid DFT Calculations. A 'Chain-of-Spheres' Algorithm for the Hartree–Fock Exchange. *Chem. Phys.* **2009**, *356*, 98–109.

(17) Barone, V.; Cossi, M. Quantum Calculation of Molecular Energies and Energy Gradients in Solution by a Conductor Solvent Model. *J. Phys. Chem. A* **1998**, *102*, 1995–2001.

(18) Cossi, M.; Rega, N.; Scalmani, G.; Barone, V. Energies, Structures, and Electronic Properties of Molecules in Solution with the C-PCM Solvation Model. *J. Comput. Chem.* **2003**, *24*, 669–681.

(19) Frisch, M. J.; Trucks, G. W.; Schlegel, H. B.; Scuseria, G. E.; Robb, M. A.; Cheeseman, J. R.; Scalmani, G.; Barone, V.; Petersson, G. A.; Nakatsuji, H.; Li, X.; Caricato, M.; Marenich, A.; Bloino, J.; Janesko, B. G.; Gomperts, R.; Mennucci, B.; Hratchian, H. P.; Ortiz, J. V.; Izmaylov, A. F.; Sonnenberg, J. L.; Williams-Young, D.; Ding, F.; Lipparini, F.; Egidi, F.; Goings, J.; Peng, B.; Petrone, A.; Henderson, T.; Ranasinghe, D.; Zakrzewski, V. G.; Gao, J.; Rega, N.; Zheng, G.; Liang, W.; Hada, M.; Ehara, M.; Toyota, K.; Fukuda, R.; Hasegawa, J.; Ishida, M.; Nakajima, T.; Honda, Y.; Kitao, O.; Nakai, H.; Vreven, T.; Throssell, K.; Montgomery, J. A. Jr.; Peralta, J. E.; Ogliaro, F.; Bearpark, M.; Heyd, J. J.; Brothers, E.; Kudin, K. N.; Staroverov, V. N.; Keith, T.; Kobayashi, R.; Normand, J.; Raghavachari, K.; Rendell, A.; Burant, J. C.; Iyengar, S. S.; Tomasi, J.; Cossi, M.; Millam, J. M.; Klene, M.; Adamo, C.; Cammi, R.; Ochterski, J. W.; Martin, R. L.; Morokuma, K.; Farkas, O.; Foresman, J. B.; Fox, D. J. Gaussian 09. Gaussian, Inc.: Wallingford CT 2016.

(20) Schleyer, P. von R.; Maerker, C.; Dransfeld, A.; Jiao, H.; van Eikema Hommes, N. J. R. Nucleus-Independent Chemical Shifts: A Simple and Efficient Aromaticity Probe. *J. Am. Chem. Soc.* **1996**, *118*, 6317–6318.

(21) Schäfer, A.; Horn, H.; Ahlrichs, R. Fully Optimized Contracted Gaussian Basis Sets for Atoms Li to Kr. *J. Chem. Phys.* **1992**, *97*, 2571–2577.

(22) Weigend, F.; Ahlrichs, R. Balanced Basis Sets of Split Valence, Triple Zeta Valence and Quadruple Zeta Valence Quality for H to Rn: Design and Assessment of Accuracy. *Phys. Chem. Chem. Phys.* **2005**, *7*, 3297–3305.
(23) Aravena, D.; Atanasov, M.; Neese, F. Periodic Trends in Lanthanide Compounds through the Eyes of Multireference Ab Initio Theory. *Inorg. Chem.* **2016**, *55*, 4457–4469.
(24) Ganyushin, D.; Neese, F. First-Principles Calculations of Zero-Field Splitting Parameters. *J. Chem. Phys.* **2006**, *125*, 024103.
(25) Chibotaru, L. F.; Ungur, L. Ab Initio Calculation of Anisotropic Magnetic Properties of Complexes. I. Unique Definition of Pseudospin Hamiltonians and Their Derivation. *J. Chem. Phys.* **2012**, *137*, 064112.
(26) Gil, Y.; Aravena, D. Understanding Single-Molecule Magnet Properties of Lanthanide Complexes from 4f Orbital Splitting. *Dalton Trans.* **2024**, *53*, 2207–2217.
(27) Chilton, N. F.; Anderson, R. P.; Turner, L. D.; Soncini, A.; Murray, K. S. PHI: A Powerful New Program for the Analysis of Anisotropic Monomeric and Exchange-Coupled Polynuclear d- and f-Block Complexes. *J. Comput. Chem.* **2013**, *34*, 1164–1175.
